# Gold(iii)–arene complexes by insertion of olefins into gold–aryl bonds†
†Electronic supplementary information (ESI) available: Experimental details (syntheses, analytical data and copies of ^1^H, ^13^C and ^31^P NMR spectra), crystallographic data of **2** and computational details. CCDC 1526613 and 1538015. For ESI and crystallographic data in CIF or other electronic format see DOI: 10.1039/c7sc00145b



**DOI:** 10.1039/c7sc00145b

**Published:** 2017-04-19

**Authors:** Feriel Rekhroukh, Charlie Blons, Laura Estévez, Sonia Mallet-Ladeira, Karinne Miqueu, Abderrahmane Amgoune, Didier Bourissou

**Affiliations:** a Université de Toulouse , UPS , 118 route de Narbonne , 31062 Toulouse , France . Email: amgoune@chimie.ups-tlse.fr ; Email: dbouriss@chimie.ups-tlse.fr; b CNRS , LHFA , UMR 5069 , F-31062 Toulouse , France; c CNRS , Univ Pau & Pays Adour , Institut des Sciences Analytiques et de Physico-Chimie pour l'environnement et les Matériaux , UMR 5254 , Pau , 64000 , France . Email: karinne.miqueu@univ-pau.fr; d Université Paul Sabatier , Institut de Chimie de Toulouse (FR 2599) , 118 route de Narbonne , 31062 Toulouse Cedex 9 , France

## Abstract

The synthesis and characterization of the first gold(iii)–arene complexes are described. Well-defined (P,C)-cyclometalated gold(iii)–aryl complexes were prepared and characterized by NMR spectroscopy. These complexes swiftly and cleanly reacted with norbornene and ethylene to provide cationic gold(iii)–alkyl complexes, in which the remote phenyl ring was η^2^-coordinated to gold. The interaction between the aromatic ring and the gold(iii) center was thoroughly analyzed by NMR spectroscopy, X-ray diffraction, and DFT calculations. The π–arene coordination was found to significantly influence the stability and reactivity of low coordinated gold(iii) alkyl species.

## Abstract 2

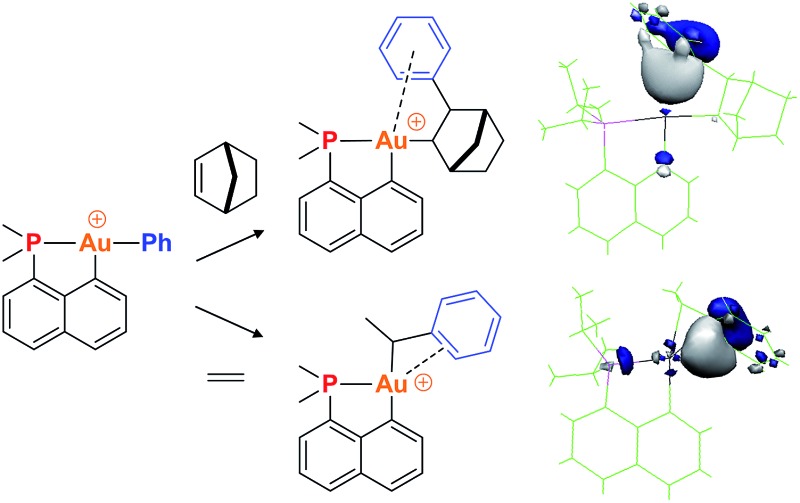
The synthesis and characterization of the first gold(iii)–arene complexes are described.

## Introduction

Transition metal π-complexes are key intermediates in many catalytic transformations involving alkenes, alkynes, allenes, and arenes^1^ as substrates. They represent an important class of organometallic compounds, with Zeise's salt [KPtCl_3_(H_2_C=CH_2_)] standing as the pioneering archetype.^2^ Nowadays, π-complexes are well-documented with almost all transition metals and their bonding situations being well understood (*cf.* the Dewar–Chatt–Duncanson model). However, this general picture does not apply to gold. Gold(i) π-complexes have been known for more than four decades and have recently witnessed an upsurge of interest owing to their relevance to the catalytic functionalisation of π-substrates.^3^ However, in stark contrast with their Pd(ii) and Pt(ii) congeners, gold(iii) π-complexes are extremely rare.^4^ To date, only a few gold(iii) alkene complexes have been authenticated,^5–8^ following the landmark work of Bochmann and Tilset in 2013.^5,6^ Moreover, despite the considerable development of gold(iii) chemistry over the last few years,^4,9–11^ other types of gold(iii) π-complexes are hitherto unknown.

In particular, no gold(iii)–arene complex has been characterized to date. This is all the more striking and unfortunate as gold(iii) complexes have the remarkable ability to activate aromatic C–H bonds, and π–arene complexes likely represent the initial stage of such processes.^12–14^ This view is supported by a recent computational study by Swang and co-workers.^15^ Accordingly, benzene was predicted to coordinate in an η^1^-fashion to a dicationic gold(iii) fragment [(bpy)Au^III^(C_6_H_5_)]^2+^, and low activation barriers were computed for benzene rotation as well as proton jumping between the coordinated phenyl and benzene ligands. However, attempts to synthesize relevant gold(iii) complexes were unsuccessful.^15^ While gold(iii)–arene complexes remain unprecedented, it must be noted that a few gold(i) complexes featuring intra and intermolecular Au(i)–arene interactions have been structurally characterized.^16–19^ In these complexes, the arene acts as a weakly coordinating ligand and it can be easily displaced by substrates such as alkynes.

**Chart 1:**
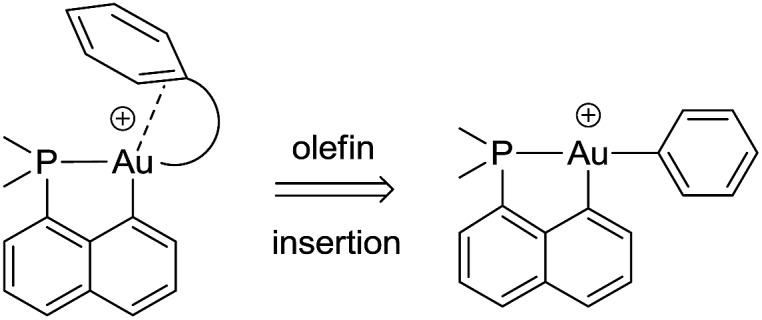
Schematic representation of the targeted gold(iii)–arene complexes and their generation by olefin insertion into a Au–aryl bond.

The key bottlenecks in the quest for gold(iii)–arene complexes lie in the design and access of low coordinated cationic gold(iii) complexes. We have recently developed a simple synthetic route to cyclometalated [(P,C)Au(iii)–Me]^+^ complexes and have shown their ability to readily insert olefins into the gold(iii)–carbon bond.^8,20^ The resulting low coordinated gold(iii) alkyl complexes showed a high tendency for β-hydride elimination^21^ and enabled the first authentication of a C–H agostic interaction with gold.^22^ These results motivated us to explore the synthesis of gold(iii)–arene complexes by olefin insertion into a gold(iii)–aryl bond (Chart 1).

As reported hereafter, this strategy proved fruitful and empowered us to characterize gold(iii)–arene complexes. Migratory insertion of norbornene and ethylene into the Au(iii)–Ph bonds is shown to be a very facile process that leads to gold(iii) alkyl complexes stabilized by intramolecular coordination of the phenyl ring. The mechanism of formation of the gold(iii)–arene complexes and their bonding situation have been thoroughly investigated spectroscopically, crystallographically, and computationally. The influence of arene coordination on the fate of low coordinated gold(iii) alkyl species is also discussed.

## Results and discussion

### Synthesis and characterization of the cationic (P,C) gold(iii)–phenyl complex **3**

**Scheme 1:**
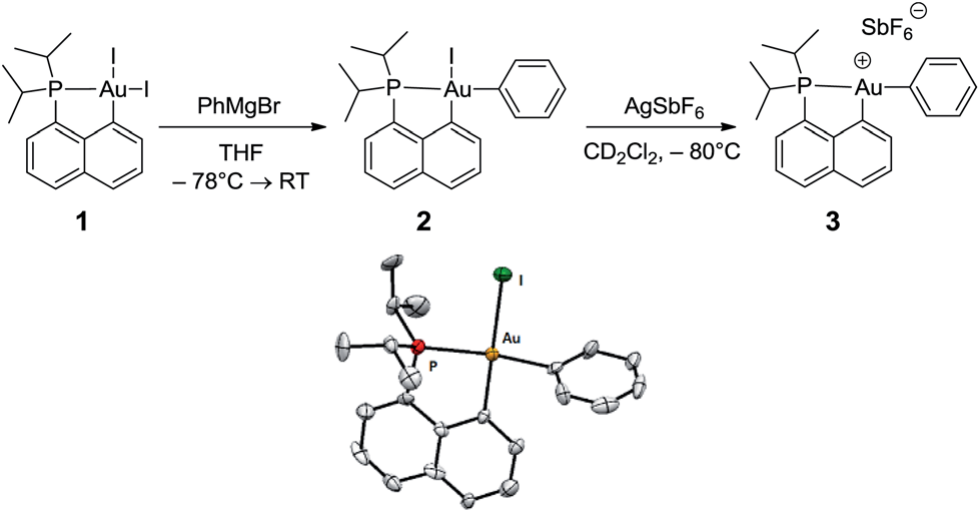
Preparation of the cationic gold(iii)–phenyl complex **3** and molecular view of its neutral precursor **2** with thermal ellipsoids drawn at the 50% probability level.

The cationic [(P,C)Au(iii)Ph]^+^ complex **3** was prepared in two steps (Scheme 1) from the [(P,C)AuI_2_] precursor **1** resulting from chelation-assisted oxidative addition to gold.^23^ First, treatment of **1** with PhMgBr (2.2 equiv.) enabled selective monoarylation, and complex **2** was isolated as a pale yellow powder (67% yield) after flash chromatography. Its molecular structure was ascertained by multinuclear NMR spectroscopy, high-resolution mass spectrometry (HRMS), elemental analysis, and X-ray diffraction.^24^

The iodide at the gold was then abstracted by adding complex **2** to AgSbF_6_ in dichloromethane. According to ^31^P NMR monitoring, the reaction was instantaneous at –80 °C and afforded a mixture of two cationic species in a 70/30 ratio (*δ* ^31^P: 77.4 and 80.8 ppm, respectively). The ^1^H and ^13^C NMR spectra also display two sets of similar signals, suggesting that the two species correspond to closely related forms of complex **3**.^25^ Variable-temperature ^31^P NMR studies indicated that the ratio between the two forms does not vary significantly from –80 to –20 °C (decomposition into several unidentified species occurs above –20 °C). However, the existence of a dynamic exchange between the two species was clearly apparent from the cross-peaks observed in the 2D ^31^P{^1^H} EXSY NMR spectrum. The ^13^C NMR signal for the phenyl *ipso* carbon atom C_*ipso*_ bound to gold is deshielded by about 10 ppm upon cationization (*δ* from 168.4 ppm for **2** to 177.2 ppm for the major form of **3**). The large ^2^ *J*_PC_ coupling constant is retained (134 Hz for **2**, 120 Hz for **3**), indicating that the phenyl ring still occupies the position *trans* to the phosphine moiety, in line with the dissymmetric *trans* influence of the (P,C) chelate. The reaction of complex **3** with olefins was then investigated by carrying out the iodide abstraction of **2** in the presence of norbornene or ethylene.

### Reaction of the cationic (P,C) gold(iii)–phenyl complex **3** with norbornene

First, a 1 : 1 mixture of complex **2** and norbornene (NB) was treated with AgSbF_6_ at –80 °C and the reaction mixture was warmed to room temperature. The ^31^P NMR spectrum showed the immediate formation of a single compound at *δ* 73.6 ppm. The ^1^H NMR spectrum revealed the complete consumption of NB (the corresponding olefin signal disappeared) and the presence of new aliphatic signals in line with the formation of the gold(iii) norbornyl complex **4**. Mono-insertion of NB into the Au–Ph bond of **3** was apparent from the integrals of the respective ^1^H NMR signals and was unambiguously confirmed by HRMS analysis. The cationic species **4** is too sensitive to be isolated in pure form, but NMR monitoring indicates that it is stable at room temperature (decomposition is observed after 48 h in solution at RT). The behavior of the gold(iii) phenyl complex **3** towards NB markedly differs from that we reported previously for the corresponding Au(iii) methyl derivative.^8,20^ In the latter case, double insertion of NB was observed, and the resulting product decomposed rapidly at low temperature despite its stabilization by γ-CH agostic interaction.^22^ To gain more insight into the stability of **4** and assess the possible π-coordination of the phenyl ring to gold, we assigned all the ^1^H and ^13^C NMR signals of **4** *via* 2D HSQC and HMBC experiments.^24^ It is important to note that NMR authentication of such π-interactions is *per se* challenging due to their weak and fluxional character. Accordingly, no diagnostic NMR data have been reported for the few characterized gold(i)–arene complexes. High fluxionality has also been noticed upon NMR characterization of Au(iii)–olefin.^5–8^ In the case of complex **4**, it is considered that the pendant phenyl ring is ideally positioned to occupy the vacant coordination site at the gold, with the norbornyl spacer inducing some rigidity (the gold atom and phenyl group are located on the *exo* face and are mutually *cis*). The ^1^H and ^13^C NMR spectra of **4** display broad resonance signals for the phenyl ring at room temperature, indicating some dynamic behavior. However, lowering the temperature to –20 °C resulted in well-resolved ^1^H and ^13^C NMR spectra.

**Fig. 1:**
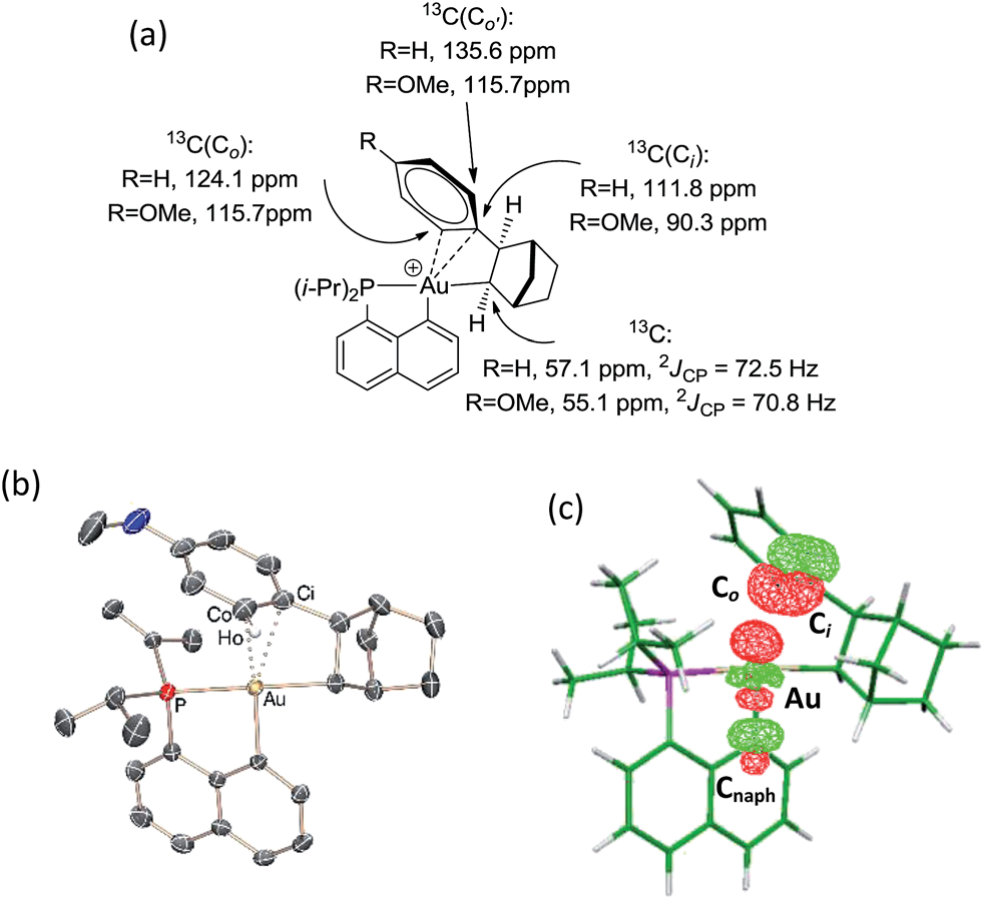
Complexes **4** and **4′**: (a) diagnostic NMR data determined experimentally at –20 °C, (b) molecular structure of **4′** in the solid state with ellipsoids set at 50% probability and hydrogen atoms expected on C_*ortho*_ omitted for clarity, (c) superposition of the donor π(C_*ipso*_C_*ortho*_) and acceptor σ*(AuC_naphthyl_) NBOs accounting for the Au(iii)–arene π-interaction in **4**.

The CH of the norbornyl moiety bound to gold resonates in ^13^C NMR as a doublet at 57.0 ppm with a large ^2^ *J*_PC_ coupling constant of 72.5 Hz, indicating a *trans* arrangement of the phosphorus atom and norbornyl group (Fig. 1a). In addition, six ^13^C NMR signals are found for the phenyl ring, suggesting hindered rotation around the C_sp^3^_–C_sp^2^_ bond and possibly some interaction between the phenyl ring and the gold center. The chemical shift of the *ipso* carbon is shifted to high field compared to that of 2-phenylbicycloheptane (PBH)^26^ (*δ* 111.8 ppm for **4** *vs.* 147.6 ppm for PBH) and appears as a doublet due to coupling with phosphorus (*J*_PC_ = 9.1 Hz). The presence of the gold(iii) center also affects the *ortho*-CH signals, one of them being shifted to high field by about 10 ppm (*δ* 124.1 and 135.6 ppm). These spectroscopic data are strongly reminiscent of those observed by Cheng and Catellani for related neutral Pd(ii)–arene complexes^27–29^ and suggest η^2^-coordination of the phenyl ring to gold(iii). Some of these Pd(ii)–arene complexes have been crystallographically characterized and their bonding situation has been thoroughly investigated.^27–29^

**Scheme 2:**
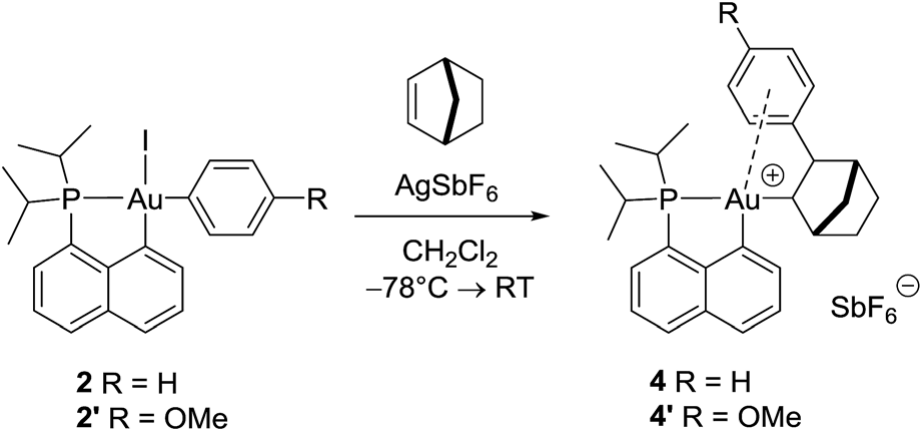
Formation of the gold(iii) norbornyl complexes **4** and **4′** by insertion of norbornene into the Au(iii)–Ar bond of **2** and **2′**.

With the aim of obtaining structural confirmation of the gold(iii)–arene interaction, we prepared another gold(iii)–aryl complex **2′** with an OMe group in the *para* position of the phenyl ring (Scheme 2). This electron-rich arene ring was envisioned to impart greater stability to the gold(iii) π-complex and hopefully higher crystallinity. Starting from **2′**, the insertion of norbornene also works well. The ensuing gold(iii)–arene complex **4′** was isolated and fully characterized. To our delight, crystals suitable for X-ray diffraction analysis were obtained in this case. Accordingly, complex **4′** (Fig. 1b) adopts a 3-coordinated T-shaped structure (PAuC_NB_ and PAuC_naphthyl_ bond angles of 177.29(7)° and 84.15(7)°, respectively). As expected, the insertion of norbornene into the Au–Ph bond places the gold atom and the phenyl ring *cis* and *exo* to the norbornyl group. The arene sits *trans* to the naphthyl backbone with short contacts between gold and two carbon atoms (C_*ipso*_ and one C_*ortho*_). The Au–C_*ipso*_ distance (2.416(2) Å) is shorter than the Au–C_*ortho*_ distance (2.593(3) Å), indicating dissymmetric η^2^-coordination of the arene to gold(iii). The interaction of the (*p*-OMe)-phenyl ring with the gold(iii) center is also apparent from NMR (Fig. 1a). As for **4**, the ^13^C signals for C_*ipso*_ (*δ* 90.3 ppm) and one of the C_*ortho*_ atoms (*δ* 115.7 ppm) of **4′** are shifted to high field compared to those of 2-(*p*-OMe)-phenylbicycloheptane^26^ (Δ*δ* ^13^C = 49 ppm for C_*ipso*_ and 13 ppm for C_*ortho*_).^30^ Interestingly, the NMR signals for the (*p*-OMe)-phenyl ring are desymmetrized even at room temperature, suggesting tighter arene coordination in **4′** *vs.* **4**.

### DFT study of complex **4**: structure and mechanism of formation

DFT calculations were performed at the B3PW91/SDD+f(Au),6-31G**(other atoms) level of theory. Two isomeric forms of complex **4** were located on the potential energy surface, corresponding to *cis* and *trans* arrangements of the two carbon atoms, C_norbornyl_ and C_naphthyl_, at the gold (*cis*-**4** and *trans*-**4**, respectively). In line with experimental observations, complex *cis*-**4** was calculated to be thermodynamically more stable than *trans*-**4** by 13.2 kcal mol^–1^.^24^ To confirm the experimental assignments, the ^13^C NMR data for complex *cis*-**4** were computed using the GIAO method and the IGLO-II basis set (P, C, and H atoms).^24^ The coupling constant between the phosphorus and C_norbornyl_ atoms (^2^ *J*_PC_ = 75.9 Hz) nicely matches that observed experimentally (72.5 Hz). Moreover, the C_*ipso*_ and one of the C_*ortho*_ atoms are significantly shielded compared to the other signals (Table S1†), in line with η^2^-coordination to gold.^24^ The optimized geometry of *cis*-**4** (Fig. S26†) very much resembles that determined crystallographically for **4′**. The gold center is 3-coordinated and adopts T-shaped geometry (PAuC_NB_ and PAuC_naphthyl_ bond angles of 171.7° and 82.9°, respectively). The arene occupies the fourth coordination site of the gold(iii) center and is η^2^-coordinated. Short contacts are observed with the C_*ipso*_ and one of the C_*ortho*_ atoms (2.62 and 2.63 Å, respectively). The coordination of the aryl ring to gold was further confirmed by Natural Bond Orbital (NBO) analysis. At the second-order perturbation level, donation from the π(C_*ipso*_C_*ortho*_) orbital to the σ*(AuC_naphthyl_) orbital was found with a delocalization energy Δ*E*_(2)_ of 30.7 kcal mol^–1^ (Fig. 1c). This indicates the presence of a rather strong donor–acceptor interaction compared with those encountered in related [(P,C)Au(iii)R]^+^/norbornene adducts [Δ*E*_(2)_ of 9–18 kcal mol^–1^].^8,24^ Similar results were obtained for the *p*-OMe substituted complex **4′**. Its optimized structure is consistent with that determined crystallographically, with a slightly more dissymmetric η^2^-coordination of the arene (C_*ipso*_Au and C_*ortho*_Au distances of 2.44 and 2.80 Å, respectively). In NBO, the delocalization energy Δ*E*_(2)_ for the π(C_*ipso*_C_*ortho*_) → σ*(AuC_naphthyl_) interaction (36.6 kcal mol^–1^) is slightly higher than for **4**, in line with a strengthening of the π interaction.^24^

**Fig. 2:**
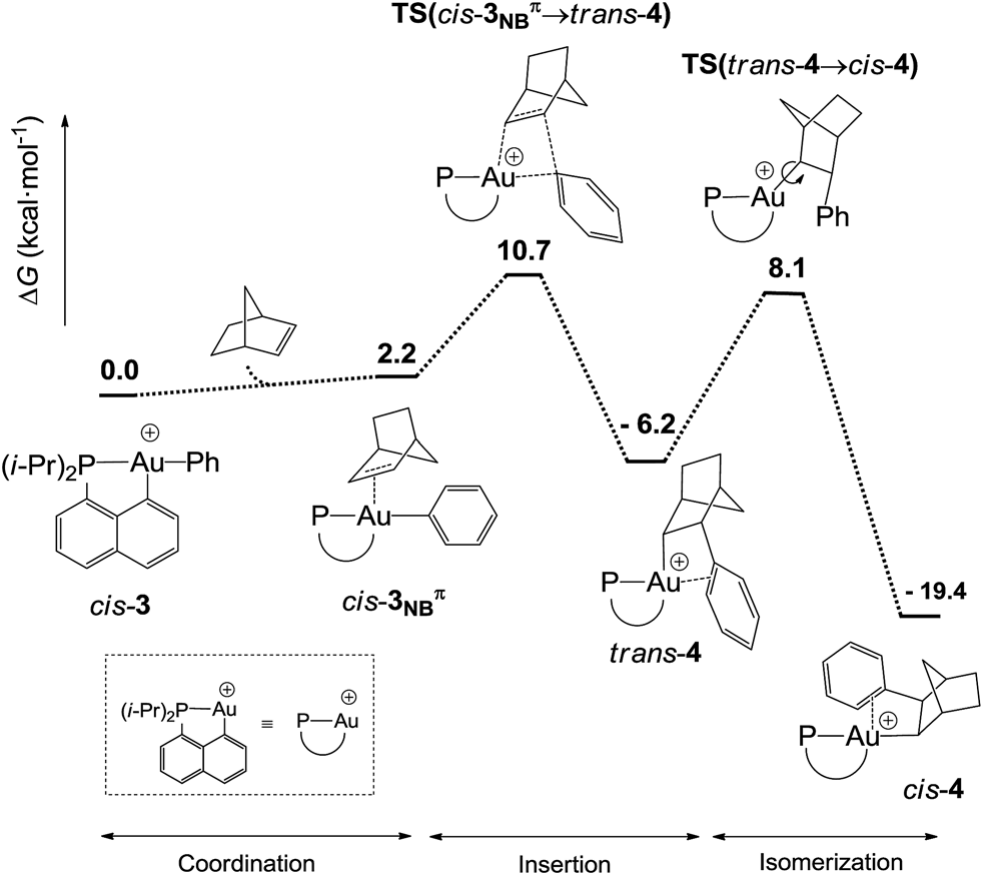
Reaction profile (Δ*G* in kcal mol^–1^) computed at the B3PW91/SDD+f(Au)/6-31G**(other atoms) level of theory for the formation of the gold(iii)–arene complex **4** upon reaction of *cis*-**3** with NB.

The reaction profile accounting for the formation of complex **4** from **3** was also computed. This involves the coordination and migratory insertion of norbornene into the Au–Ph bond, followed by *cis*/*trans* isomerization (Fig. 2). The insertion of NB proceeds *via* an in-plane 4-center transition state and gives the gold(iii)–norbornyl complex *trans*-**4**. The associated activation barrier is low (Δ*G* ^≠^ = 10.7 kcal mol^–1^), much lower than that computed for the reaction of NB with the corresponding Au(iii)–Me complex (Δ*G* ^≠^ = 18.7 kcal mol^–1^).^31^ Complex *trans*-**4** is not observed experimentally. It spontaneously isomerizes (with a low barrier Δ*G* ^≠^ of 14.3 kcal mol^–1^) into the more thermodynamically stable complex *cis*-**4**. The Gibbs free energy Δ*G* for the overall transformation *cis*-**3** → *cis*-**4** is –19.4 kcal mol^–1^.

### Formation of the neutral cyclometalated complex **5**: base-promoted C–H activation of **4**

**Scheme 3:**
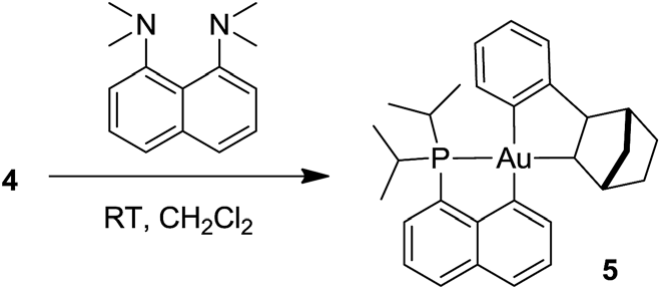
Cyclometallation of the Au(iii)–arene complex **4** leading to **5**.

Given the propensity of electrophilic Au(iii) complexes to activate aromatic C–H bonds, we were intrigued by the possibility of promoting the auration of the π-coordinated arene ring of **4**. Heating a DCM solution at 45 °C led only to decomposition products, but in the presence of a base such as pyridine or a proton sponge, C–H activation proceeded rapidly (Scheme 3). The resulting cyclometalated complex **5** was isolated in 95% yield and fully characterized.^24^

The insertion of NB into the Au–Ph bond of **3** is a very facile process. It affords a straightforward entry to the cationic gold(iii) norbornyl complex **4** and to the neutral cyclometalated complex **5** following C–H activation. The η^2^-coordination of the phenyl ring to gold has been unambiguously authenticated in **4**. This intramolecular gold(iii)–arene interaction provides significant thermal stability to complex **4**. Indeed, cationic gold(iii) alkyl complexes tend to rapidly undergo reductive elimination or β-hydride elimination even at low temperature and only a few such species have been characterized so far.^21,22^ These observations prompted us to explore the reactivity of the cationic phenyl complex **3** towards ethylene and to thereby probe the impact of π–arene interactions on the reactivity of gold(iii) alkyl species.

### Reaction of the cationic (P,C)gold(iii)–phenyl complex **3** with ethylene

**Scheme 4:**
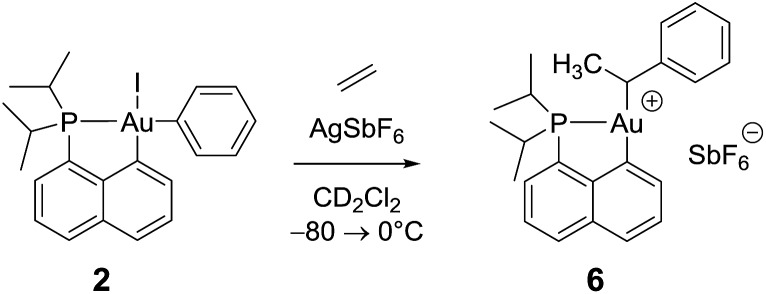
Formation of the gold(iii) alkyl complex **6** upon reaction of the cationic (P,C)gold(iii) phenyl complex with ethylene.

Remarkably, complex **3** reacts swiftly with ethylene (1 bar) at low temperature whereas insertion of ethylene required 4 hours at 50 °C and 2 bars for the related gold(iii) methyl complex.^21^ Moreover, the reaction of the [(P,C)AuMe]^+^ species with ethylene directly afforded higher olefins (propylene and butenes) and a cationic 2-coordinated bis(phosphine) gold(i) complex (*δ* ^31^P 60 ppm), while complex **3** is instantaneously converted into a single gold(iii) species **6**, as indicated by ^31^P NMR spectroscopy (*δ* ^31^P 95.9 ppm). The new compound **6** was unequivocally characterized by multinuclear NMR spectroscopy. In particular, 2D ^1^H–^13^C NMR data clearly indicated the presence of the *sec*-alkyl group –CH(CH_3_)Ph at the gold (Scheme 4).

**Fig. 3:**
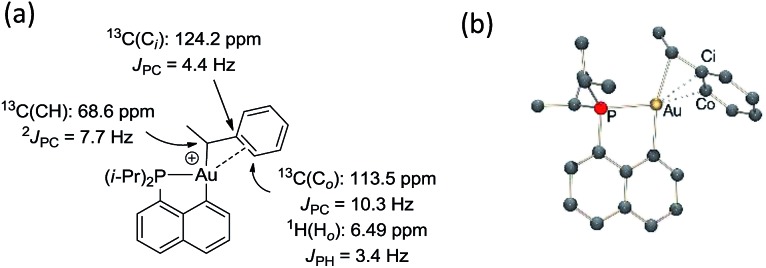
Complex **6**: (a) diagnostic NMR data determined experimentally at 0 °C and (b) DFT-optimized structure of *trans*-**6** calculated at the B3PW91/SDD+f(Au)/6-31G**(other atoms) level of theory: Au–C_*ipso*_ 2.28 Å and Au–C_*ortho*_ 2.55 Å.

The methine group at the gold resonates in ^13^C NMR at *δ* 68.6 ppm with a small ^2^ *J*_PC_ coupling constant of 7.7 Hz, indicating that the *sec*-alkyl moiety sits in this case in a *trans* position to the C_naphthyl_ atom. The ^13^C NMR spectrum also revealed a significant shift to high field of the C_*ipso*_ and one of the C_*ortho*_ carbon atoms of the phenyl ring compared to those of ethylbenzene (Δ*δ* ^13^C = 20 ppm for C_*ipso*_ and 16 ppm for C_*ortho*_, Fig. 3a).^32^ All the carbon atoms of the phenyl ring except for one of the C_*meta*_ couple with phosphorus (4.4 < *J*_PC_ < 10.3 Hz), and one of the H_*ortho*_ resonance signal also display coupling to phosphorus (*J*_PH_ = 3.4 Hz). All these data support η^2^-coordination of the phenyl ring to gold(iii), as in the case of the gold(iii) norbornyl complex **4**.

Complex **6** shows unusual thermal stability (no degradation is observed after 24 h at 0 °C) given the presence of hydrogen atoms in a β position and the proclivity of cationic 3-coordinated gold(iii) alkyl complexes to undergo β-H elimination.^21^ Indeed, the corresponding *n*-propyl and *n*-butyl complexes were previously found to rapidly release propene and butenes at 0 °C. A similar process with the formation of styrene is observed with **6** but only after 1 h at room temperature. The higher stability of complex **6** is most likely due to the π-interaction of the phenyl ring with the gold(iii) center.

The structure of complex **6** was further assessed by DFT and NBO calculations. Accordingly, complex **6** adopts a slightly distorted T-shaped geometry (Fig. 3b and Table S3†) with PAuC(H) and C_naphthyl_AuC(H) bond angles of 109.4 and 166.3°, respectively. The pendant phenyl ring is significantly bent towards the cationic [(P,C)Au]^+^ fragment [AuC(H)C_*ipso*_ bond angle = 74.2°] and interacts with the gold center *via* the C_*ipso*_ and C_*ortho*_ carbons in an asymmetrical manner (Au–C_*ipso*_: 2.28 Å, Au–C_*ortho*_: 2.55 Å). The η^2^-coordination of the phenyl ring to gold(iii) is corroborated by the ^13^C NMR data computed for the corresponding C_*ipso*_ and C_*ortho*_ carbon atoms (Table S3†). The respective resonance signals are shifted to high field (*δ* 124.6 and 120.6 ppm for C_*ipso*_ and C_*ortho*_, respectively) and display *J*_PC_ coupling constants of 5 and 10.7 Hz, respectively. NBO analysis (Fig. S31†) identified a donation from the π(C_*ipso*_C_*ortho*_) orbital to the Au σ*(AuC_naphthyl_) orbital with a delocalization energy Δ*E*_(2)_ of 38.6 kcal mol^–1^, a value very close to that found in the gold(iii) norbornyl complex **4**. It is interesting to note that complex **6** is characterized experimentally in its *trans* form (the C_naphthyl_ and C_alkyl_ atoms are in a *trans* arrangement) and does not isomerize into the more thermodynamically stable *cis*-isomer (Δ*G* = –5.2 kcal mol^–1^ and Δ*G* ^≠^ = 25 kcal mol^–1^, Fig. S30†).

**Fig. 4:**
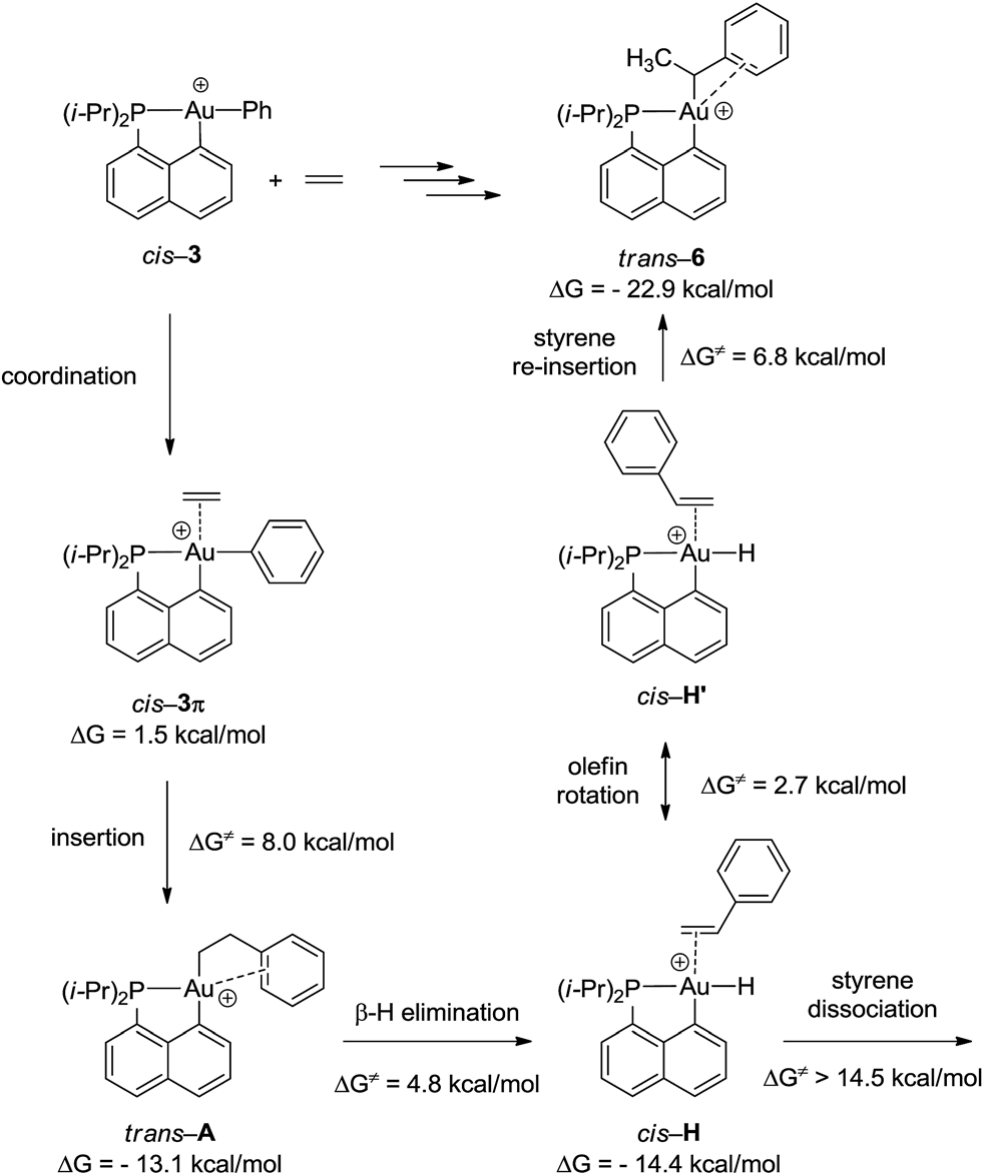
Reaction sequence computed at the B3PW91/SDD+f(Au)/6-31G**(other atoms) level of theory for the formation of the gold(iii)–arene complex **6** (Δ*G* values set with the initial reactants *cis*-**3** + ethylene as origin, while Δ*G* ^≠^ values correspond to the activation barriers of each step).

To gain a deeper insight into the mechanism of formation of complex **6**, the reaction of the cationic complex **3** with ethylene was thoroughly investigated by DFT calculations (Fig. 4 and S32†). Ethylene first coordinates to gold to give complex *cis*-**3π** (Δ*G* = 1.5 kcal mol^–1^) and then inserts into the Au–Ph bond to afford the Au(CH_2_CH_2_Ph) complex *trans*-**A**. The activation barrier for the migratory insertion is low (Δ*G* ^≠^ = 9.5 kcal mol^–1^ from the initial reactants). In line with experimental observations, insertion into the Au–Ph bond is kinetically more facile than into the Au–Me bond (the corresponding activation barrier reaches 16.5 kcal mol^–1^). Complex *trans*-**A** displays some π–arene interaction [according to NBO, Δ*E*_(2)_ = 10.5 kcal mol^–1^] (Fig. S33†) but coordination of the pendant phenyl ring to gold(iii) is less stabilizing with the flexible CH_2_CH_2_ spacer than that encountered in complex **6**. In line with the fact that *trans*-**A** is not observed experimentally, it easily undergoes β-hydride elimination (Δ*G* ^≠^ = 4.8 kcal mol^–1^).^33^ The reaction is slightly exergonic and leads to intermediate *cis*-**H**. Finally, the Au(iii)–H styrene complex readily undergoes (2,1) re-insertion of styrene into the Au–H bond to give complex *trans*-**6** with an activation barrier of Δ*G* ^≠^ = 8.0 kcal mol^–1^ from *cis*-**H** to *trans*-**6** (styrene dissociation is comparatively disfavored with Δ*G* ^≠^ = 14.5 kcal mol^–1^).^34,35^ As mentioned above, η^2^-coordination of the phenyl ring substantially stabilizes complex *trans*-**6** (Δ*G* = –22.9 kcal mol^–1^), and the latter species is actually the thermodynamic product of ethylene insertion into the Au–Ph bond, enabling its experimental characterization. Note that in accordance with experimental observations, the arene coordination disfavors β-hydride elimination from complex *trans*-**6** (Fig. S34†).^24^ The associated energy barrier Δ*G* ^≠^ amounts to 16.5 kcal mol^–1^ *vs.* 7.1 kcal mol^–1^ for the corresponding [(P,C)Au(iii)(*n*-Bu)]^+^ complex.^21^ The arene coordination also disfavors isomerization of *trans*-**6** into its more thermodynamically stable *cis* isomer (Fig. S30†). The latter process requires a high activation energy (Δ*G* ^≠^ = 25 kcal mol^–1^) and is even less favored than β-hydride elimination.

## Conclusions

A suitable synthetic strategy based on olefin insertion into a Au(iii)–Ph bond allowed us to experimentally characterize gold(iii)–arene complexes for the first time. The η^2^-coordination of the remote phenyl ring to gold has been substantiated spectroscopically, crystallographically, and computationally. The isolation and characterization of such intermediates provides interesting information on the interaction between arenes and gold(iii) species in the context of gold-catalyzed functionalization of aromatic C–H bonds.^12,14^

This study also shows that migratory insertion of olefins such as norbornene and ethylene into the Au(iii)–Ph bond is a very facile process. This transformation and the ensuing intermediates, which are stabilized intramolecularly by π–arene coordination, are relevant to a number of key catalytic processes (such as Mizoroki–Heck coupling, the Catellani reaction, and the hydroarylation of olefins).

Future work from our group will seek to take advantage of the increasing scope of gold(iii) species and reactivities to develop valuable catalytic transformations.
