# Supplementary material for: Gold(iii)–arene complexes by insertion of olefins into gold–aryl bonds
Source: Chem Sci. 2017 Apr 19;8(6):4539–45. doi: 10.1039/c7sc00145b (PMC5472032; doi:10.1039/c7sc00145b)
Supplement: Supplementary file 1 [file SC-008-C7SC00145B-s001.pdf]

Electronic Supplementary Material (ESI) for Chemical Science.
This journal is © The Royal Society of Chemistry 2017

Supporting Information

Materials and methods S2

Experimental procedures and analytical data S3

NMR spectra S11

Crystallographic data S27

Computational data S28

Materials and methods

All reactions and manipulations were carried out under an atmosphere of dry argon using standard Schlenk techniques or in a glovebox under inert atmosphere. Dry, oxygen-free solvents were employed. Melting points were determined with a Stuart SMP40 apparatus and are uncorrected. Solution ^{1}H, ^{13}C, ^{11}B, ^{19}F and ^{31}P NMR spectra were recorded on Bruker Avance 300, 400 or 500 spectrometers at 298 K. Chemical shifts (δ) are expressed with a positive sign, in parts per million. ^{1}H and ^{13}C chemical shifts reported are referenced internally to residual protio- (^{1}H) or deutero- (^{13}C) solvent, while ^{31}P chemical shifts are relative to 85% H_3PO_4. The following abbreviations and their combinations are used: br, broad; s, singlet; d, doublet; t, triplet; q, quartet, m, multiplet. The ^{1}H and ^{13}C resonance signals were attributed by means of 2D HSQC and HMBC experiments. Mass spectra were recorded on a Waters LCT apparatus. Elemental analyses were performed by the in-house service at the *Laboratoire de Chimie de Coordination* (205, Route de Narbonne, 31077, Toulouse, France) on a PerkinElmer 2400 Series II system. GC/MS was performed on a Perkin Elmer Clarus 500 machin equipped with a head space injector with a BPX-volatiles column. All starting materials were purchased from Aldrich and used as received. Complex **1** was prepared following previously described procedure[1].

[1] Rekhroukh, F.; Brousses, R.; Amgoune, A.; Bourissou, D. *Angew. Chem. Int. Ed.* **2015**, *54*, 1266.

Experimental procedures and analytical data

Synthesis of Gold(III) aryl complex 2

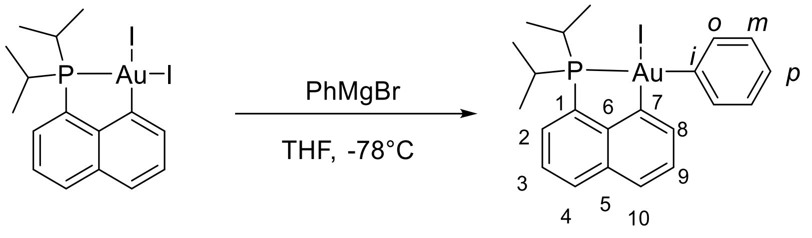

1 **2**

A solution of PhMgBr (2.2 equiv, 1M in THF) was added dropwise to a solution of complex **1** (200 mg, 0.27 mmol) in THF (15 mL) at –78°C. The reaction mixture was stirred for 1h at –78°C and then at room temperature for 1h. While warming to room temperature, the reaction mixture cleared up to light yellow solution. The solvent was then removed in vacuo and the resulting residue was dissolved in DCM (8 mL) and washed with distilled water (1 mL). The organic phase was dried over Na_2SO_4 and filtered through celite. All volatiles were then removed in vacuo to yield the product as a yellow pale solid. Complex **2** was purified by flash column chromatography (silica, eluent: Pentane/DCM 80:20) to yield **2** as a yellow pale powder. Yield: 125 mg (67 %).

MP: 142 °C**.** **^{1}H NMR** (500 MHz, CD_2Cl_2): δ 8.03 (dd, $^3J_{HH}$ = 7.0 Hz, $^5J_{PH}$ = 1.3 Hz, 1H, H_8), 7.84 (ddd, $^3J_{HH}$ = 7.0 Hz; $^3J_{HH}$ = 7.2 Hz; $^5J_{PH}$ = 7.1 Hz, 1H, H_3), 7.77 (d, $^3J_{HH}$ = 7.8 Hz, 1H, H_{10}), 7.61 (ddd, $^4J_{HH}$ = 7.1 Hz; $^3J_{HH}$ = 7.2 Hz; $^3J_{PH}$ = 2.1 Hz, 1H, H_2), 7.52 (ddd, $^3J_{HH}$ = 7.8 Hz; $^3J_{HH}$ = 7.7 Hz; $^5J_{PH}$ = 8.0 Hz, 2H, H_{meta}), 7.30 (m, 2H, H_{ortho}), 7.27 (m, 2H, H_9), 7.12 (ddd, $^3J_{HH}$ = 7.7 Hz; $^4J_{HH}$ = 1.2 Hz; $^6J_{PH}$ = 1.1 Hz,1H, H_{para}), 7.06 (d, $^3J_{HH}$ = 7.4 Hz, 1H, H_4), 3.23 (heptd, $^3J_{HH}$ = 7.0 Hz; $^2J_{PH}$ = 11.4 Hz, 2H, CH*iPr*), 1.42 (dd, $^3J_{HH}$ = 7.0 Hz, $^3J_{PH}$ = 18.0 Hz, 6H, CH_3*iPr*), 1.30 (dd, $^3J_{HH}$ = 7.0 Hz, $^3J_{PH}$ = 16.1 Hz, 6H, CH_3*iPr*); **^{31}P{^{1}H} NMR** (202 MHz, CD_2Cl_2): δ 67.9 (s); **^{13}C{^{1}H} NMR** (125 MHz, CD_2Cl_2): δ 168.4 (d, $^2J_{PC}$ = 133.7 Hz, C_{ipso}), 152.2 (d, $^3J_{PC}$ = 1.3 Hz, C_7), 149.1 (d, $^3J_{PC}$ = 30.7 Hz, C_6), 135.1 (d, $^3J_{PC}$ = 14.1 Hz, C_5), 134.4 (d, $^4J_{PC}$ = 2.3 Hz, C_{meta}), 134.1 (s, C_8), 133.2 (d, $^5J_{PC}$ = 2.4 Hz, C_4), 131.7 (s, C_3), 129.6 (d, $^3J_{PC}$ = 9.2 Hz, C_{ortho}), 129.4 (d, $^1J_{PC}$ = 47.0 Hz, C_1), 127.4 (s, C_9), 126.9 (d, $^4J_{PC}$ = 1.0 Hz, C_{10}), 125.2 (d, $^5J_{PC}$ = 1.5 Hz, C_{para}), 124.6 (d, $^2J_{PC}$ = 7.6 Hz, C_2), 27.1 (d, $^1J_{PC}$ = 25.8 Hz, CH*iPr*), 19.2 (d, $^2J_{PC}$ = 2.5 Hz, CH_3*iPr*), 18.5 (d, $^2J_{PC}$ = 1.5 Hz, CH_3*iPr*); **HRMS (DCI-CH_4+)**: calcd for $[M]^+$ = $C_{22}H_{25}AuP^+$: 517.1359. Found: 517.1382; **Elemental Analysis**: calcd for $C_{22}H_{16}AuIP$: C 41.01, H 3.91. Found: C 40.71, H 3.68.

Synthesis of Gold(III) aryl complex 2'

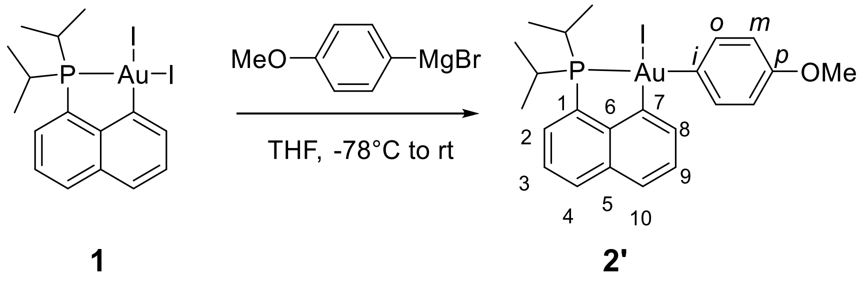

A 0.5M solution of 4-methoxyphenylmagnesium bromide in THF (0.69 mL, 0.35 mmol) was added dropwise to a solution of complex **1** (200 mg, 0.29 mmol) in THF (15 mL) at -78°C. The reaction mixture was allowed to slowly warm back to room temperature (solution cleared up to light yellow) and stirred for 1h. The solvent was then removed in vacuo and the resulting residue was dissolved in DCM (8 mL) before filtrating over celite pad. All volatiles were then removed and the crude mixture was purified by flash column chromatography (silica, eluent: Pentane/DCM 100:0 to 70:30) to afford compound **2'** as a white powder in 82% yield (159 mg).

MP: 175°C (decomposition); **^{1}H NMR** (500 MHz, CD_2Cl_2): δ 8.02 (ddd, $^3J_{HH}$ = 8.1 Hz, $^4J_{HH}$ = 1.0, $^5J_{PH}$ = 2.4 Hz, 1H, H_8), 7.83 (ddd, $^3J_{HH}$ = 7.1, $^3J_{HH}$ = 1.1, $^4J_{PH}$ = 7.1 Hz, 1H, H_3), 7.77 (d, $^3J_{HH}$ = 8.1, 1H, H_{10}), 7.60 (ddd, $^3J_{HH}$ = 8.0, $^4J_{HH}$ = 7.1, $^3J_{PH}$ = 2.1 Hz, 1H, H_2), 7.45-7.41 (m, 2H, H_{meta}), 7.28 (t, $^3J_{HH}$ = 7.7 Hz, H_9), 7.08 (dd, $^3J_{HH}$ = 7.4 Hz, $^4J_{HH}$ = 1.1 Hz, 1H, H_4), 6.93-6.89 (m, 2H, H_{ortho}), 3.83 (s, 3H, OMe), 3.22 (heptd, $^3J_{HH}$ = 7.1 Hz; $^2J_{PH}$ = 11.2 Hz, 2H, CH*i*Pr), 1.41 (dd, $^3J_{HH}$ = 7.1 Hz, $^3J_{PH}$ = 18.1 Hz, 6H, CH_3*i*Pr), 1.29 (dd, $^3J_{HH}$ = 7.1 Hz, $^3J_{PH}$ = 16.1 Hz, 6H, CH_3*i*Pr); **^{31}P{^{1}H} NMR** (202 MHz, CD_2Cl_2): δ 68.7 (s); **^{13}C{^{1}H} NMR** (125 MHz, CD_2Cl_2): δ 158.8 (d, $^2J_{PC}$ = 136.4 Hz, C_{ipso}), 157.4 (d, $^5J_{PC}$ = 1.7 Hz, C_{para}), 152.2 (s, C_7), 148.9 (d, $^2J_{PC}$ = 30.7 Hz, C_6), 134.9 (d, $^3J_{PC}$ = 14.2 Hz, C_5), 134.5 (d, $^4J_{PC}$ = 3.4 Hz, C_{meta}), 133.9 (s, C_8), 133.1 (d, $^4J_{PC}$ = 2.4 Hz, C_4), 131.5 (s, C_3), 129.7 (d, $^1J_{PC}$ = 46.5 Hz, C_1), 127.4 (s, C_{10}), 127.3 (s, C_9), 126.2 (d, $^2J_{PC}$ = 7.7 Hz, C_2), 114.9 (d, $^3J_{PC}$ = 10.0 Hz, C_{ortho}), 55.4 (s, OMe), 26.9 (d, $^1J_{PC}$ = 25.9 Hz, CH*i*Pr), 19.0 (d, $^2J_{PC}$ = 2.4 Hz, CH_3*i*Pr), 18.4 (d, $^2J_{PC}$ = 1.2 Hz, CH_3*i*Pr); **HRMS (ESI+)**: calcd for $[M]^+$ = $C_{23}H_{27}AuOP^+$: 547.1465. Found: 547.1479; **Elemental Analysis**: calcd for $C_{23}H_{27}AuIOP$: C 40.97, H 4.04. Found: C 41.01, H 3.88.

Generation of cationic Gold(III) aryl complex 3

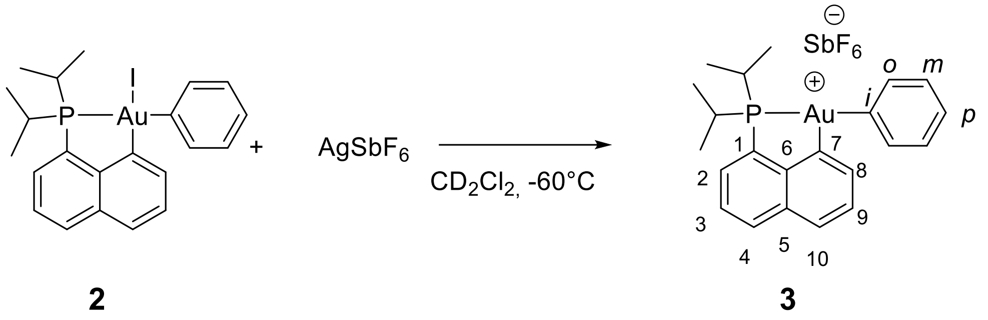

In a glovebox, a screw-cap NMR tube was charged with $AgSbF_6$ (8.7 mg, 0.025 mmol) in dichloromethane-d^2 (0.35 mL). Complex **2** (16.3 mg, 0.025 mmol) was transferred into a small glass vial and solubilized in dichloromethane-d^2 (0.35 mL). The prepared solution was loaded into a plastic syringe equipped with stainless steel needle. The syringe was closed by blocking the needle with a septum. Outside of the glovebox, the NMR tube was put under positive argon pressure and cooled down to – 80 °C (Acetone/N_2 coldbath). At this temperature, the solution of complex **2** was added. The tube was kept at – 60 °C and immediately introduced into the NMR machine for analysis. A mixture of two cationic species in a 70/30 ratio was observed by ^{31}P NMR spectroscopy at – 60°C (δ ^{31}P: 77.4 ppm for the major species and 80.8 ppm for the minor species), corresponding to two forms of complex **3**. Complete NMR characterization of the major species is described hereafter.

^{1}H NMR (500 MHz, CD_2Cl_2, 213K): δ 8.03 (m, 1H, H_2), 7.89 (m, 1H, H_4), 7.87 (m, 1H, H_{10}), 7.77 (m, 2H, H_{meta}), 7.75 (m, 2H, H_{ortho}), 7.71 (m, 1H, H_3), 7.47 (m, 1H, H_{para}), 7.25 (t, $^3J_{HH}$ = 7.5 Hz, 1H, H_9), 6.47 (d, $^3J_{HH}$ = 7.5 Hz, 1H, H_8), 3.14 (m, 2H, CH*iPr*), 1.34 (m, 6H, CH_3*iPr*), 1.25 (m, 6H, CH_3*iPr*); **^{31}P{^{1}H} NMR** (202 MHz, CD_2Cl_2, 213K): δ 77.4 (bs); **^{13}C{^{1}H} NMR** (125 MHz, CD_2Cl_2, 213K): δ 177.2 (d, $^2J_{PC}$ = 120.2 Hz, C_{ipso}), 149.6 (s, C_7), 147.1 (d, $^3J_{PC}$ = 25.5 Hz, C_6), 134.7 (d, $^3J_{PC}$ = 13.8 Hz, C_5), 133.5 (s, C_2), 132.5 (s, C_4), 132.4 (s, C_8), 131.7 (s, C_{ortho}), 129.4 (bs, C_{meta}), 128.4 (s, C_{10}), 127.5 (bs, C_{para}), 126.7 (d, $^3J_{PC}$ = 7.5 Hz, C_3), 126.3 (s, C_9), 124.7 (d, $^1J_{PC}$ = 53.0 Hz, C_1), 26.8 (d, $^1J_{PC}$ = 26.6 Hz, CH*iPr*), 18.5 (s, CH_3*iPr*), 17.8 (s, CH_3*iPr*).

Synthesis of Gold(III)–arene complex 4

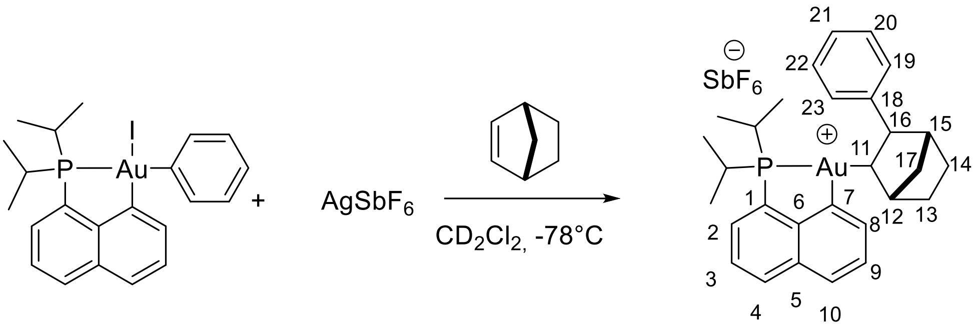

A solution of $AgSbF_6$ (9.6 mg, 0.028 mmol) in 0.2 mL of CD_2Cl_2 was added at – 80°C to a mixture of complex **2** (15.0 mg, 0.023 mmol) and NB (0.028 mmol) in CD_2Cl_2 (0.4 mL). The reaction was allowed to warm up to room temperature and kept for 10 min at this temperature (the reaction medium turns from colorless to yellow). Complex **4** was characterized at – 20°C without purification.

^{1}H NMR (400 MHz, CD_2Cl_2, 253K): δ 8.19 (d, $^{3}J_{HH}$ = 7.0 Hz, 1H, H_{23}), 8.07 (d, $^{3}J_{HH}$ = 8.0 Hz, 1H, H_4), 8.00-7.92 (m, 3H, H_{19}&H_{22}&H_{20}), 7.89 (d, $^{3}J_{HH}$ = 8.0 Hz, 1H, H_{10}), 7.85 (d, $^{3}J_{HH}$ = 7.8 Hz, H_8), 7.69-7.74 (m, 2H, H_2&H_{21}), 7.62 (m, 1H, H_3), 7.55 (t, $^{3}J_{HH}$ = 7.8 Hz, H_9), 4.77 (dd, $^{3}J_{HH}$ = 8.7 Hz, $^{4}J_{PH}$ = 11.3 Hz, 1H, H_{16}), 3.00 (bs, 1H, H_{12}), 2.90 (bs, 1H, H_{15}), 2.55 (heptd, $^{3}J_{HH}$ = 7.1 Hz; $^{2}J_{PH}$ = 11.3 Hz, 1H, CH*i*Pr), 2.44 (m, 1H, H_{11}), 2.20-2.28 (m, 1H, CH*i*Pr), 2.01-1.91 (m, 2H, H_{13}&H_{17}), 1.85-1.76 (m, 1H, H_{14}), 1.57 (m, 1H, $H_{17'}$), 1.45-1.39 (m, 3H, $H_{13'}$&$H_{14'}$), 1.12-1.04 (m, 6H, CH_3*i*Pr), 0.97-0.86 (m, 6H, CH_3*i*Pr); **^{31}P{^{1}H} NMR** (162 MHz, CD_2Cl_2, 253K): δ 73.6 (s); **^{13}C{^{1}H} NMR** (100 MHz, CD_2Cl_2, 253K): δ 146.1 (d, $^{3}J_{PC}$ = 3.6 Hz, C_7), 144.9 (d, $^{2}J_{PC}$ = 27.8 Hz, C_6), 135.5 (s, C_{19}), 134.6 (d, $^{3}J_{PC}$ = 11.2 Hz, C_5), 134.3 (s, C_{20}), 133.1 (s, C_{21}), 133.0 (d, $^{4}J_{PC}$ = 2.4 Hz, C_4),132.8 (s, C_{22}), 132.5 (s, C_2), 129.6 (d, $^{4}J_{PC}$ = 1.3 Hz, C_{10}), 128.9 (d, $^{4}J_{PC}$ = 1.0 Hz, C_8), 127.5 (d, $^{5}J_{PC}$ = 0.8 Hz, C_9), 126.6 (d, $^{3}J_{PC}$ = 7.5 Hz, C_3), 124.0 (s, C_{23}), 122.5 (d, $^{3}J_{PC}$ = 48.3 Hz, C_1), 111.7 (d, $^{4}J_{PC}$ = 9.6 Hz, C_{18}), 57.0 (d, $^{2}J_{PC}$ = 72.5 Hz, C_{11}), 51.7 (d, $^{3}J_{PC}$ = 6.1 Hz, C_{16}), 44.0 (d, $^{3}J_{PC}$ = 4.2 Hz, C_{12}), 40.3 (s, C_{15}), 37.3 (s, C_{17}), 32.7 (d, $^{4}J_{PC}$ = 13.1 Hz, C_{13}), 28.1 (s, C_{14}), 26.7 (d, $^{1}J_{PC}$ = 19.3 Hz, CH*iPr*), 25.6 (d, $^{1}J_{PC}$ = 21.1 Hz, CH*iPr*), 18.9 (s, CH_3*iPr*), 18.5 (d, $^{2}J_{PC}$ = 4.4 Hz, CH_3*iPr*), 18.0 (d, $^{2}J_{PC}$ = 4.1 Hz, CH_3*iPr*), 17.8 (d, $^{2}J_{PC}$ = 4.9 Hz, CH_3*iPr*); **HRMS (ESI+)**: calcd for [M]+ = $C_{29}H_{35}AuP^+$: 611.2141. Found: 611.2144.

Synthesis of Gold(III)–(*p*-methoxyphenyl) complex 4’

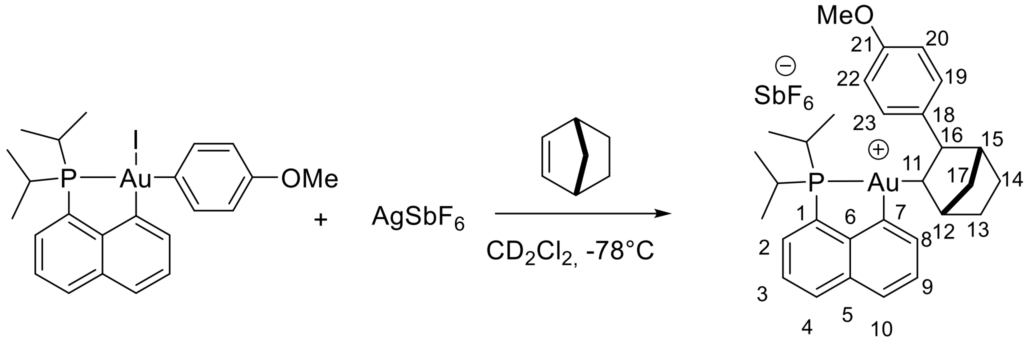

2' **4'**

A solution of $AgSbF_6$ (61.1 mg, 0.178 mmol) in 1.2 mL of DCM was added at -80°C to a mixture of complex **2’** (100.0 mg, 0.148 mmol) and NB (16.8 mg, 0.178 mmol) in DCM (2.8 mL). The reaction was allowed to warm up to room temperature and stirred for 30 min at this temperature (the reaction medium evolved from colorless to yellow). The resulting suspension was filtered via filtrating cannula, evaporated to dryness and washed with pentane. Drying under vacuum afforded the crude compound **4’** as a yellow powder in 65% yield (74 mg). The solid was further purified by crystallization at – 20°C in a saturated DCM/pentane mixture to give single crystals suitable for X-ray diffraction analysis (19% yield, 18 mg).

^{1}H NMR (400 MHz, CD_2Cl_2, 253K): δ 8.22 (dd, $^3J_{HH}$ = 8.3 Hz, $^4J_{HH}$ = 2.3 Hz, 1H, H_{22}), 8.08-8.04 (m, 2H, H_4&H_{20}), 7.87 (d, $^3J_{HH}$ = 8.0 Hz, 1H, H_{10}), 7.83 (d, $^3J_{HH}$ = 7.8 Hz, 1H, H_8), 7.72-7.67 (m, 1H, H_2), 7.63-7.59 (m, 1H, H_3), 7.54 (t, $^3J_{HH}$ = 7.8 Hz, 1H, H_9), 7.37 (dd, $^3J_{HH}$ = 8.8 Hz, $^4J_{HH}$ = 2.8 Hz, 1H, H_{19}), 7.30 (dd, $^3J_{HH}$ = 8.3 Hz, $^4J_{HH}$ = 2.8 Hz, 1H, H_{23}), 4.58 (dd, $^3J_{HH}$ = 8.2 Hz, $^4J_{PH}$ = 11.3 Hz, 1H, H_{16}), 3.97 (s, 3H, OMe), 2.99 (bs, 1H, H_{12}), 2.75-2.64 (m, 2H, H_{15}&CH*i*Pr), 2.38 (m, 1H, H_{11}), 2.06-1.92 (m, 3H, H_{13}&H_{17}&CH*i*Pr), 1.82-1.73 (m, 1H, H_{14}), 1.55 (m, 1H, $H_{13'}$), 1.45 (d, $^3J_{HH}$ = 10.5 Hz, 1H, $H_{17'}$), 1.35 (m, 1H, $H_{14'}$), 1.10-1.03 (m, 9H, CH_3*i*Pr), 0.82 (dd, $^3J_{HH}$ = 7.2 Hz, $^3J_{PH}$ = 19.6 Hz, 3H, CH_3*i*Pr); **^{31}P{^{1}H} NMR** (162 MHz, CD_2Cl_2, 253K): δ 72.2 (s); **^{13}C{^{1}H} NMR** (100 MHz, CD_2Cl_2, 253K): δ 164.4 (s, C_{21}), 145.3 (d, $^2J_{PC}$ = 27.3 Hz, C_6), 145.0 (d, $^3J_{PC}$ = 2.8 Hz, C_7), 140.1 (s, C_{20}), 135.3 (s, C_{22}), 134.5 (d, $^3J_{PC}$ = 11.0 Hz, C_5), 132.9 (d, $^4J_{PC}$ = 2.5 Hz, C_4), 132.2 (s, C_2), 129.4 (s, C_{10}), 128.9 (d, $^4J_{PC}$ = 1.3 Hz, C_8), 127.4 (s, C_9), 126.5 (d, $^3J_{PC}$ = 7.5 Hz, C_3), 122.8 (d, $^3J_{PC}$ = 48.5 Hz, C_1), 119.6 (s, C_{19}), 115.7 (s, C_{23}), 90.3 (s, $^4J_{PC}$ = 8.6 Hz, C_{18}), 56.5 (s, OMe), 55.1 (d, $^2J_{PC}$ = 70.8 Hz, C_{11}), 51.6 (d, $^3J_{PC}$ = 6.3 Hz, C_{16}), 43.5 (d, $^3J_{PC}$ = 4.2 Hz, C_{12}), 41.4 (s, C_{15}), 37.8 (s, C_{17}), 32.6 (d, $^4J_{PC}$ = 13.5 Hz, C_{13}), 28.3 (s, C_{14}), 26.1 (d, $^1J_{PC}$ = 18.9 Hz, CH*i*Pr), 25.9 (d, $^1J_{PC}$ = 21.0 Hz, CH*i*Pr), 18.8 (s, CH_3*i*Pr), 18.0 (s, $^2J_{PC}$ = 4.3 Hz, CH_3*i*Pr), 17.5 (d, $^2J_{PC}$ = 5.1 Hz, CH_3*i*Pr), 17.4 (d, $^2J_{PC}$ = 3.8 Hz, CH_3*i*Pr); **HRMS (ESI+)**: calcd for [M]+ = $C_{30}H_{37}AuOP^+$: 641.2248. Found: 641.2260. **Elemental Analysis**: calcd for $C_{30}H_{37}OAuPSbF_6$: C 41.07, H 4.25. Found: C 41.35, H 4.61.

Synthesis of the neutral tetracoordinate complex 4’-Cl

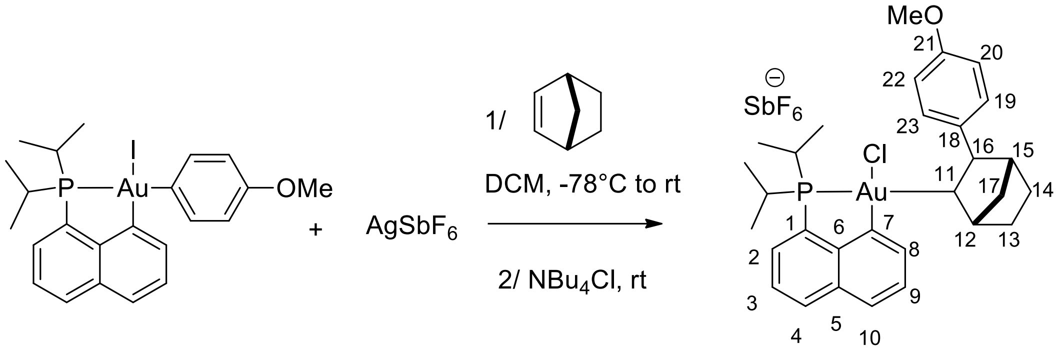

2' **4'-Cl**

A solution of $AgSbF_6$ (50.0 mg, 0.089 mmol) in 0.6 mL of DCM was added at -80°C to a mixture of complex **2’** (50.0 mg, 0.074 mmol) and NB (8.4 mg, 0.089 mmol) in DCM (1.4 mL). The reaction was allowed to warm up to room temperature and stirred for 30 min at this temperature (the reaction medium evolved from colorless to yellow). A solution of NBu_4Cl (24.7 mg, 0.089 mmol) in DCM (0.6 mL) was then added and the medium immediately turned colorless. After filtration on a celite pad, the solvent was removed in vacuo and the crude mixture was purified by flash column chromatography (silica, eluent: Pentane/DCM 100:0 to 50:50) to afford compound as a white solid in 56% yield (28 mg).

^{1}H NMR (400 MHz, CD_2Cl_2, 253K): δ 8.35 (d, $^3J_{HH}$ = 7.5 Hz, 1H H_8), 7.95 (d, $^3J_{HH}$ = 8.0 Hz, 1H, H_4), 7.76 (d, $^3J_{HH}$ = 8.1 Hz, 1H, H_{10}), 7.63 (dd, $^3J_{HH}$ = 7.0 Hz, $^3J_{PH}$ = 7.5 Hz, 1H H_2), 7.54-7.46 (m, 2H, H_3&H_9), 7.32 (br d, $^3J_{HH}$ = 9.5 Hz, 2H, H_{19}&H_{23}), 6.46 (br d, $^3J_{HH}$ = 8.6 Hz, 2H, H_{20}&H_{22}), 3.51 (s, 3H, OMe), 3.46 (dd, $^3J_{HH}$ = 8.2 Hz, $^4J_{PH}$ = 9.3 Hz, 1H, H_{16}), 3.08 (heptd, $^3J_{HH}$ = 7.0 Hz; $^2J_{PH}$ = 10.7 Hz, 1H, CH*iPr*), 2.94 (br s, 1H, H_{12}), 2.89-2.78 (m, 2H, H_{11}&CH*i*Pr), 2.72 (d, $^3J_{HH}$ = 9.8 Hz, H_{14}), 2.18 (br s, 1H, H_{15}), 1.88-1.80 (m, 1H, H_{13}), 1.70-1.62 (m, 1H, H_{17}), 1.43 (m, 1H, $H_{17'}$), 1.34 (m, 2H, $H_{13'}$&$H_{14'}$),1.26-1.07 (m, 9H, CH_3*i*Pr), 0.66 (dd, $^3J_{HH}$ = 7.0 Hz, $^3J_{PH}$ = 16.8 Hz, 3H, CH_3*i*Pr); **^{31}P{^{1}H} NMR** (162 MHz, CD_2Cl_2, 253K): δ 73.5 (s); **^{13}C{^{1}H} NMR** (100 MHz, CD_2Cl_2, 253K): δ 156.7 (s, C_{21}), 148.5 (d, $^2J_{PC}$ = 34.0 Hz, C_6), 145.6 (d, $^3J_{PC}$ = 3.8 Hz, C_7), 141.8 (d, $^4J_{PC}$ = 9.4 Hz, C_{18}), 134.4 (d, $^3J_{PC}$ = 13.9 Hz, C_5), 132.4 (d, $^4J_{PC}$ = 2.1 Hz, C_4), 131.3 (s, C_8), 130.6 (s, C_2), 129.8 (s, C_{19}&C_{23}), 127.8 (d, $^3J_{PC}$ = 44.2 Hz, C_1), 127.2 (s, C_9), 127.0 (s, C_{10}), 125.5 (d, $^3J_{PC}$ = 7.1 Hz, C_3), 112.9 (s, C_{20}&C_{22}), 72.3 (d, $^2J_{PC}$ = 106.3, C_{11}), 56.2 (d, $^3J_{PC}$ = 5.0 Hz, C_{16}), 54.9 (s, OMe), 44.8 (s, C_{15}), 43.3 (d, $^3J_{PC}$ = 2.9 Hz, C_{12}), 38.4 (s, C_{14}), 33.5 (d, $^4J_{PC}$ = 13.1 Hz, C_{13}), 31.2 (s, C_{17}), 25.9 (d, $^1J_{PC}$ = 21.3 Hz, CH*i*Pr), 24.9 (d, $^1J_{PC}$ = 22.1 Hz, CH*i*Pr), 17.9 (d, $^2J_{PC}$ = 3.4 Hz, CH_3*i*Pr), 17.8 (d, $^2J_{PC}$ = 3.7 Hz, CH_3*i*Pr), 17.7 (d, $^2J_{PC}$ = 3.7 Hz, CH_3*i*Pr), 17.7 (s, CH_3*i*Pr); **HRMS (ESI+)**: calcd for [M]+ = 641.2248, found: 641.2251.

Synthesis of the cyclometallated complex 5

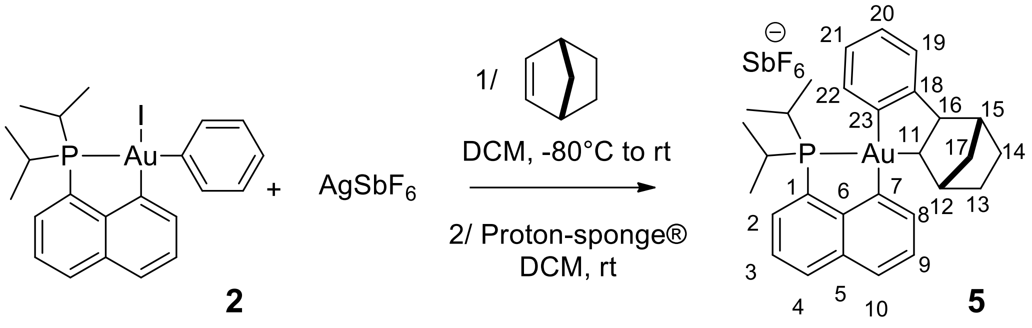

A solution of $AgSbF_6$ (19.2 mg, 0.056 mmol) in 0.4 mL of DCM was added at – 80°C to a mixture of complex **2** (30.0 mg, 0.047 mmol) and a 1M solution of NB in DCM (56 µL, 0.056 mmol) in DCM (1.2 mL). The reaction was allowed to warm up to room temperature and stirred for 10 min at this temperature (colored evolved from colorless to yellow). A solution of Proton-sponge® (20.0 mg, 0.093 mmol) in 0.4 mL of DCM was then added and color evolved to light green solution and black suspension. The mixture was filtered on a celite pad before being purified by flash column chromatography (silica, eluent: Pentane/DCM 100:0 to 70:30) to afford complex **5** as a white solid in 95% yield (27 mg).

MP: 186°C. **^{1}H NMR** (500 MHz, CD_2Cl_2): δ 8.37 (d, $^3J_{HH}$ = 6.9 Hz, 1H, H_8), 7.98 (d, $^3J_{HH}$ = 8.0 Hz, 1H, H_3), 7.94 (d, $^3J_{HH}$ = 7.9 Hz, 1H, H_{22}), 7.83 (d, $^4J_{HH}$ = 7.8 Hz, 1H, H_4), 7.77 (d, 1H, $^3J_{HH}$ = 8.2 Hz, H_{10}), 7.60 (t, $^3J_{HH}$ = 7.4 Hz, 1H, H_9), 7.55 (m, 1H, H_2), 7.30 (d, $^3J_{HH}$ = 7.6 Hz, 1H, H_{19}), 7.14 (td, $^3J_{HH}$ = 7.5 Hz, $^4J_{HH}$ = 1.2 Hz, 1H, H_{20}), 7.02 (td, $^3J_{HH}$ = 7.4 Hz, $^4J_{HH}$ = 0.8 Hz, 1H, H_{21}), 7.06 (d, $^3J_{HH}$ = 7.4 Hz, 1H, H_8), 3.52 (dd, $^3J_{HH}$ = 7.2 Hz, $^4J_{PH}$ = 7.2 Hz, 1H, H_{16}), 3.44-3.42 (m, 1H, H_{11}), 3.09 (heptd, $^3J_{HH}$ = 7.0 Hz; $^2J_{PH}$ = 10.3 Hz, 1H, CH*iPr*), 2.98 (heptd, $^3J_{HH}$ = 7.1 Hz; $^2J_{PH}$ = 8.3 Hz, 1H, CH*iPr*), 2.77 (br s, 1H, H_{12}), 2.34 (d, $^3J_{HH}$ = 3.4 Hz 1H, H_{15}), 1.88-1.80 (m, 1H, H_{13}), 1.70-1.63 (m, 2H, $H_{13'}$&H_{14}), 1.62-1.58 (m, 1H, H_{17}), 1.53-1.47 (m, 1H, $H_{14'}$), 1.36 (dd, $^3J_{HH}$ = 7.1 Hz; $^3J_{PH}$ = 17.5 Hz, 3H, CH_3*i*Pr), 1.28-1.21 (m, 6H, CH_3*i*Pr), 1.15 (dd, $^3J_{HH}$ = 7.2 Hz; $^3J_{PH}$ = 18.1 Hz, 3H, CH_3*i*Pr), 0.95-0.90 (m, 1H, $H_{17'}$); **^{31}P{^{1}H} NMR** (202 MHz, CD_2Cl_2): δ 65.5 (s); **^{13}C{^{1}H} NMR** (125 MHz, CD_2Cl_2): δ 170.2 (d, $^2J_{PC}$ = 12.8 Hz, C_{23}), 167.6 (d, $^3J_{PC}$ = 3.1 Hz, C_7), 162.3 (d, $^3J_{PC}$ = 6.0 Hz, C_{18}), 152.1 (d, $^2J_{PC}$ = 30.0 Hz, C_6), 140.0 (d, $^3J_{PC}$ = 5.2 Hz, C_{22}), 134.7 (d, $^3J_{PC}$ = 12.0 Hz, C_5), 134.4 (d, $^3J_{PC}$ = 6.9 Hz, C_8), 133.2 (d, $^1J_{PC}$ = 45.9 Hz, C_1), 132.6 (d, $^3J_{PC}$ = 2.3 Hz, C_3), 130.7 (s, C_4), 127.7 (s, C_9), 125.8 (s, C_{20}), 125.6 (s, C_{10}), 125.3 (d, $^2J_{PC}$ = 7.0 Hz, C_2), 125.0 (d, $^4J_{PC}$ = 2.0 Hz, C_{21}), 124.8 (s, C_{19}), 74.3 (d, $^2J_{PC}$ = 97.2 Hz, C_{11}), 62.5 (d, $^3J_{PC}$ = 5.5 Hz, C_{16}), 47.4 (s, C_{15}), 45.0 (d, $^3J_{PC}$ = 3.4 Hz, C_{12}), 34.5 (s, C_{17}), 33.5 (d, $^4J_{PC}$ = 12.6 Hz, C_{13}), 29.2 (s, C_{14}), 26.4 (d, $^1J_{PC}$ = 18.3 Hz, CH*i*Pr), 26.3 (d, $^1J_{PC}$ = 20.6 Hz, CH*i*Pr), 19.3 (d, $^2J_{PC}$ = 4.5 Hz, CH_3*iPr*), 19.2 (d, $^2J_{PC}$ = 5.2 Hz, CH_3*iPr*), 18.9 (s, CH_3*iPr*), 18.4 (d, $^2J_{PC}$ = 2.8 Hz, CH_3*iPr*); **HRMS (ESI+)**: calcd for $[M]^+$ = $C_{29}H_{35}AuP^+$: 611.2142. Found: 611.2141; **Elemental Analysis**: calcd for $C_{29}H_{34}AuP$: C 57.60, H 5.96. Found: C 57.49, H 5.95.

Characterization of Gold(III) complex 6

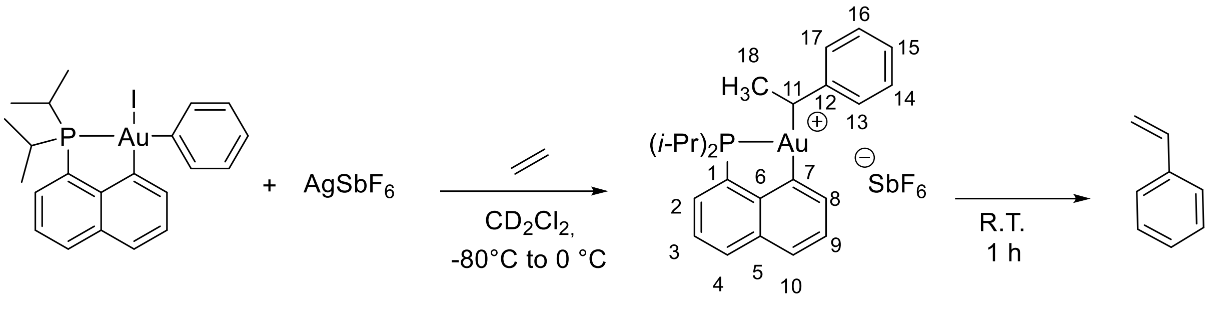

In a glovebox, a pressure tube was charged with $AgSbF_6$ (8.4 mg, 0.024 mmol) in dichloromethane-d^2 (0.35 mL). Complex **2** (15.6 mg, 0.024 mmol) was transferred into a small glass vial and dissolved in dichloromethane-d^2 (0.35 mL). The prepared solution was loaded into a plastic syringe equipped with stainless steel needle. The syringe was closed by blocking the needle with a septum. Outside of the glovebox, the NMR tube was put under positive argon pressure and cooled down to – 80 °C (Acetone/N_2 coldbath). At this temperature, the solution of complex **2** was added. The tube was kept at -80 °C and the solution was degased 3 times using the Freeze-Pump-Thaw degassing technique and 2 bars of ethylene were added. Complex **5** was immediately formed at – 80 °C and was characterized at 0 °C. Complex **5** decomposes at room temperature after 1h to give styrene and diphosphine cationic gold(I) complex.

^{1}H NMR (500 MHz, CD_2Cl_2, 273K): δ 8.09 (m, 2H, H_{14}&H_4), 7.99 (t, $^3J_{HH}$ = 6.8 Hz, 1H, H_{16}), 7.82-7.93 (m, 3H, H_2&H_{10}&H_{15}), 7.67 (m, 1H, H_9), 7.49 (d, $^3J_{HH}$ = 6.8 Hz, 1H, H_{17}), 7.33 (d, $^3J_{HH}$ = 7.2 Hz, 1H, H_8), 7.18 (m, 1H, H_3), 6.49 (dd, $^3J_{HH}$ = 6.8 Hz, $^3J_{PH}$ = 3.4 Hz, 1H, H_{13}), 3.94 (m, 1H, H_{11}), 3.16 (m, 1H, CH*i*Pr), 2.96 (m, 1H, CH*i*Pr), 2.47 (dd, $^3J_{HH}$ = 6.7 Hz, $^4J_{PH}$ = 6.2 Hz, 3H, H_{18}), 1.55 (m, 6H, CH_3*iPr*), 0.97-1.05 (m, 3H, CH_3*iPr*), 0.71-0.79 (m, 6H, CH_3*iPr*) ; **^{31}P{^{1}H} NMR** (202 MHz, CD_2Cl_2, 273K): δ 95.9 (s); **^{13}C{^{1}H} NMR** (125 MHz, CD_2Cl_2, 273K): 156.2 (d, $^3J_{PC}$ = 8.8 Hz, C_7), 148.6 (d, $^2J_{PC}$ = 19.8 Hz, C_6), 136.9 (s, C_{16}), 135.7 (d, J_{PC} = 6.0 Hz, C_{14}), 134.5 (d, $^3J_{PC}$ = 10.7 Hz, C_5), 134.3 (d, J_{PC} = 6.0 Hz, C_{15}), 134.1 (d, $^4J_{PC}$ = 6.8 Hz, C_4), 131.8 (d, $^2J_{PC}$ = 4.0 Hz, C_2), 130.6 (s, C_8), 127.9 (s, C_{10}), 127.8 (s, C_9), 126.9 (d, $^1J_{PC}$ = 61.4 Hz, C_1), 126.6 (d, $^3J_{PC}$ = 10.6 Hz, C_3), 124.2 (d, $^3J_{PC}$ = 4.4 Hz, C_{12}), 123.7 (d, $^4J_{PC}$ = 5.2 Hz, C_{17}), 113.54 (d, J = 10.3 Hz, C_{13}), 68.6 (d, $^2J_{PC}$ = 7.7 Hz, C_{11}), 27.7 (d, $^1J_{PC}$ = 28.0 Hz, CH*iPr*), 26.2 (d, $^1J_{PC}$ = 28.3 Hz, CH*iPr*), 18.6 (d, $^2J_{PC}$ = 8.0 Hz, CH_3*iPr*), 18.1 (s, CH_3*iPr*), 17.1 (d, $^2J_{PC}$ = 6.3 Hz, CH_3*iPr*), 14.1 (s, CH_3, C_{18}); **HRMS (ESI+)**: calcd for $[M]^+$ = $C_{24}H_{29}AuP^+$: 545.1672. Found: 545.1678.

NMR spectra of complexes

Figure S1. ^{1}H NMR spectrum of complex **2** (298K)

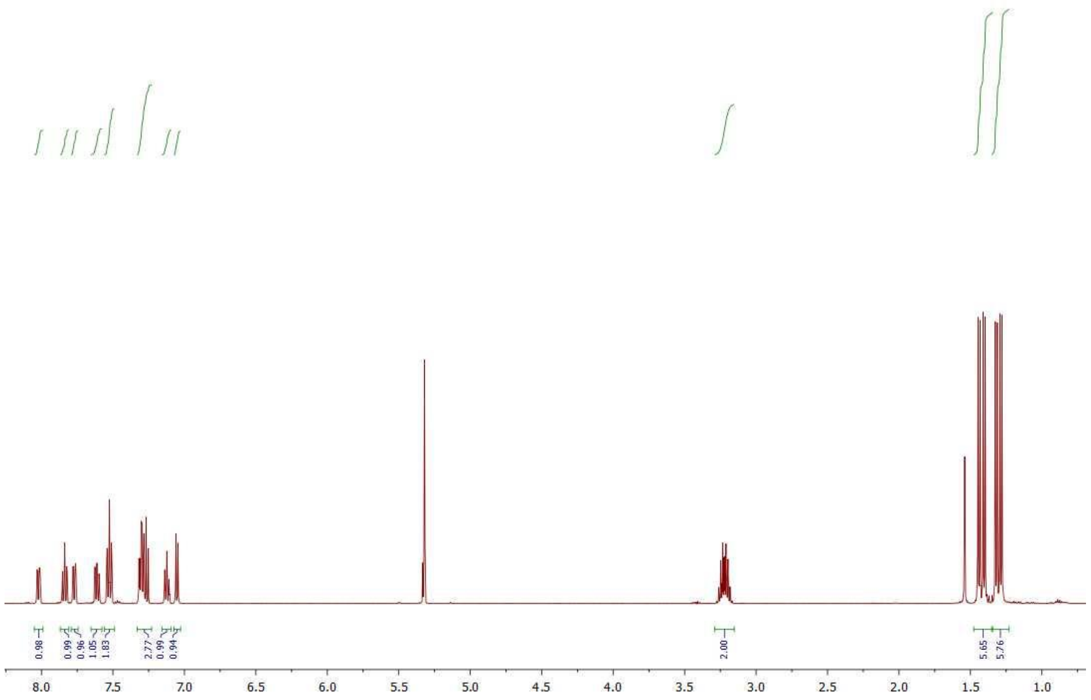

Figure S2 ^{31}P{^{1}H} NMR spectrum of complex **2** (298K)

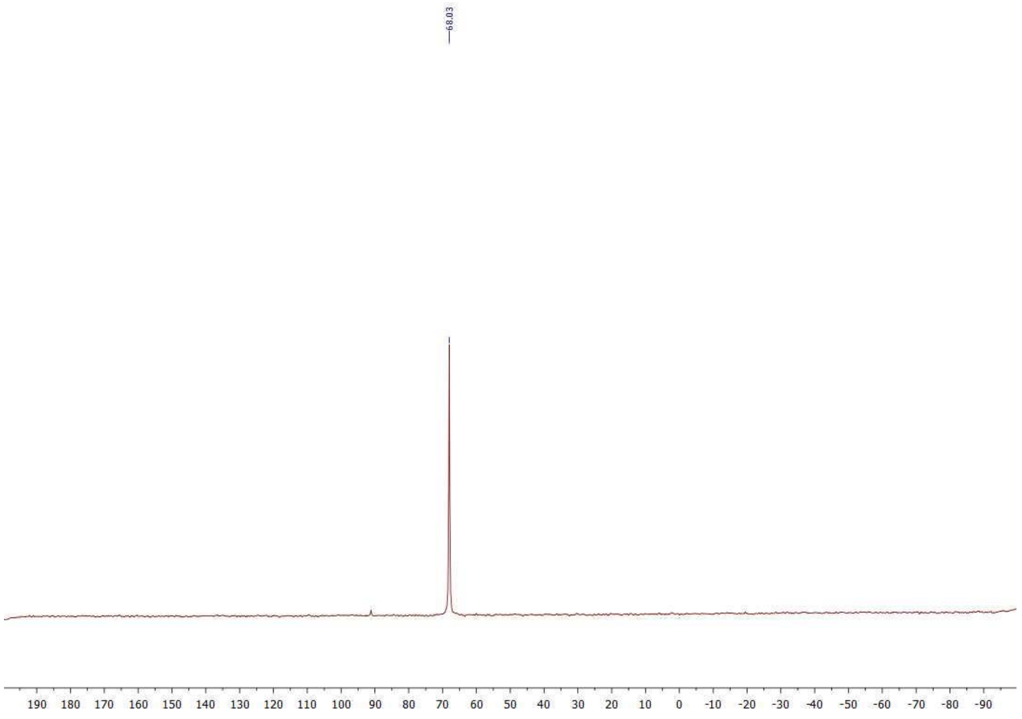

Figure S3. $^{13}C\{^{1}H\}$ NMR spectrum of complex **2** (298K)

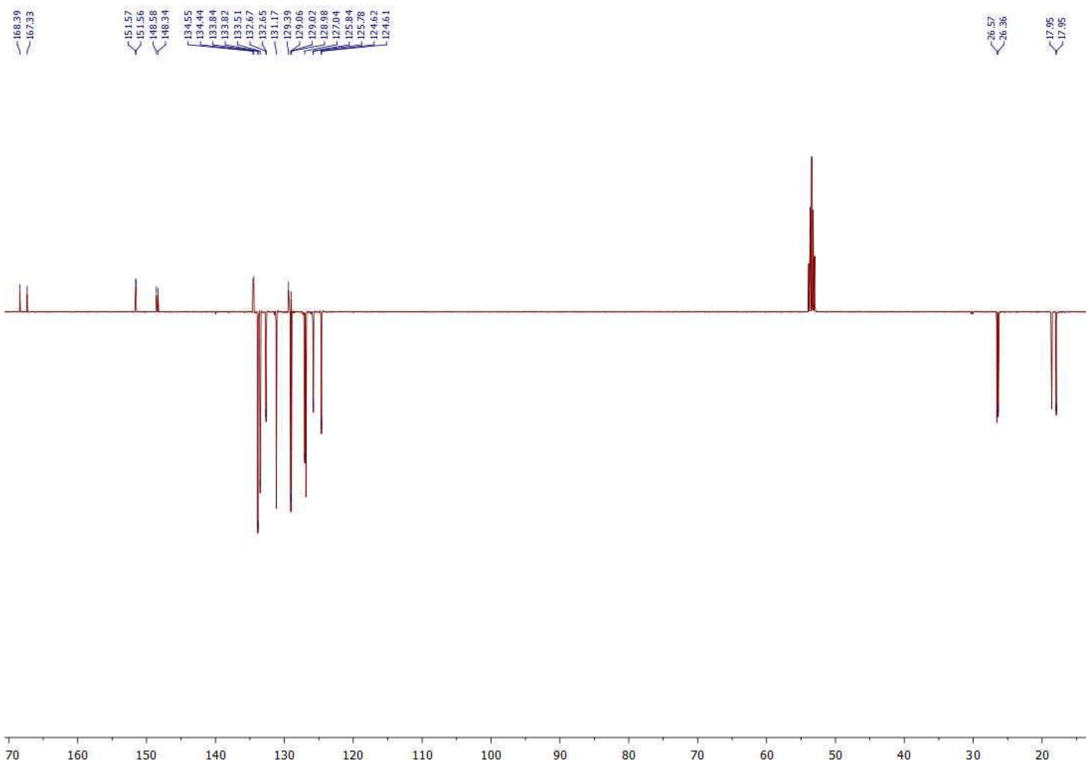

Figure S4. ^{1}H NMR spectrum of complex **2’** (298K)

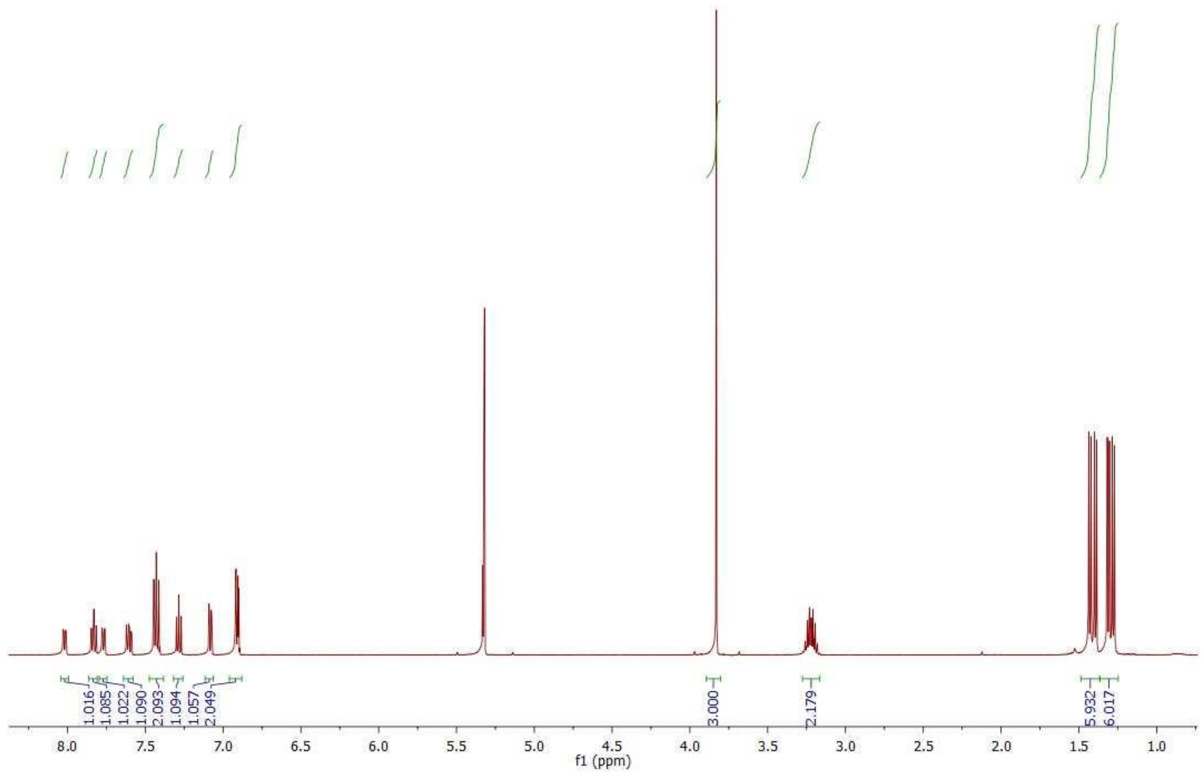

Figure S5 $^{31}P\{^{1}H\}$ NMR spectrum of complex **2’** (298K)

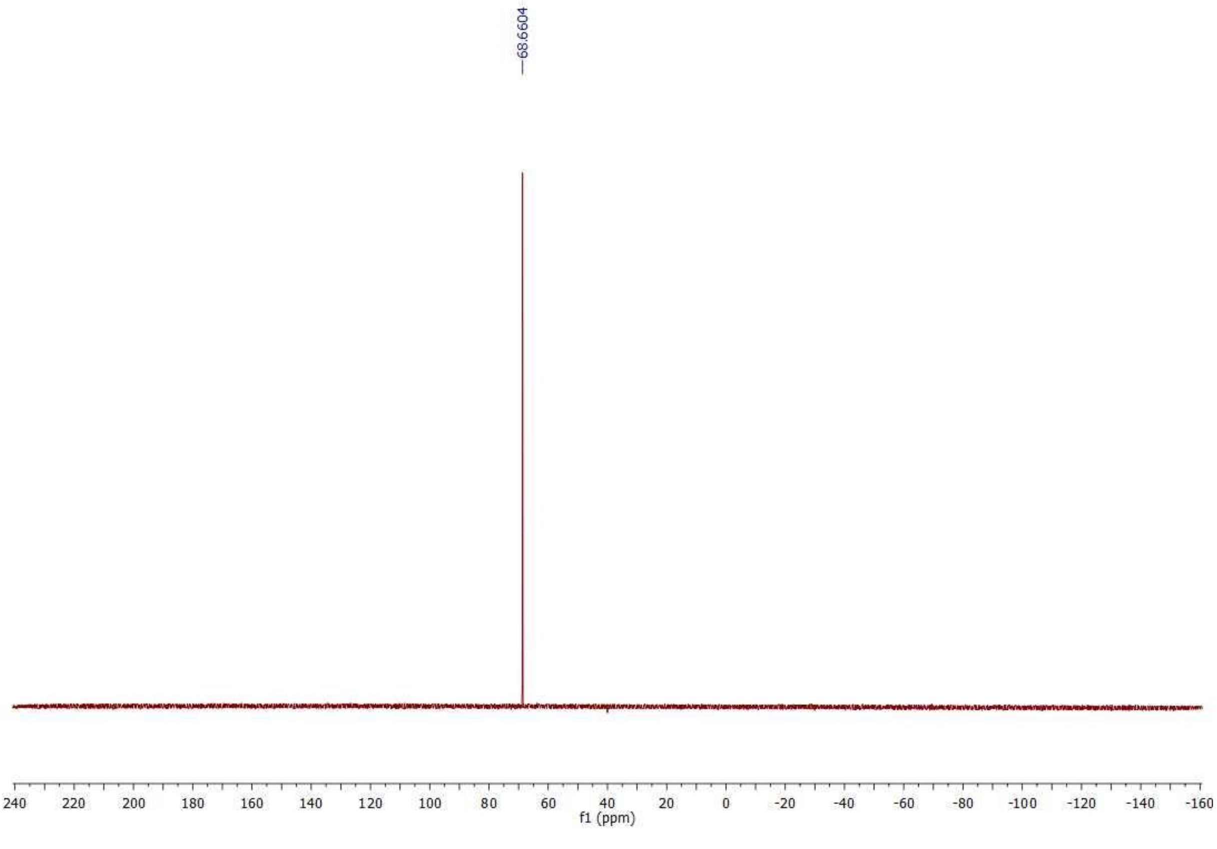

Figure S6. $^{13}C\{^{1}H\}$ NMR spectrum of complex **2'** (298K)

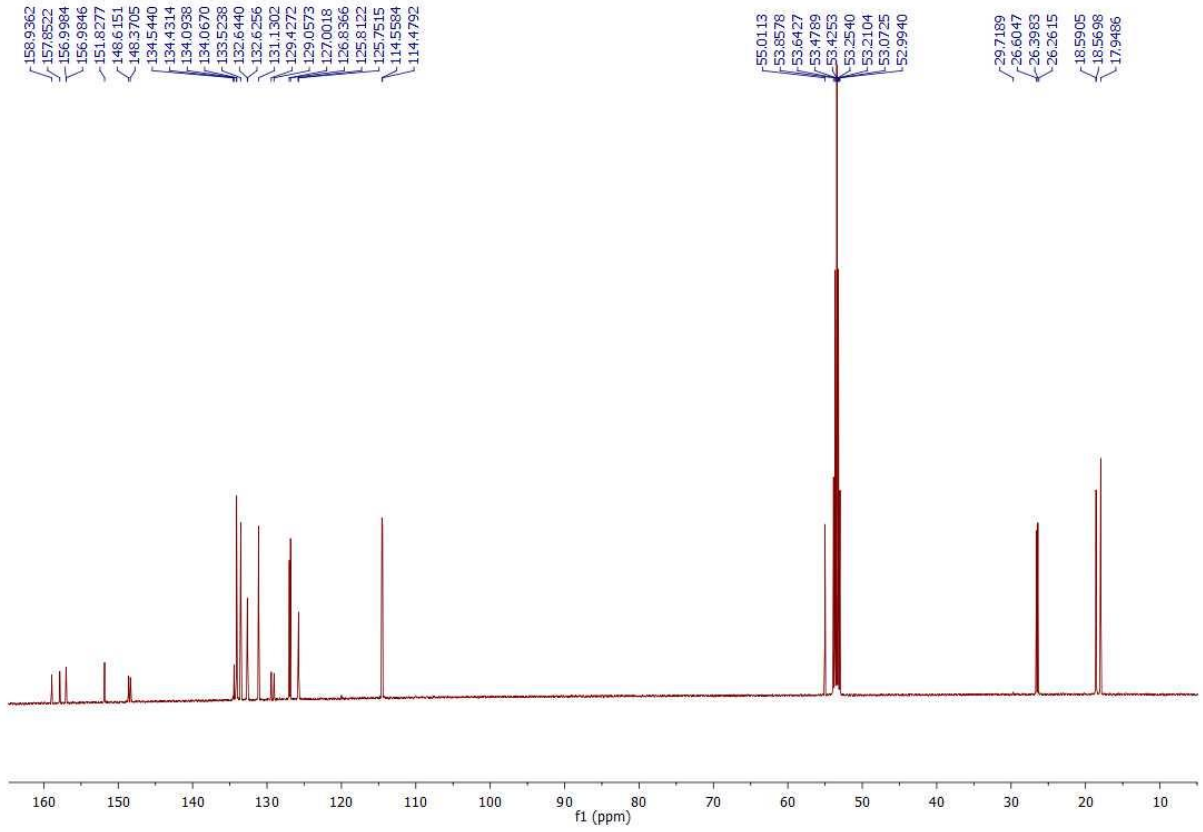

Figure S7. ^{1}H NMR spectrum of complex **3** (213K)

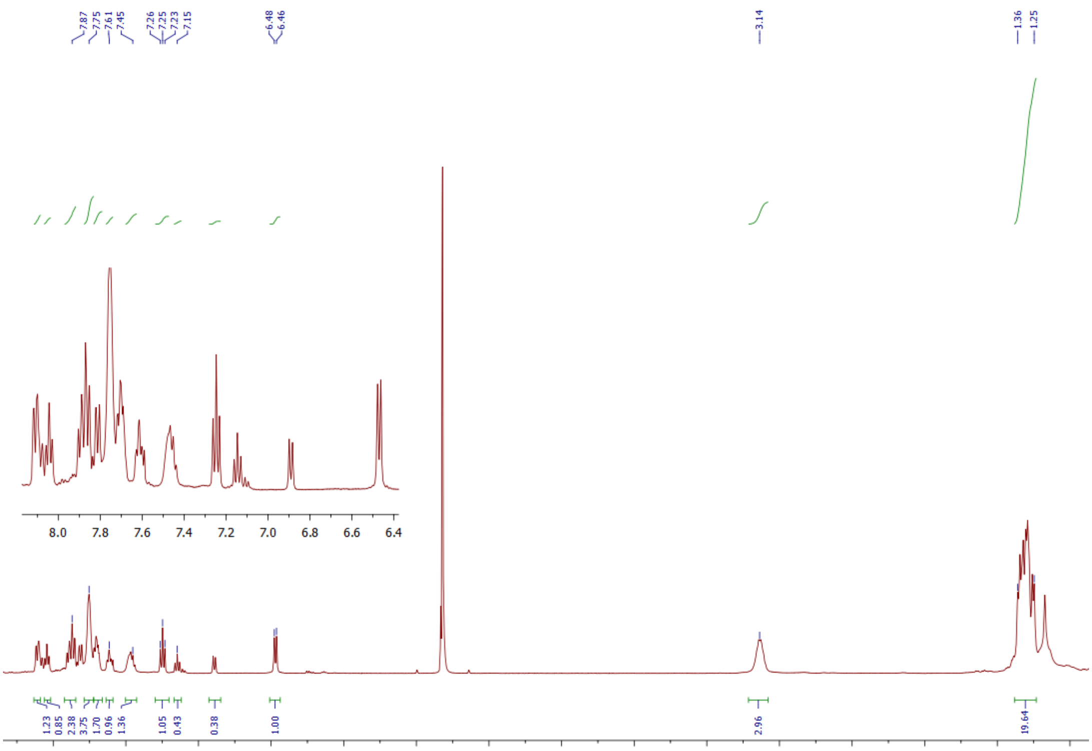

Figure S8. $^{31}P\{^{1}H\}$ NMR spectrum of complex **3** (213K)

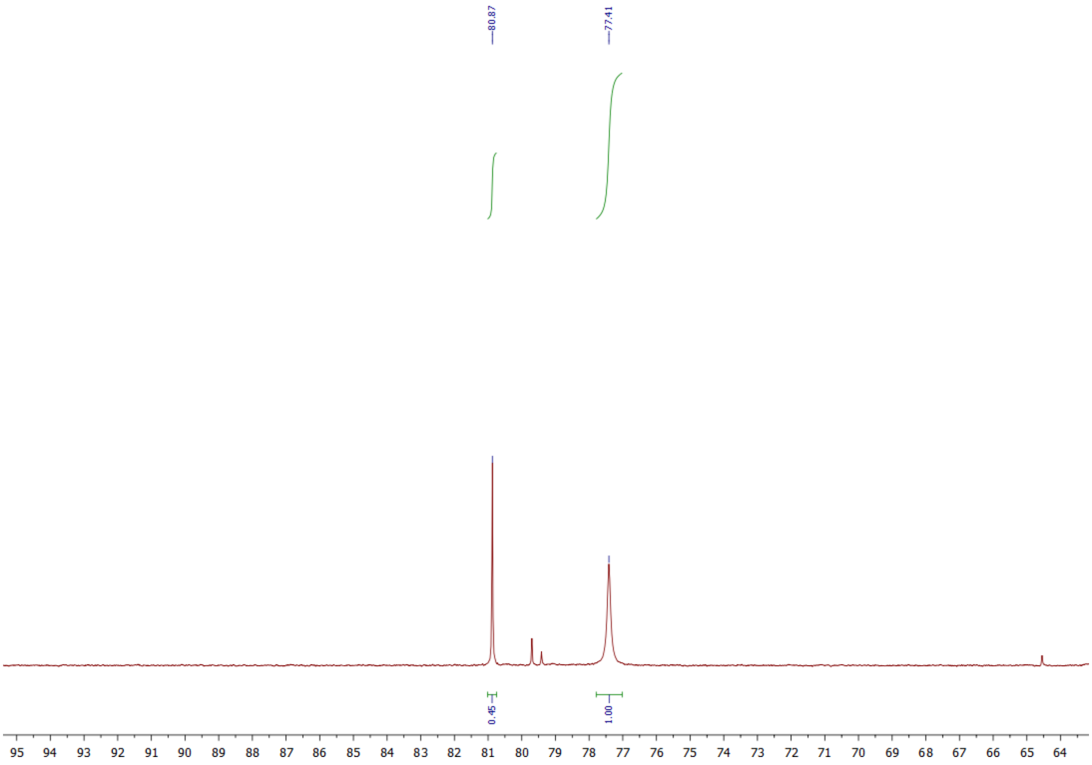

Figure S9. $^{13}C\{^{1}H\}$ NMR spectrum of complex **3** (213K)

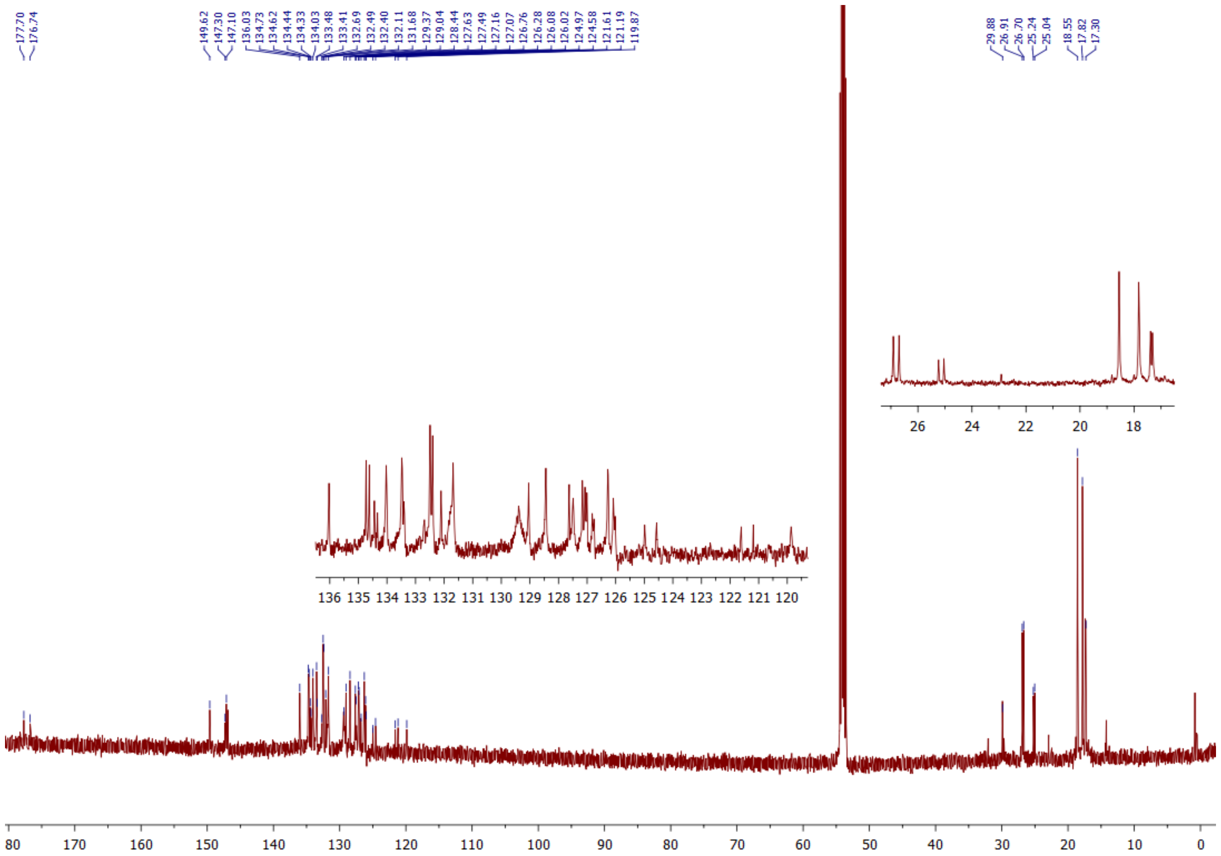

Figure S10. EXSY $^{31}P\{^{1}H\}$-$^{31}P\{^{1}H\}$ (Bruker sequence NOESY PHDC) spectrum of complex **3** (213K)

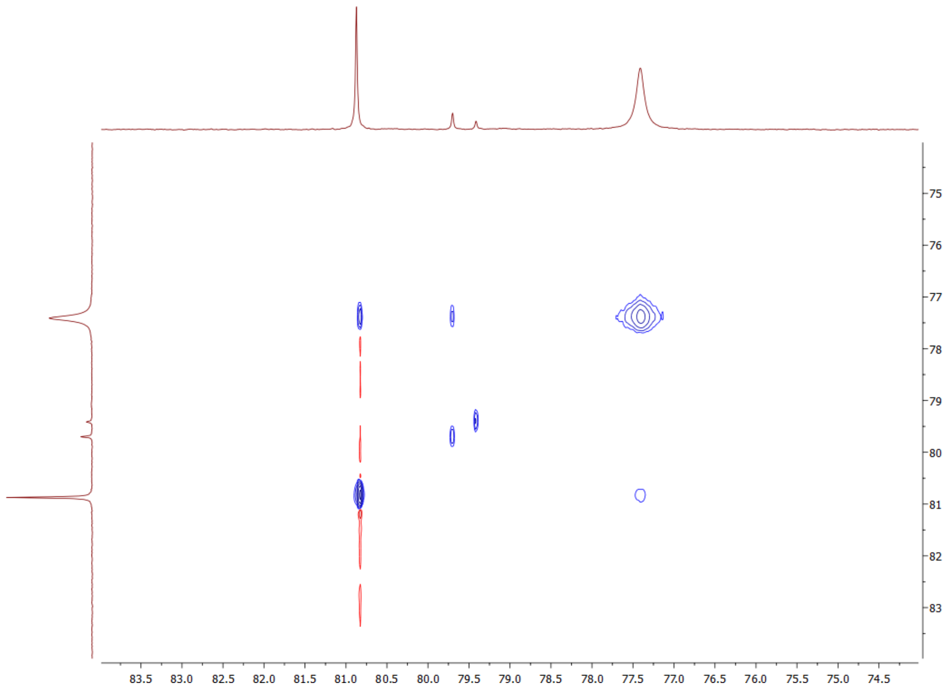

Figure S11. ^{1}H NMR spectrum of complex **4** (253K)

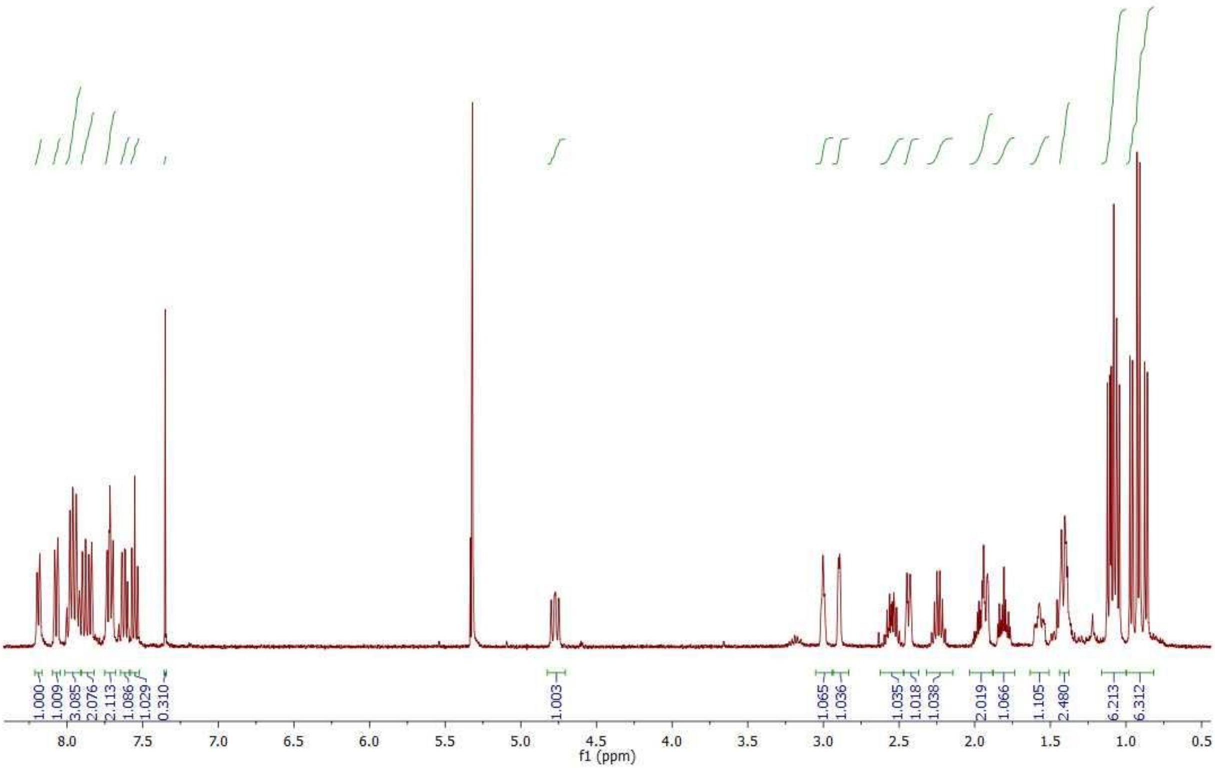

Figure S12. ^{31}P{^{1}H} NMR spectrum of complex **4** (253K)

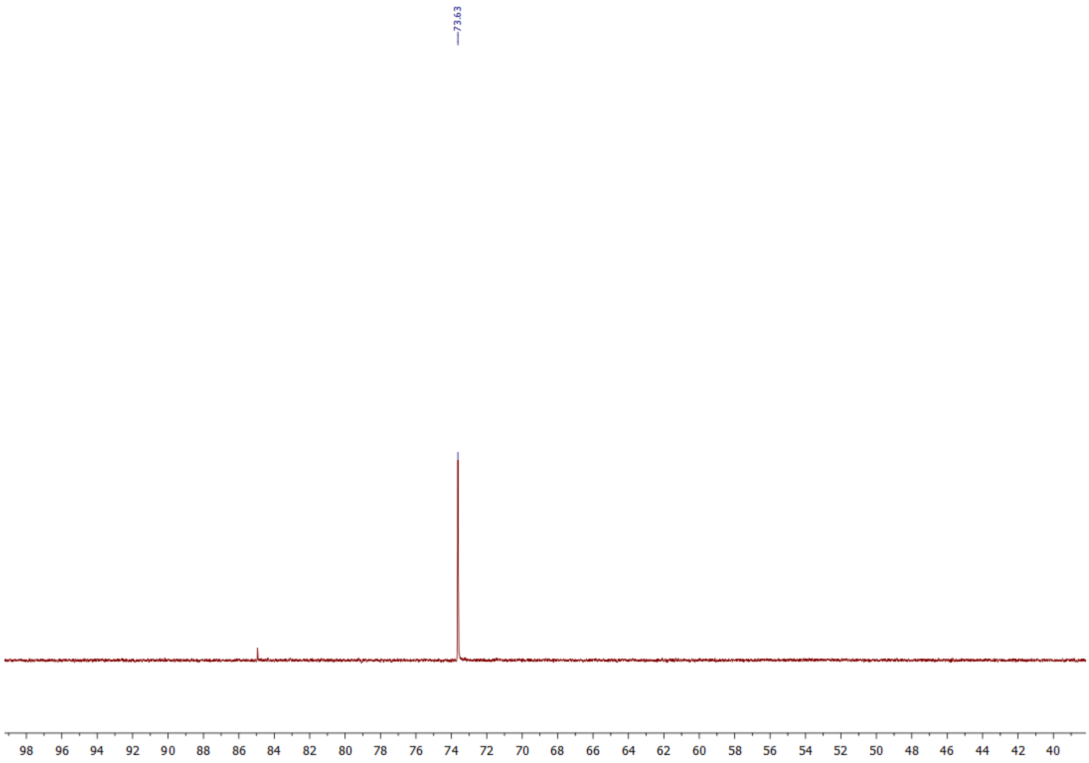

Figure S13. $^{13}C\{^1H\}$ NMR spectrum of complex **4** (253K)

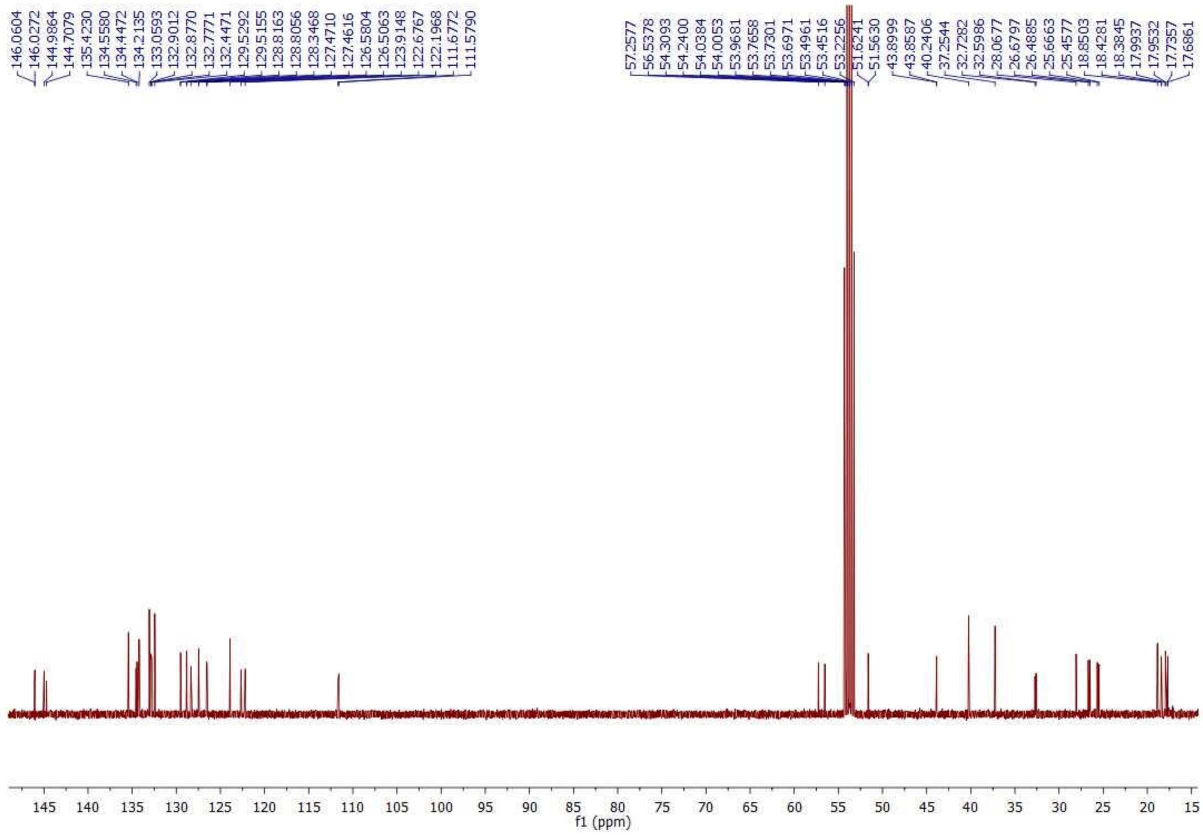

Figure S14. ^{1}H NMR spectrum of complex **4’** (253K)

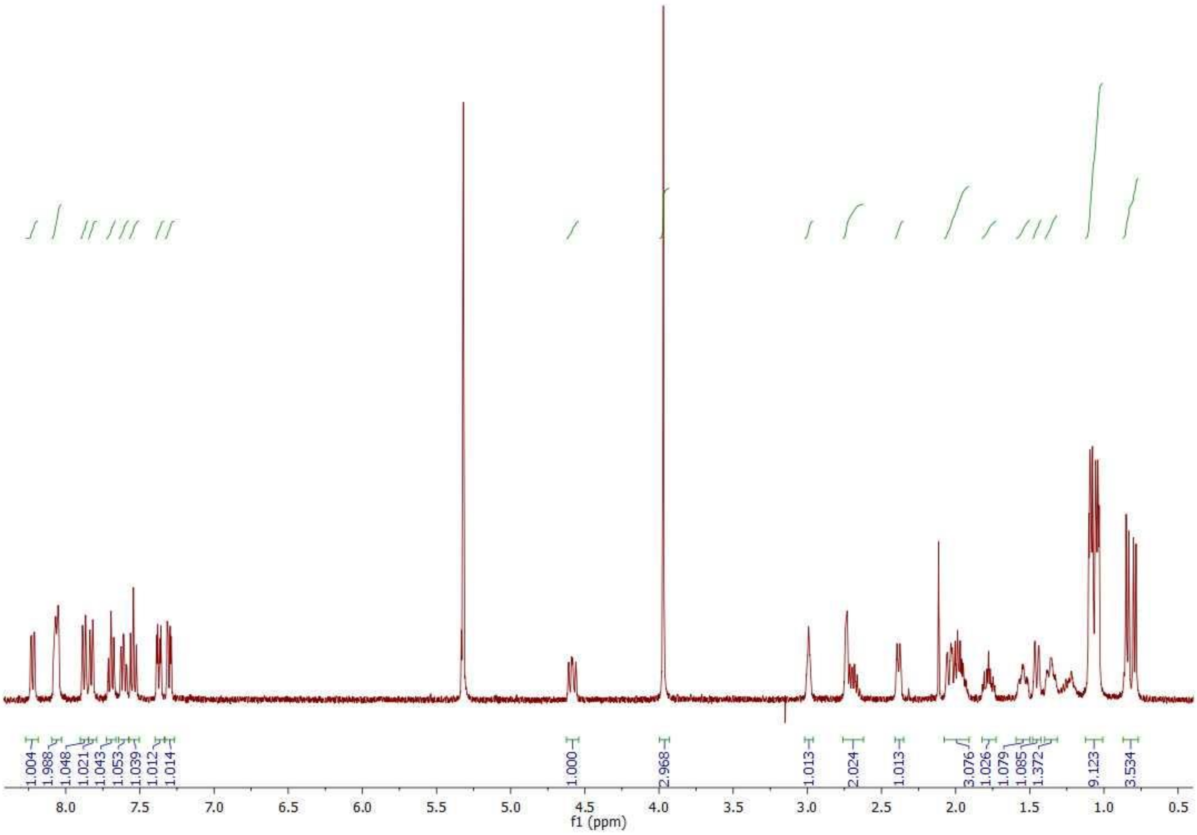

Figure S15. $^{31}P\{^{1}H\}$ NMR spectrum of complex **4’** (253K)

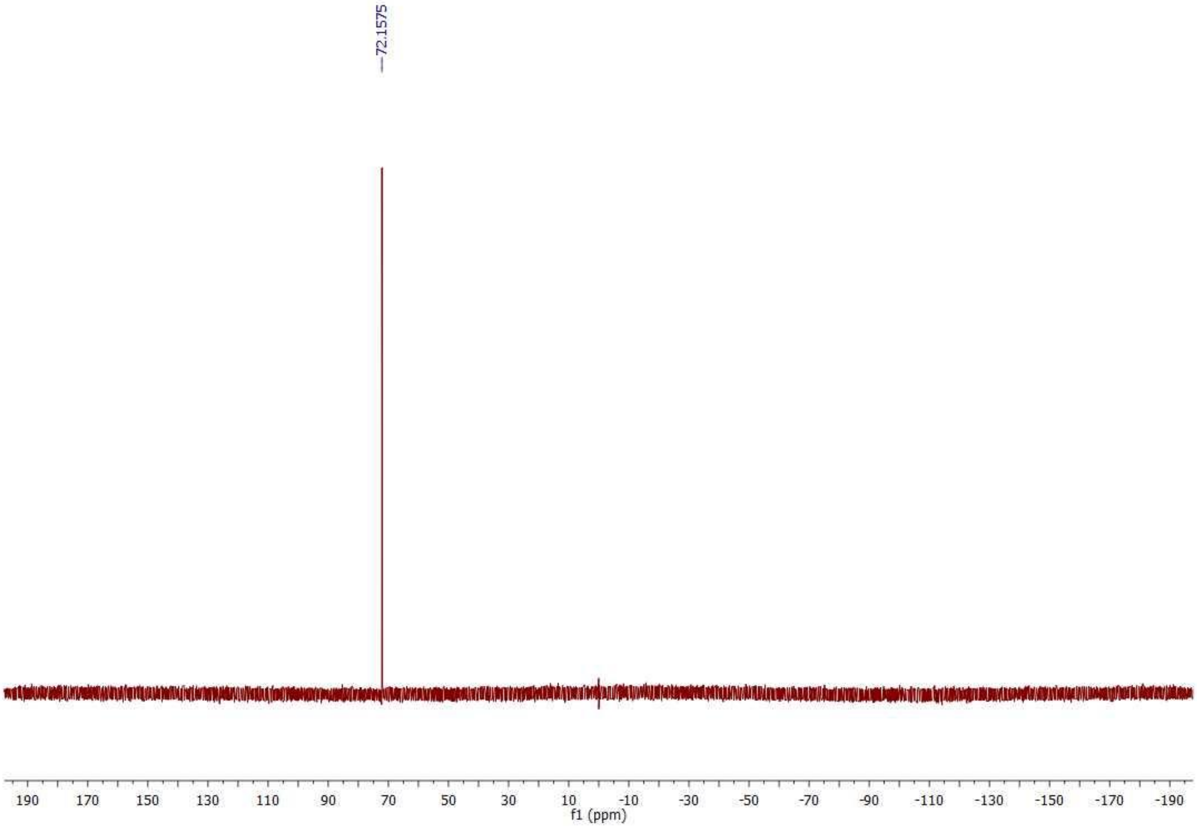

Figure S16. $^{13}C\{^{1}H\}$ NMR spectrum of complex **4’** (253K)

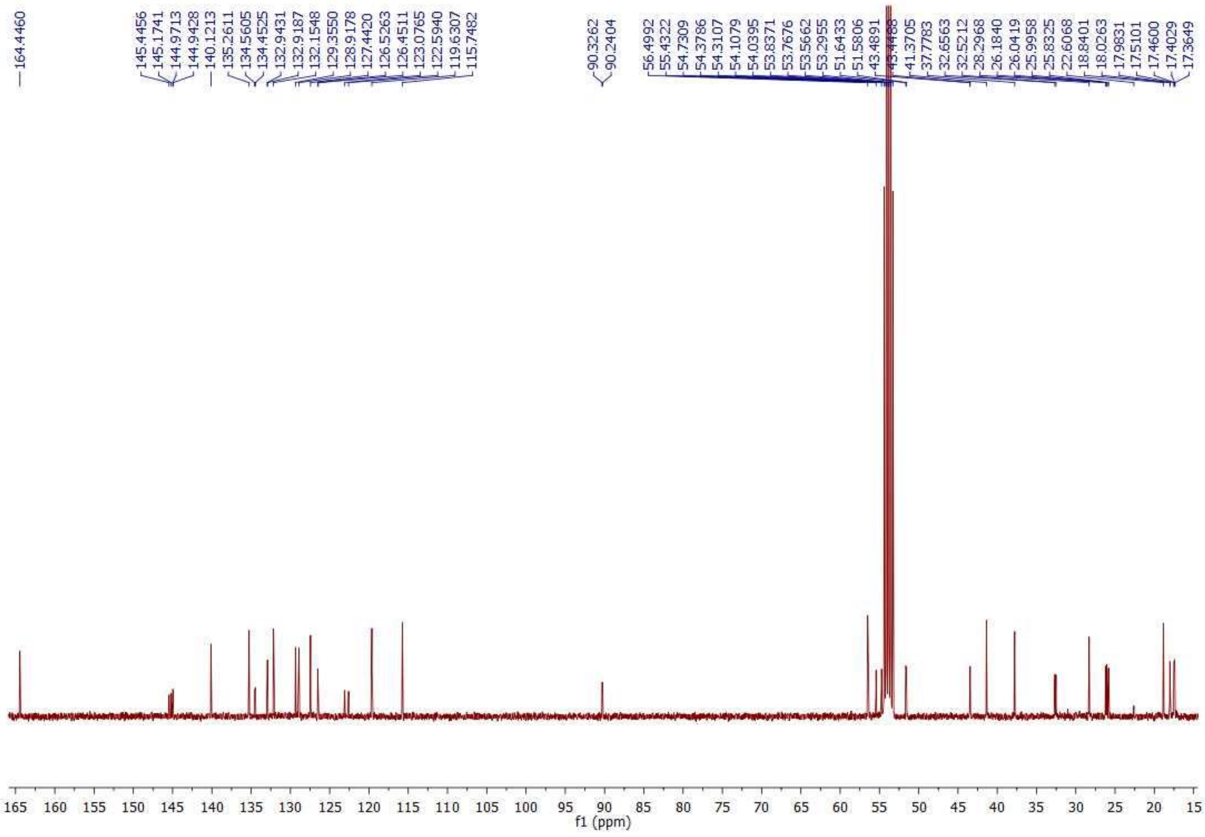

Figure S17. ^{1}H NMR spectrum of complex **4’-Cl** (253K)

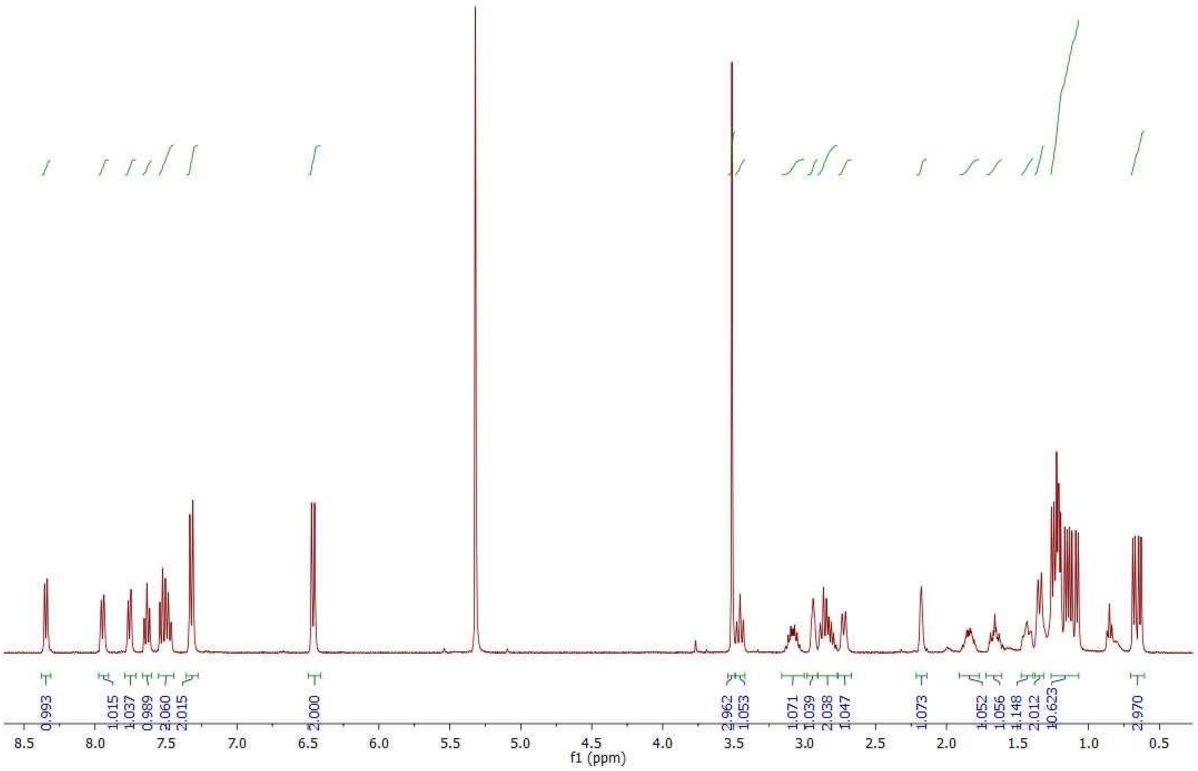

Figure S18. ^{31}P{^{1}H} NMR spectrum of complex **4’-Cl** (253K)

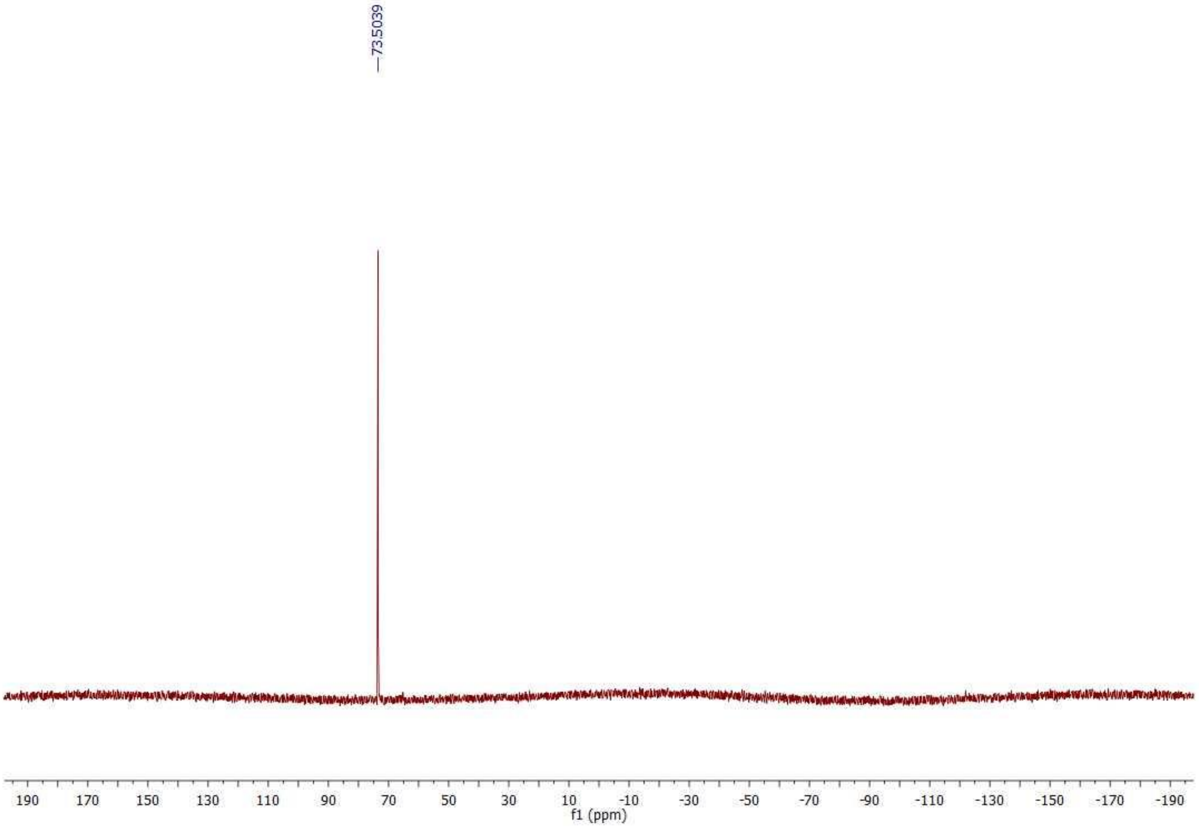

Figure S19. $^{13}C\{^{1}H\}$ NMR spectrum of complex **4’-Cl** (253K)

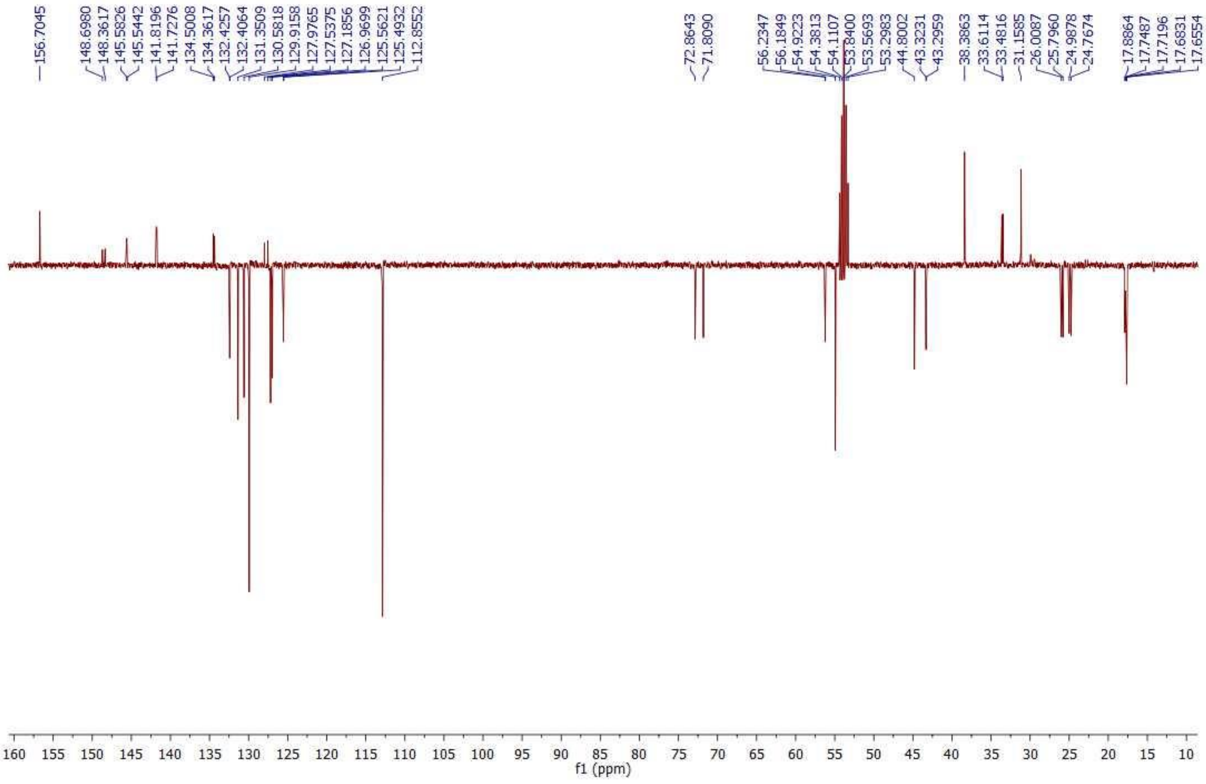

Figure S20. ^{1}H NMR spectrum of complex **5** (298K)

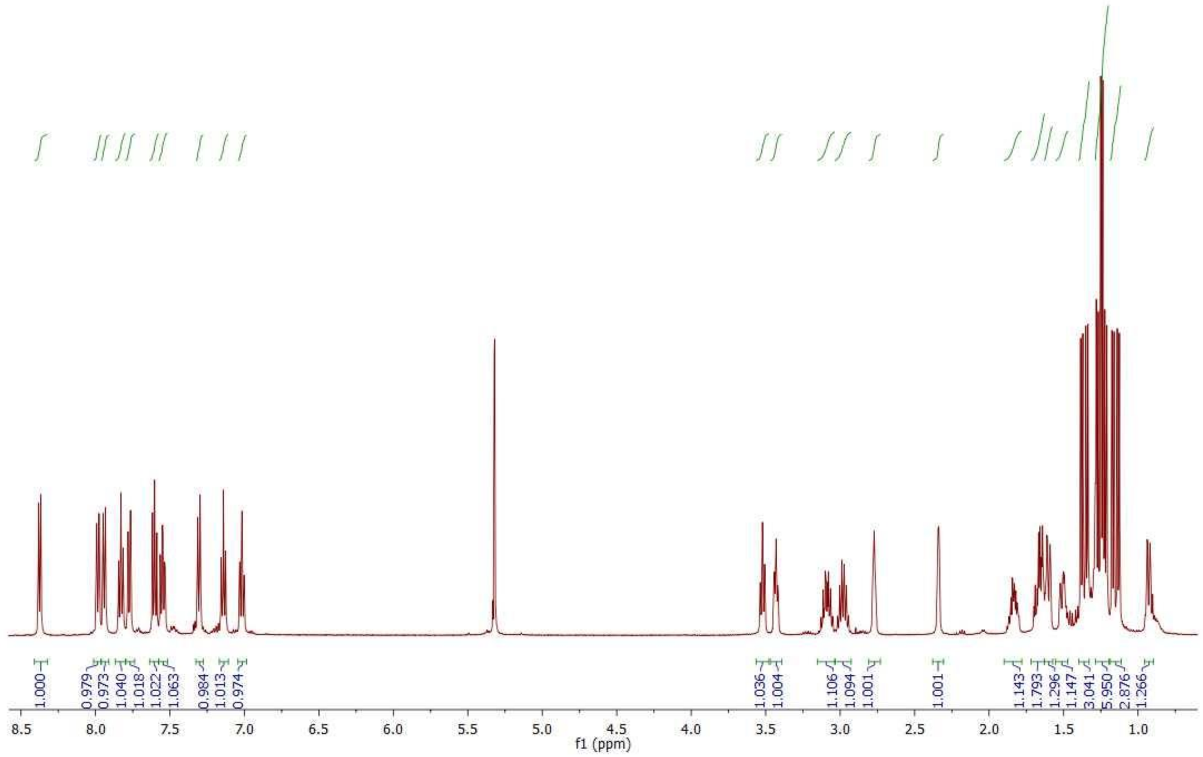

Figure S21. ^{31}P{^{1}H} NMR spectrum of complex **5** (298K)

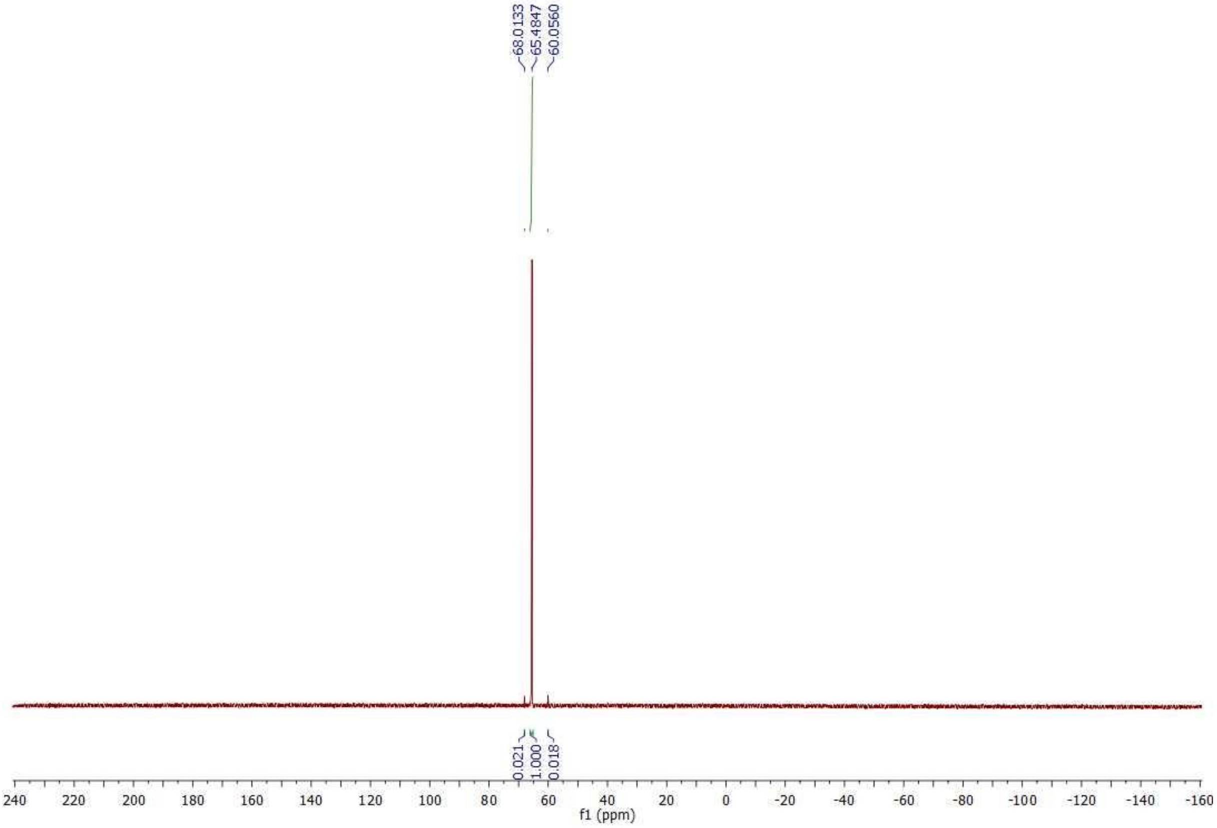

Figure S22. $^{13}C\{^{1}H\}$ NMR spectrum of complex **5** (298K)

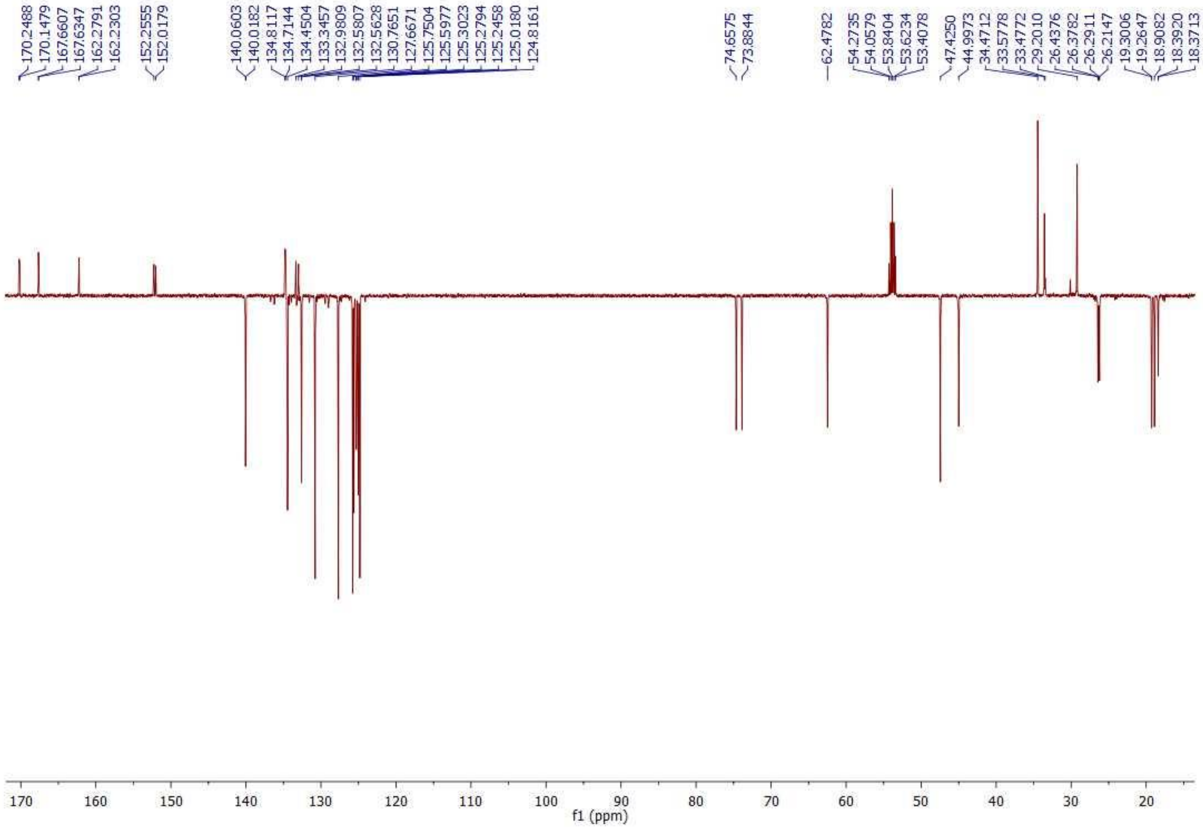

Figure S23. ^{1}H NMR spectrum of complex **6** (273K)

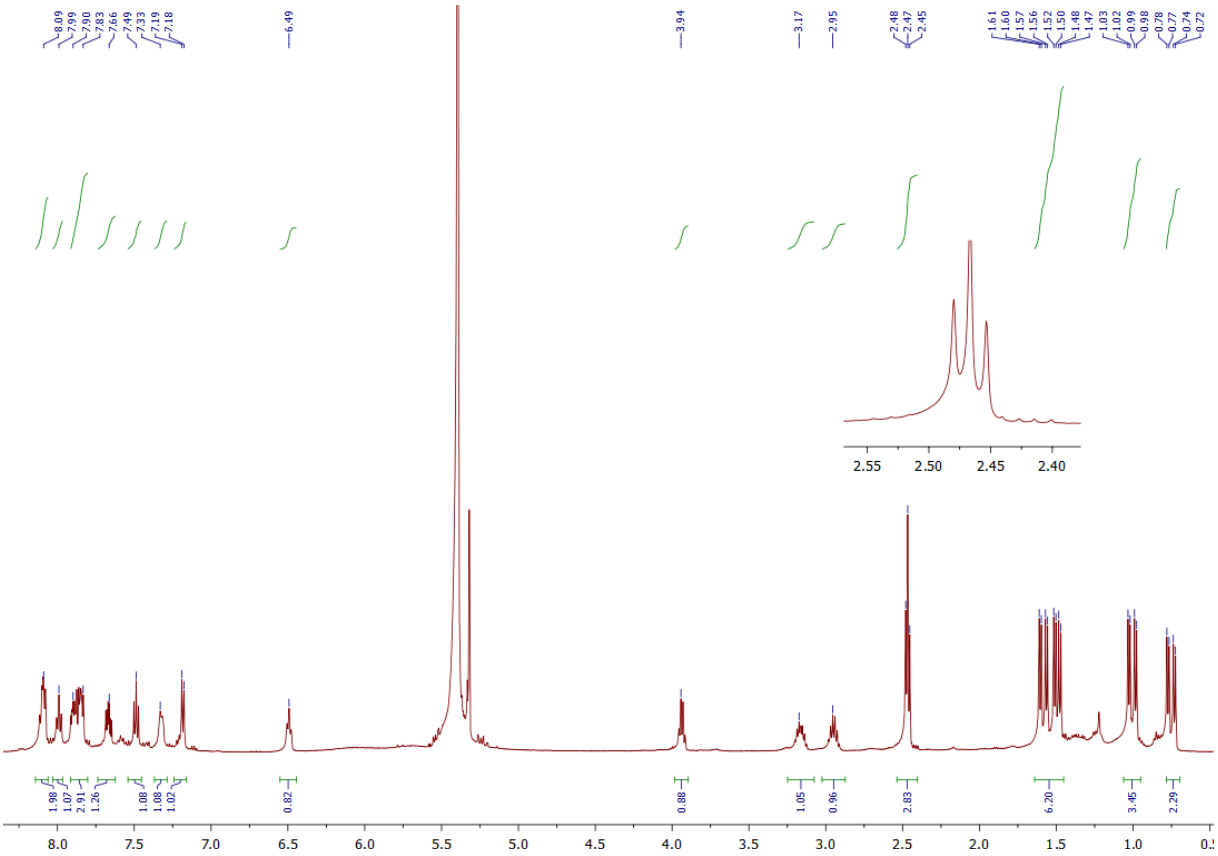

Figure S24. ^{31}P{^{1}H} NMR spectrum of complex **6** (273K)

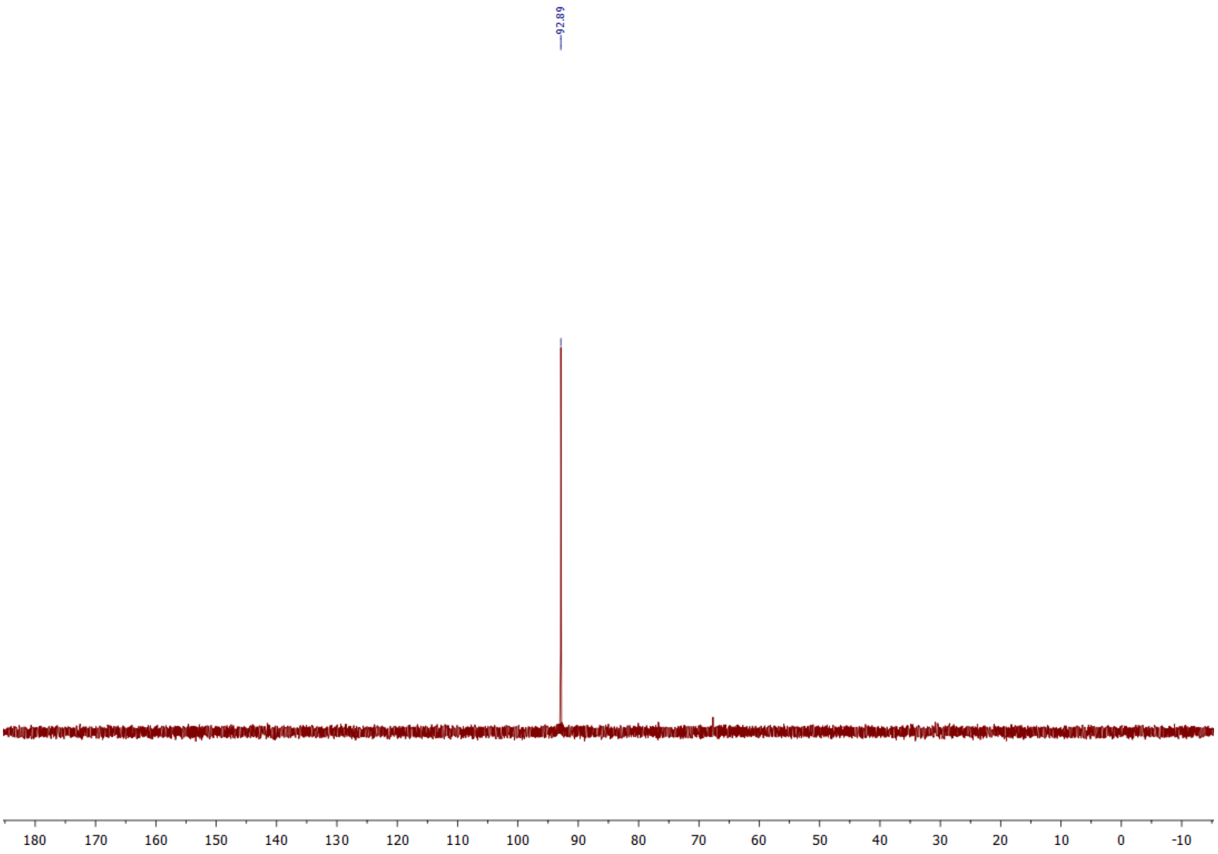

Figure S25. $^{13}C\{^{1}H\}$ NMR spectrum of complex **6** (273K)

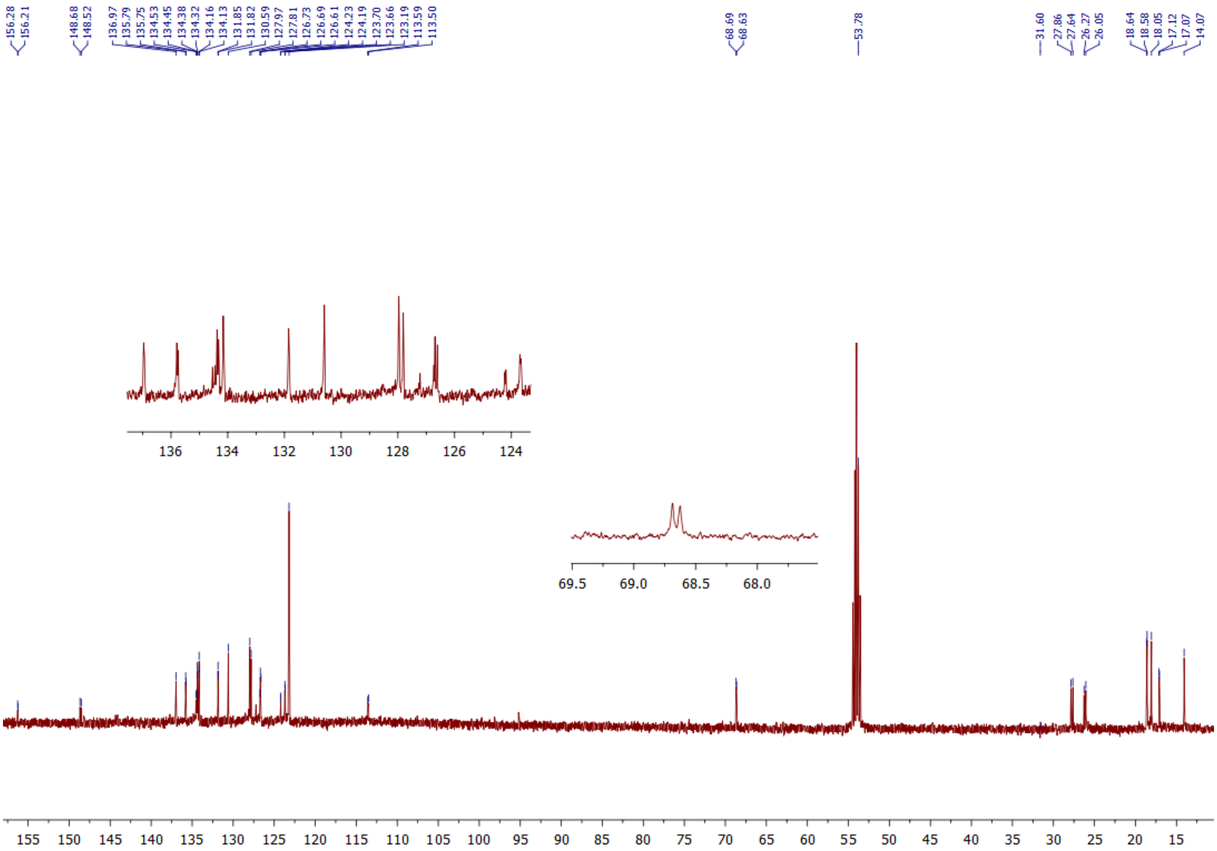

Crystallographic data

Crystallographic data of **2** and **4'** were collected at 193(2) K on diffractometers equipped with air-cooled microfocus sources: a Bruker-AXS PHOTON100 D8 VENTURE diffractometer with CuK_α radiation, λ= 1.54178 Å for **2** and a Bruker-AXS APEX II Quazar diffractometer with MoK_α radiation, λ = 0.71073Å for **4'**. Phi- and omega-scans were used. Space groups were determined on the basis of systematic absences and intensity statistics. Empirical absorption correction was employed.[2] The structures were solved by direct methods[3] and refined using the least-squares method on F^2. All non-H atoms were refined with anisotropic displacement parameters. Hydrogen atoms were refined isotropically at calculated positions using a riding model except the H atom on C9 for **4'** which was located in difference Fourier maps and refined with the isotropic displacement parameter U_{iso}(H) = 1,2 U_{eq}(C).

Crystallographic data have been deposited to the Cambridge Crystallographic Data Centre as supplementary publication no. CCDC-1526613 (**2**) and 1538015 (**4'**). These data can be obtained free of charge via www.ccdc.cam.uk/conts/retrieving.html (or from the CCDC, 12 Union Road, Cambridge CB2 1EZ, UK; fax: (+44) 1223-336-033; or deposit@ccdc.cam.ac.uk)

Crystal data for **2**: $C_{22}H_{25}AuIP$, *M* = 644.26, monoclinic, space group $P2_1$, a = 8.4290(3) Å, b = 10.7662(3) Å, c = 11.6292(3) Å, β = 94.266(2) °, V = 1052.41(5) Å^3; Z=2, crystal size 0.200 x 0.100 x 0.050 mm^3, 3003 reflections collected (2196 independent, Rint=0.0593), 231 parameters, 1 restraints, R1 [I>2σ(I)]= 0.0406, *wR2* [all data]= 0.1155, largest diff. peak and hole: 1.558 and -1.107 e.Å^{-3}.

Crystal date for **4'**: $C_{30}H_{37}AuF_6OPSb$, *M* = 877.28, monoclinic, space group $P2_1/c$, a = 12.9887(7) Å, b = 15.9169(9) Å, c = 15.3964(7) Å, β = 106.6993(18) °, V = 3048.8(3) Å^3, Z=4, crystal size 0.400 x 0.160 x 0.120 mm^3, 51528 reflections collected (7564 independent, Rint= 0.0317), 459 parameters, 252 restraints, R1 [I>2σ(I)]= 0.0191, *wR2* [all data]= 0.0463, largest diff. peak and hole: 0.773 and -0.922 e.Å^{-3}.

---

[2] Bruker, *SADABS*, Bruker AXS Inc., Madison, Wisconsin, USA.
[3] G. M. Sheldrick, *Acta Cryst. A* **2008**, *64*, 112–122.

Computional details

All calculations were performed using the Gaussian 09 package[4] and the B3PW91 hybrid functional on the real experimental systems.[5] The weakly coordinating counter–anion SbF_6^- has not been considered in the calculations since we previously showed that even a more coordinating counter-anion like NTf_2^- has no significant impact on the reaction profile (migratory insertion of norbornene and ethylene, β-hydride elimination) in similar processes than those described in this work.[6] The gold atom was described with the relativistic electron core potential SDD and associated basis set,[7] augmented by a set of f-orbital polarization functions.[8] The 6-31G** basis set were employed for other atoms. All stationary points involved were fully optimized. Frequency calculations were undertaken to confirm the nature of the stationary points, yielding one imaginary frequency for transition states (TS), corresponding to the expected process, and all of them positive for *minima*. The connectivity of the transition states and their adjacent *minima* was confirmed by intrinsic reaction coordinate (IRC)[9] calculations.

4 Gaussian 09, Revision C.01, M. J. Frisch, G. W. Trucks, H. B. Schlegel, G. E. Scuseria, M. A. Robb, J. R. Cheeseman, G. Scalmani, V. Barone, B. Mennucci, G. A. Petersson, H. Nakatsuji, M. Caricato, X. Li, H. P. Hratchian, A. F. Izmaylov, J. Bloino, G. Zheng, J. L. Sonnenberg, M. Hada, M. Ehara, K. Toyota, R. Fukuda, J. Hasegawa, M. Ishida, T. Nakajima, Y. Honda, O. Kitao, H. Nakai, T. Vreven, J. A. Montgomery, Jr., J. E. Peralta, F. Ogliaro, M. Bearpark, J. J. Heyd, E. Brothers, K. N. Kudin, V. N. Staroverov, T. Keith, R. Kobayashi, J. Normand, K. Raghavachari, A. Rendell, J. C. Burant, S. S. Iyengar, J. Tomasi, M. Cossi, N. Rega, J. M. Millam, M. Klene, J. E. Knox, J. B. Cross, V. Bakken, C. Adamo, J. Jaramillo, R. Gomperts, R. E. Stratmann, O. Yazyev, A. J. Austin, R. Cammi, C. Pomelli, J. W. Ochterski, R. L. Martin, K. Morokuma, V. G. Zakrzewski, G. A. Voth, P. Salvador, J. J. Dannenberg, S. Dapprich, A. D. Daniels, O. Farkas, J. B. Foresman, J. V. Ortiz, J. Cioslowski, and D. J. Fox, Gaussian, Inc., Wallingford CT, **2009**.

5 (a) A. D. Becke *J. Chem. Phys.*, **1993**, *98*, 5648; (b) J. P. Perdew, in *Electronic Structure of Solids '91*, Ed. P. Ziesche and H. Eschrig, Akademie Verlag, Berlin, **1991**, 11-20.

6 F. Rekhroukh, L. Estevez, S. Mallet-Ladeira, K. Miqueu, A. Amgoune, D. Bourissou *J. Am. Chem. Soc.*, **2016**, *138*, 11920.

7 Andrae, D. ; Häussermann, U.; Dolg, M.; Stoll, H.; Preuss, H. *Theor. Chim. Acta* **1990**, *77*, 123.

8 Ehlers, A. W.; Biihme, M.; Dapprich, S.; Gobbi, A.; Hijllwarth, A.; Jonas, V.; Kiihler, K. F.; Stegmann, R.; Veldkamp, A.; Frenking, G. *Chem. Phys. Letters*, **1993**, *208*, 111.

9 (a) Fukui, K. *Acc. Chem. Res.*, **1981**, *14*, 363; (b) Hratchian, H. P.; Schlegel, H. B. in *Theory and Applications of Computational Chemistry: The First 40 Years*, Ed. Dykstra, C. E.; Frenking, G.; Kim, K. S.; Scuseria, G. Elsevier, Amsterdam, **2005**, 195.

Natural Bond Orbital[10] calculations (NBO, 5.9 version)[11] have been carried to analyze the bonding situation, in particular for the description of π-interaction in arene gold complexes. Natural Localized Molecular Orbital (NLMO) were plotted with Molekel 4.3[12] and all the geometrical structures with Gaussview 5.0.[13]

Coupling constants J_{CP} and ^{13}C NMR chemical shifts were evaluated by employing the direct implementation of the Gauge Including Atomic Orbitals (GIAO),[14] with the IGLOII[15] basis set on C, H and P atoms, using as reference the corresponding $SiMe_4$ shielding constant calculated at the same level of theory.

10 (a) Reed, E.; Curtiss, L. A.; Weinhold, F. *Chem. Rev.* **1988**, *88*, 899. (b) Foster J. P.; Weinhold, F. *J. Am. Chem. Soc.* **1980**, *102*, 7211. (c) Reed A. E.; Weinhold, F. *J. Chem. Phys.* **1985**, *83*, 1736.

11 NBO 5.0 program, Glendening, E. D.; Badenhoop, J, K.; Reed, A. E.; Carpenter, J. E.; Bohmann, J. A.; Morales, C. M.; Weinhold, F. Theoretical Chemistry Institute, University of Wisconsin, Madison, **2001**.

12 MOLEKEL 4.3, Flükiger, P.; Lüthi, H. P.; Portmann, S.; Weber, J. Swiss Center for Scientific Computing, Manno (Switzerland), 2000-2002.

13 GaussView, Version 5, Dennington, R.; Keith, T.; Millam, J.. *Semichem Inc.*, Shawnee Mission, KS, **2009**.

14 (a) London, F. *J. Phys. Radium*, **1937**, *8*, 397. (b) McWeeny, R. Phys. Rev., **1962**, *126*, 1028. (c) Ditchfield, R. Mol. Phys., **1974**, *27*, 789. (d) Wolinski, K.; Hilton, J. F.; Pulay, P. *J. Am. Chem. Soc.* **1990**, *112*, 8251. (e) Cheeseman, J. R.; Trucks, G. W.; Keith, T. A.; Frisch, M. J. *J. Chem. Phys.* **1996**, *104*, 5497.

15 Kutzelnigg, W.; Fleischer, U.; Schindler M., *The IGLO-Method: Ab Initio Calculation and Interpretation of NMR Chemical Shifts and Magnetic Susceptibilities*, Springer-Verlag, Heidelberg, **1990**, vol. 23.

Figure S26. DFT optimized structures and relative energy stability (in kcal.mol^{-1}) for the *cis* and *trans* isomers of complex **4**, computed at the B3PW91/SDD+f(Au),6-31G**(other atoms) level of theory. The transition state for the *trans* - *cis* isomerization is also shown. Structure ***Cis*-4i** corresponds to a local minimum with π-coordination of $C_iC_{o'}$ (instead of C_iC_o in ***Cis*-4**). ΔE values are given in brackets.

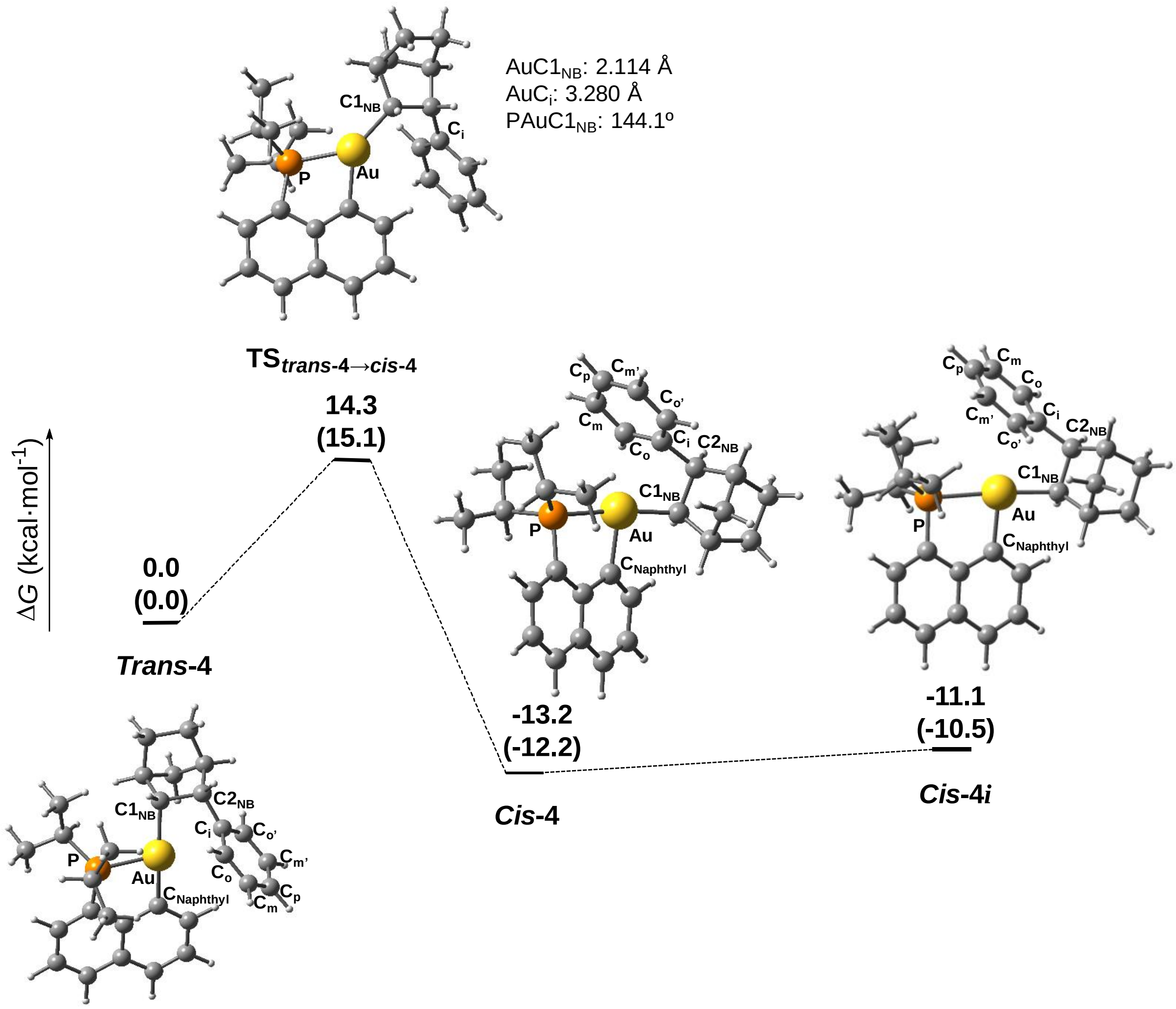

Table S1. Main geometrical parameters with distances in Å and bond angles in (°) for compounds *trans*-**4** and *cis*-**4**. Computed NMR data for complex ***cis*-4** (^{13}C NMR chemical shifts in ppm and J_{PC} coupling constants in Hz).

	***Trans*-4**	***Cis*-4**
**AuC_{NB}**	2.14	2.11 (J_{PCNB}: 75.9; δC_{NB}: 76.2)
**AuC_i**	2.53	2.61 (J_{PCi}: 12.7; δC_i: 128.8)
**AuC_o**	2.67	2.62 (δC_o: 127.0)
**C_iC_o**	1.42	1.417
**$AuC_{o'}$**	3.34	3.47 ($\delta C_{o'}$: 142.3)
**AuC_m**	3.58	3.54 (δC_m: 140.1)
**$AuC_{m'}$**	4.11	4.19 ($\delta C_{m'}$: 139.6)
**AuC_p**	4.22	4.24 (δC_p: 144.5)
**$PAuC_{NB}$**	100.7	171.7
**$PAuC_i$**	161.1	121.0
**$PAuC_{NB}$**	83.2	82.9

Figure S27. a) NLMO plot of π_{Ci-Co} (cutoff 0.05 au) associated with the π-arene interaction in complex ***cis*–4**. Stabilizing energy ΔE(2) found at the second order perturbation theory is around 30.7 kcal/mol. Atomic contributions of main atoms in the corresponding π_{C-C} NLMO are shown as well. b) Superposition of the donor π_{Ci-Co} and acceptor σ^*_{CAr-Au} (chicken-wire) NBOs (cutoff: 0.07 au) outlining the π–arene interaction.

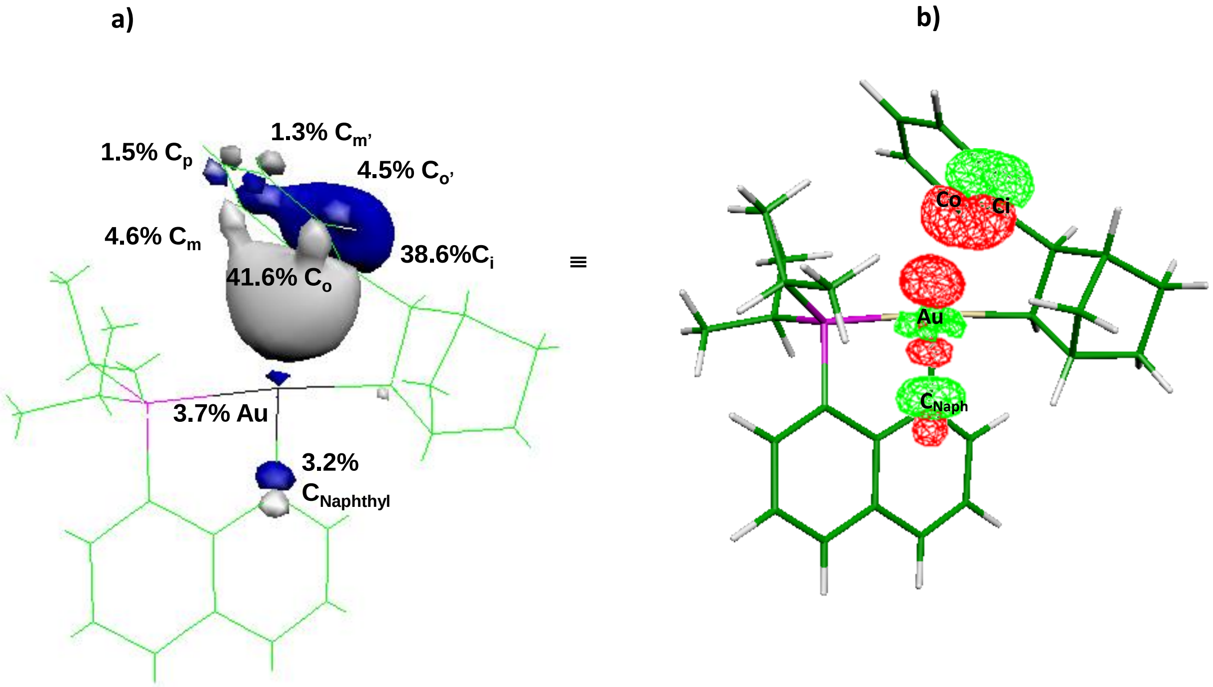

Figure S28. DFT optimized structures and relative energy stability (in kcal.mol^{-1}) for the *cis* and *trans* isomers of complex **4'**, computed at the B3PW91/SDD+f(Au),6-31G**(other atoms) level of theory.

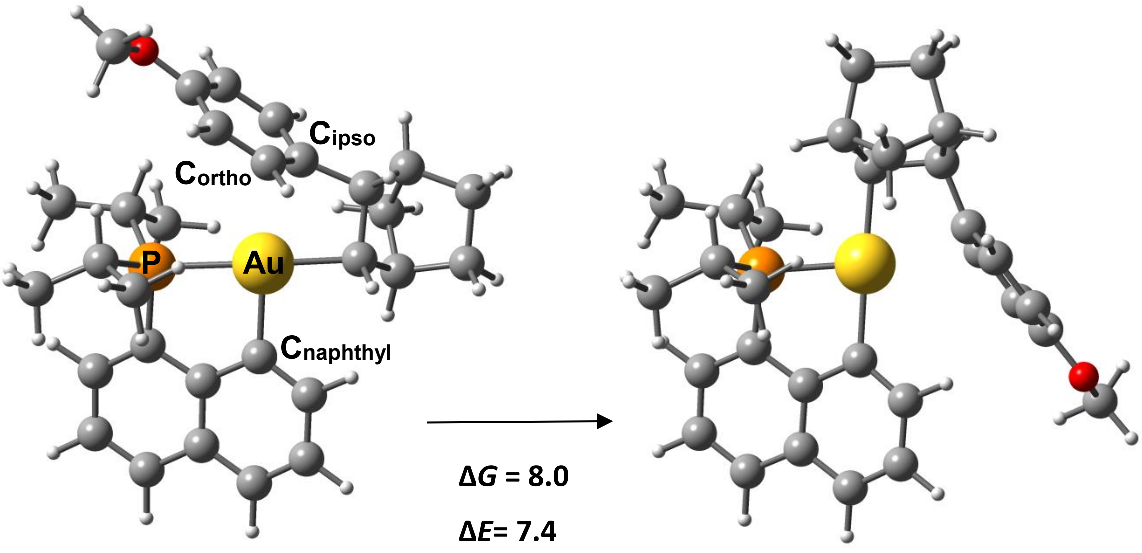

Cis-4' **Trans-4'**

Figure S29. a) NLMO plot of π_{Ci-Co} (cutoff 0.05 au) associated with the π-arene interaction in complex ***cis*–4'**. Stabilizing energy ΔE(2) found at the second order perturbation theory is 36.6 kcal/mol. Atomic contributions of main atoms in the corresponding π_{C-C} NLMO are shown as well.

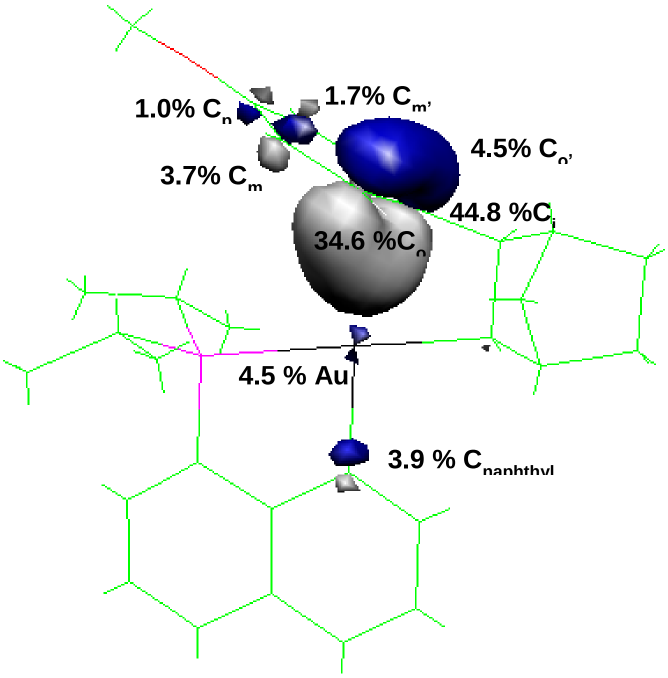

Table S2. Main geometrical parameters with distances in Å and bond angles in (°) and computed NMR data (main ^{13}C NMR chemical shifts in ppm and J_{PC} coupling constants in Hz) for complex **cis-4'**.

	**Trans-4'**	**Cis-4'**
**AuC_{NB}**	2.12	2.10 (J_{PCNB}: 76.1; δC_{NB}: 65.5)
**AuC_i**	2.41	2.44 (J_{PCi}: 10.7; δC_i: 99.5)
**AuC_o**	2.78	2.80 (δC_o: 145.5)
**C_iC_o**	1.42	1.42
**$AuC_{o'}$**	3.14	3.14 ($\delta C_{o'}$: 147.9)
**AuC_m**	3.69	3.70 (δC_m: 116.0)
**$AuC_{m'}$**	3.96	3.95 ($\delta C_{m'}$: 128.3)
**AuC_p**	4.21	4.21 (δC_p: 172.8)
**$PAuC_{NB}$**	98.1	175.6
**$PAuC_i$**	163.9	113.4

Figure S30. a) DFT optimized structures and relative energy stability (Δ*G* in kcal.mol[-1]) for the *cis* and *trans* isomers of complex **6**, computed at the B3PW91/SDD+f(Au),6-31G**(other atoms) level of theory. b) Energy profile (Δ*G* (Δ*E*) values in kcal·mol[-1]) computed at B3PW91/SDD+f(Au)/6-31G** (other atoms) level of theory for the *trans* to *cis* isomerization of complex **6**.

a)

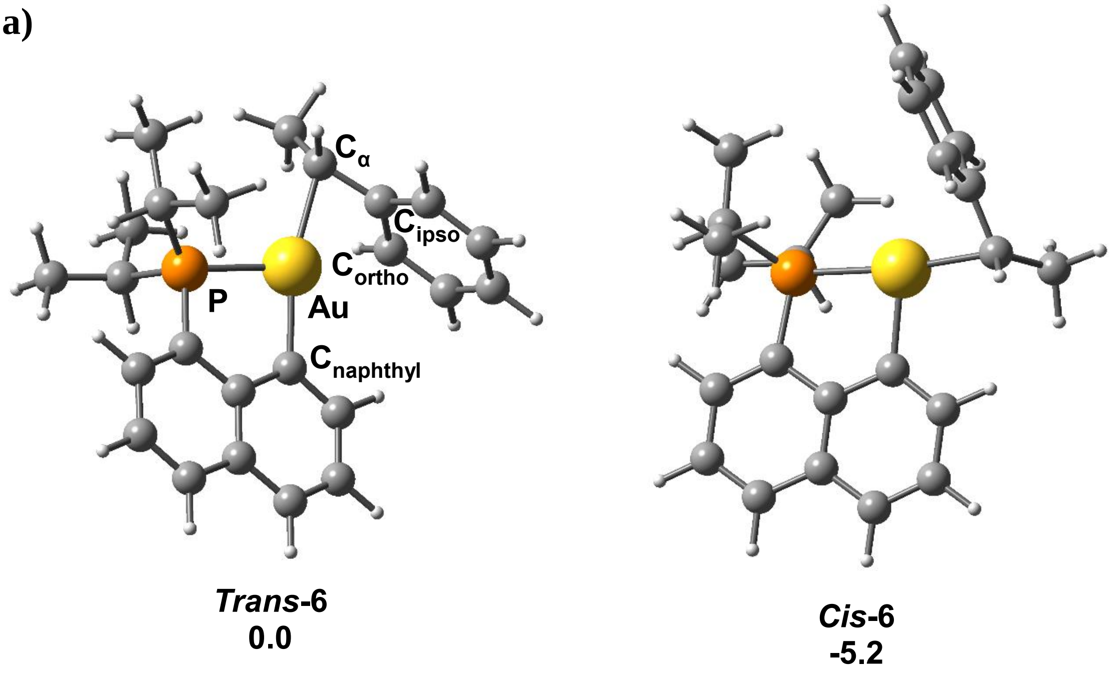

b)

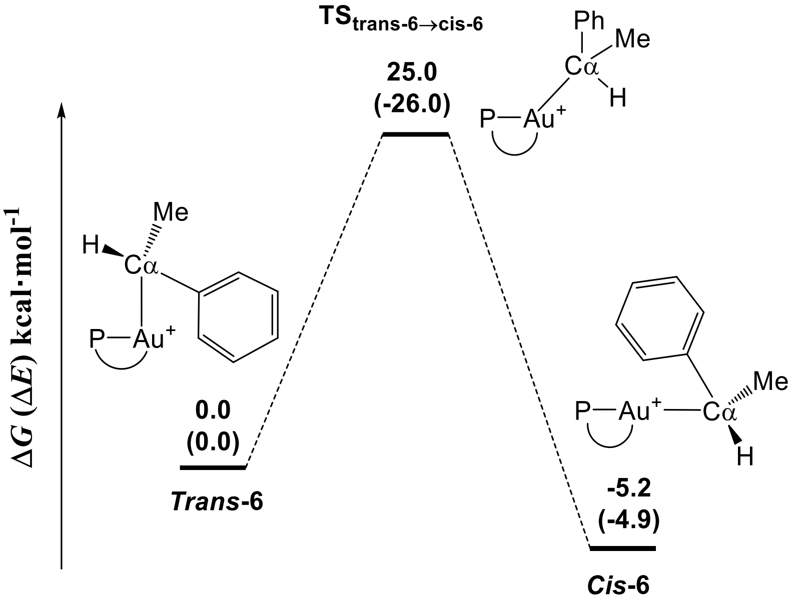

Figure S31. NLMO plot of $\pi_{Cipso\text{–}Cortho}$ (cutoff 0.05 au) orbital in complex *trans*–**6**, associated with the π-arene interaction. Stabilizing energy Δ*E*(2) found at the second order perturbation theory is around 38.6 kcal·mol^{-1} ($\pi_{Cipso\text{–}Cortho}\rightarrow\sigma^*_{PAu}$). Atomic contributions of main atoms in the corresponding $\pi_{Cipso\text{–}Cortho}$ NLMO are shown as well.

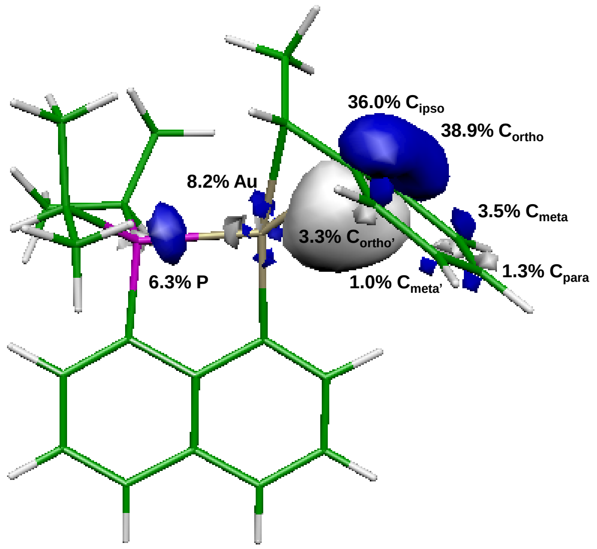

Table S3. Main geometrical parameters with distances in Å and bond angles in (°) and computed NMR data (main ^{13}C NMR chemical shifts in ppm and J_{PC} coupling constants in Hz) for complex ***trans***-**6**.

	***Trans*-6**
P-Au	2.321
Au-Cα	2.191 ($J_{PC\alpha}$: 13.0; δC_α: 67.5)
C_α-C_i	1.457
Au-C_i	2.277 (J_{PCi}: 5.0; δC_i: 124.6)
Au-C_o	2.546 (J_{PCo}: 10.7; δC_o: 120.6)
C_i-C_o	1.433
PAuC_α	109.4
PAuC_i	145.9
$C_{naphthyl}$AuC_α	166.3
$C_{naphthyl}$AuC_i	128.4

Figure S32. Energy profile (Δ*G* (Δ*E*) values in kcal·mol^{-1}) computed at B3PW91/SDD+f(Au)/6-31G** (other atoms) level of theory for the reaction of the cationic complex **3** with ethylene: a) migratory insertion and trans→cis isomerization, b) β-hydride elimination, c) styrene rotation and d) re–insertion into the Au–H bond.

a) Insertion

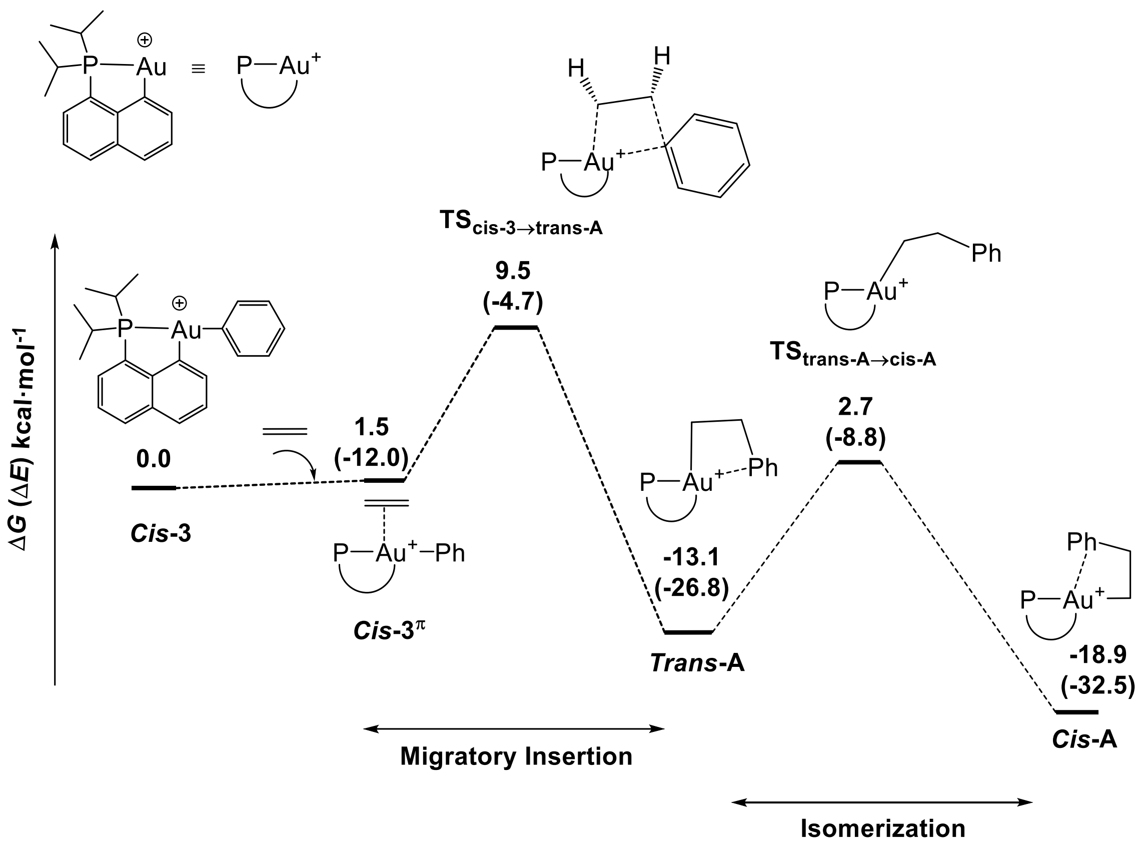

b) *β*-H elimination

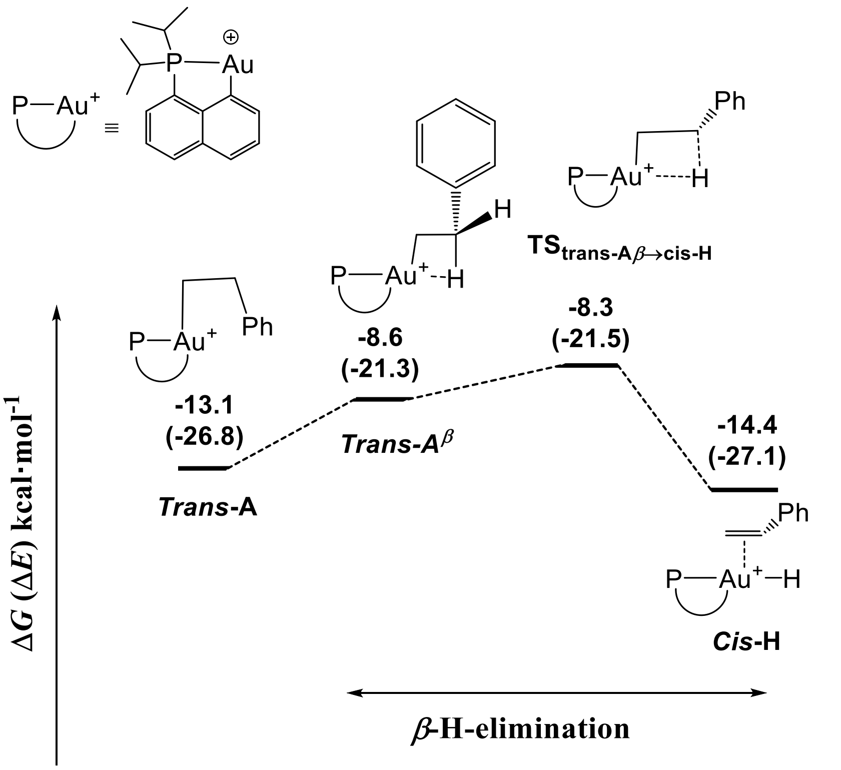

c) Olefin rotation

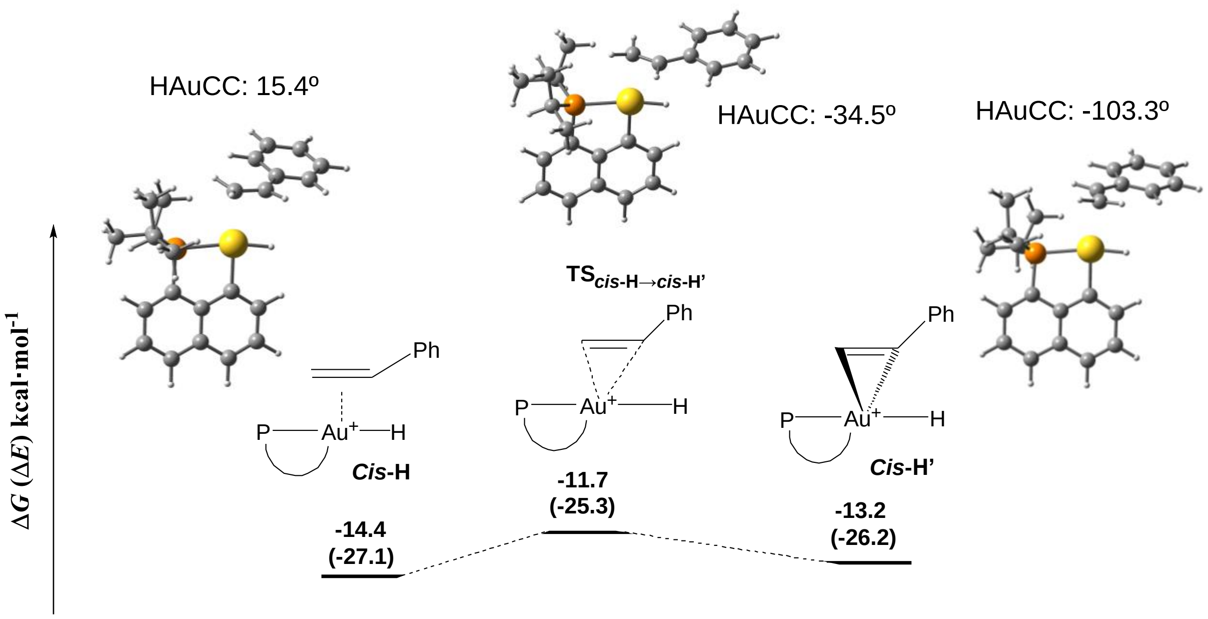

d) Re–insertion into the Au–H bond.

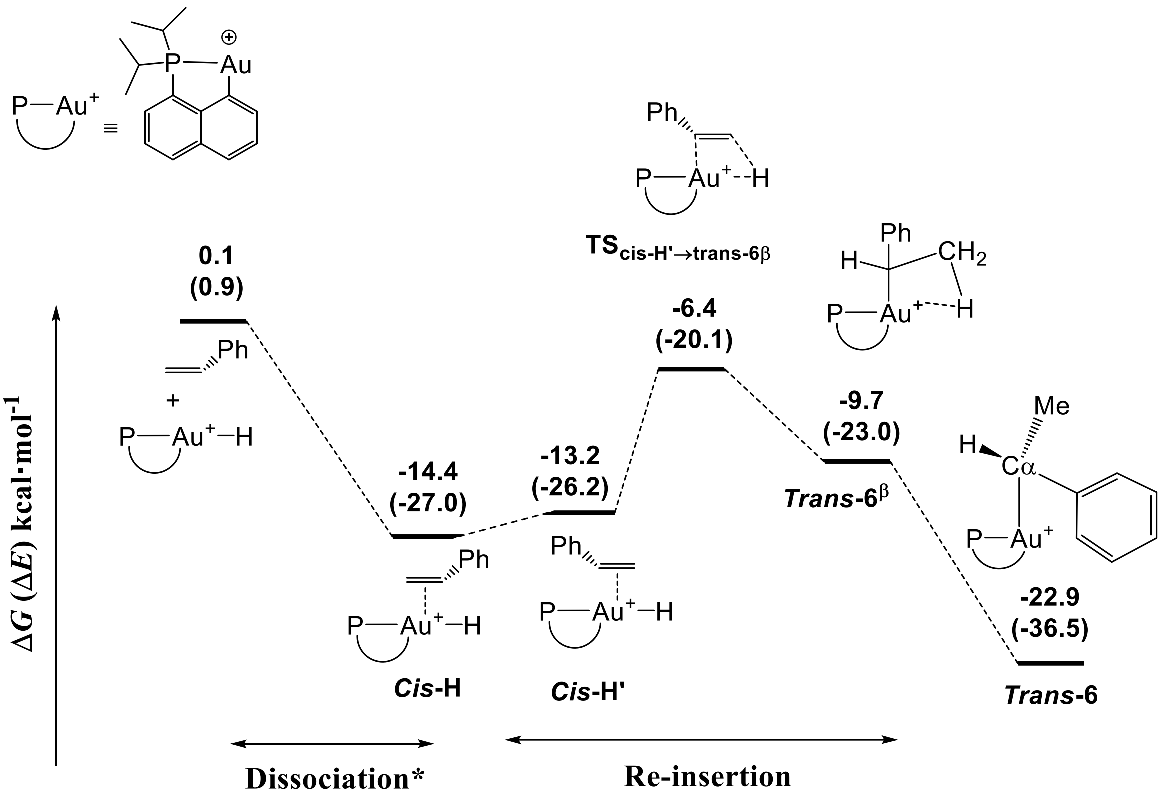

*Dissociation of styrene from ***Cis*-H** is thermodynamically disfavored. The corresponding transition state could not be located, even imposing geometrical constraints, but the activation barrier for the dissociation will be at least 14.5 kcal·mol^{-1}.

Figure S33. NLMO plot of π_{Ci-Co} (cutoff 0.05 au) associated with the π-arene interaction in complex ***trans*–A** (Au-C_i: 2.589 Å; AuC_o: 2.555 Å; PAuC_i: 159.2º; PAuCo: 159.5º). Stabilizing energy ΔE(2) found at the second order perturbation theory is around 10.5 kcal/mol. Atomic contributions of main atoms in the corresponding π_{Ci-Co} NLMO are shown as well.

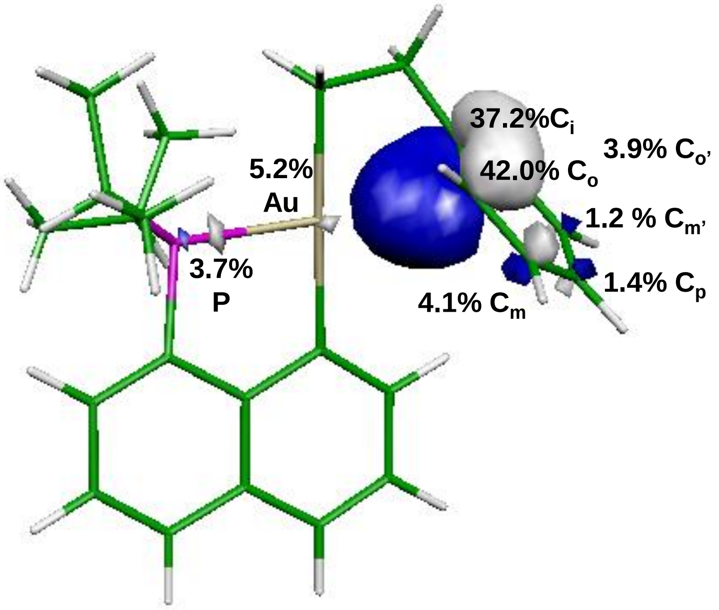

Figure S34. Energy profile (ΔG (ΔE) values in kcal·mol^{-1}) computed at the B3PW91/SDD+f(Au)/6-31G** (other atoms) level of theory for the β-hydride elimination from *trans*-**6**

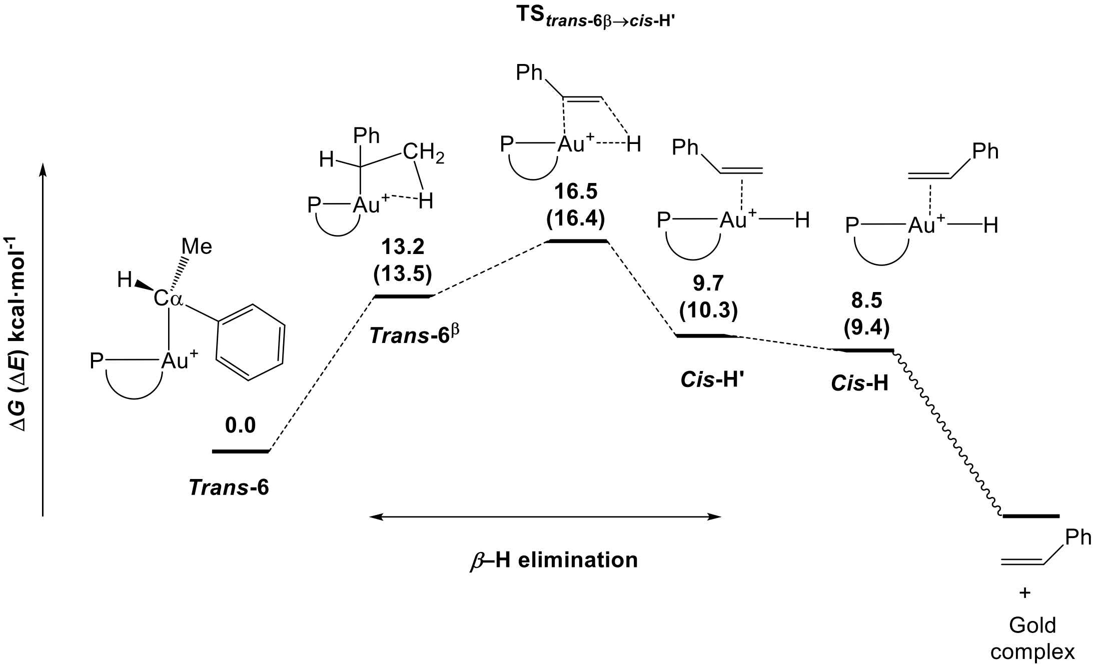

XYZ coordinates (Energy in au)

Cis-3

P	1.54482200	-1.02361000	-0.01338400
Au	-0.77155500	-0.36452300	-0.00852100
C	2.06581200	-1.82052100	-1.62030400
H	2.03395700	-0.96723300	-2.31154000
C	-0.04821400	1.54796000	-0.11590700
C	1.36691500	1.70468500	-0.04893800
C	-0.90954500	2.61312000	-0.22719200
C	2.29274900	0.62504100	0.04606700
C	1.87351000	3.04742000	-0.07479000
C	-0.37716900	3.92504500	-0.27070000
H	-1.97981100	2.47307500	-0.27687600
C	3.64863200	0.87066300	0.15206900
C	3.27363300	3.24802900	0.02416500
C	0.97506300	4.13648800	-0.19005800
H	-1.06512900	4.76002400	-0.35986900
C	4.14308500	2.19067100	0.14483500
H	4.35030000	0.04761300	0.24891900
H	3.64989300	4.26717800	0.00905100
H	1.37658000	5.14542200	-0.21195900
H	5.21068200	2.36502000	0.23030700
C	2.15171600	-1.98932500	1.45266900
H	3.24098500	-2.06050200	1.33048800
C	1.04317300	-2.85708100	-2.10264900
H	0.03323600	-2.43895700	-2.16971100
H	1.32130900	-3.19840200	-3.10465600
H	1.00513500	-3.73912000	-1.45747600
C	3.49141100	-2.37765100	-1.59935300
H	3.59080200	-3.22407000	-0.91307300
H	3.75090600	-2.73845500	-2.59964400
H	4.23200000	-1.62076500	-1.32842800
C	1.55987800	-3.40216900	1.47938200
H	0.46642700	-3.37924000	1.54632200
H	1.83854400	-3.99485300	0.60512200
H	1.92828600	-3.93124600	2.36363000
C	1.84466900	-1.22512900	2.74459900
H	0.76495900	-1.13589600	2.90707500
H	2.26046100	-1.77270600	3.59597100
H	2.27732900	-0.22157200	2.74865700
C	-2.75573200	-0.00631000	-0.00005100
C	-3.48525700	-0.06727800	-1.20021900
C	-3.43784600	0.05929900	1.22750400
C	-4.87654600	-0.13795000	-1.16363400
H	-2.97583800	-0.07027500	-2.16054200
C	-4.82954000	-0.01341900	1.25299000
H	-2.89167900	0.15743600	2.16228700
C	-5.54649300	-0.11036900	0.06017100
H	-5.43731900	-0.20525600	-2.09137200
H	-5.35346800	0.01742000	2.20389900
H	-6.63148100	-0.15042100	0.08339100

Sum of electronic and zero-point Energies= -1329.636921
Sum of electronic and thermal Free Energies= -1329.693840

Cis-$3_{NB}\pi$

P	1.53155600	-1.28160700	0.16933600
Au	-0.42184300	0.17031300	0.15370700
C	-2.57616100	-0.97542400	0.62501400
H	-2.73663500	-0.58732900	1.62573100
C	-1.68816300	-1.97763200	0.29676800
H	-1.09080400	-2.51471600	1.02342000
C	1.52529700	-2.63561200	-1.15205500
H	0.69134500	-2.31948600	-1.79076700
C	0.98876500	1.67114900	-0.13108900
C	2.35770200	1.28696400	-0.31454600
C	0.66293800	3.01147700	-0.16383800
C	2.80517700	-0.06084100	-0.23873200
C	3.34372300	2.30427800	-0.55052500
C	1.64018700	4.00524600	-0.40476000
H	-0.35486900	3.33901000	-0.00796200
C	4.14729100	-0.37823400	-0.35748600
C	4.70214800	1.93460400	-0.70486700
C	2.95102100	3.66302800	-0.60306900
H	1.33161300	5.04599100	-0.42894300
C	5.10363600	0.62388900	-0.60425200
H	4.48232200	-1.40500300	-0.25413100
H	5.43209300	2.71783700	-0.89070700
H	3.70520400	4.42242400	-0.78897200
H	6.15050200	0.35690300	-0.70684300
C	2.07276100	-1.95303900	1.84110900
H	2.94441900	-2.58320300	1.61411300
C	1.21262500	-4.03035700	-0.59953100
H	0.25422700	-4.08825900	-0.07997200
H	1.17078300	-4.73853200	-1.43326200
H	1.99538600	-4.38043700	0.08001300
C	2.77610800	-2.69292300	-2.03524100
H	3.64458800	-3.07653200	-1.49156500
H	2.58108600	-3.38801000	-2.85861200
H	3.03586300	-1.72625700	-2.46939200
C	1.02278300	-2.79973100	2.56684600
H	0.16117200	-2.19115900	2.86222400
H	0.67235000	-3.65645400	1.98943400
H	1.46263600	-3.19313500	3.48881900
C	2.51896700	-0.79884900	2.74801600
H	1.69705500	-0.09988700	2.93746200
H	2.83203700	-1.20843500	3.71357400
H	3.35577500	-0.23555500	2.33360800
C	-2.16759500	-2.57447000	-1.01196900
H	-1.44799700	-3.16151600	-1.58234000
C	-3.61741500	-0.92731100	-0.46695400
H	-4.20145800	-0.01136500	-0.53313400
C	-2.79658800	-1.35127900	-1.69572900
H	-2.06191900	-0.60137200	-2.00630400
H	-3.42118300	-1.61784800	-2.55271900
C	-3.45375400	-3.36210900	-0.59506900
H	-3.81093300	-3.94579000	-1.44844100
H	-3.26852500	-4.06248100	0.22418700
C	-4.44743200	-2.23104200	-0.21948100
H	-5.32082300	-2.22855700	-0.87760200
H	-4.81501400	-2.30360600	0.80758200
C	-1.90851700	1.63076200	0.15863500
C	-2.43746100	2.12544200	-1.03647800
C	-2.34027600	2.16688800	1.37559700
C	-3.39402200	3.14323000	-1.01121400
H	-2.09992700	1.74440300	-1.99632500
C	-3.29560600	3.18569800	1.39519000
H	-1.93027700	1.81099100	2.31810100
C	-3.82585200	3.67423000	0.20266700
H	-3.79443100	3.52380900	-1.94693300
H	-3.62027100	3.59709600	2.34697500
H	-4.56684500	4.46766100	0.21906700

Sum of electronic and zero-point Energies= -1602.151718
Sum of electronic and thermal Free Energies= -1602.213859

TScis-$3_{NB}\pi$→trans-4

P	1.58054300	-1.27481900	0.19610900
Au	-0.38820700	0.02735200	0.13177800
C	-2.89720200	-0.58282000	0.39175600
H	-3.36052900	-0.31960100	1.33603400
C	-1.85709400	-1.58281800	0.33103900
H	-1.62725100	-2.12771300	1.24586300
C	1.58978000	-2.75205800	-0.96594900
H	0.90051800	-2.42959100	-1.75673600
C	0.93927500	1.60971500	-0.18737200
C	2.31618500	1.29026200	-0.41473100
C	0.56061400	2.93521400	-0.22970200
C	2.80933800	-0.04389000	-0.31900000

C	3.25481700	2.33345700	-0.70874000
C	1.48378100	3.96643200	-0.52433000
H	-0.46827300	3.21797100	-0.03891300
C	4.15707500	-0.32050000	-0.47604600
C	4.61823100	2.00509900	-0.90704000
C	2.80099900	3.67393500	-0.76991500
H	1.13727100	4.99539500	-0.55511000
C	5.06507200	0.70992700	-0.78803500
H	4.53737300	-1.32790200	-0.34741000
H	5.31690700	2.80451700	-1.13945200
H	3.51358000	4.46126900	-0.99959200
H	6.11613800	0.47530800	-0.92300600
C	2.07136000	-1.80982700	1.92635500
H	2.84788100	-2.57145500	1.76650000
C	1.02587900	-4.01376100	-0.30294100
H	0.04379100	-3.86152800	0.14848700
H	0.92229900	-4.79683100	-1.06068600
H	1.70263600	-4.39800200	0.46623900
C	2.93854100	-3.05912000	-1.62252100
H	3.68571000	-3.38668100	-0.89216700
H	2.79787500	-3.88490000	-2.32772300
H	3.33628700	-2.21018300	-2.18029600
C	0.91478900	-2.42583700	2.71957800
H	0.15073900	-1.67112700	2.93334900
H	0.43883100	-3.27032700	2.21937500
H	1.29436300	-2.78619800	3.68095100
C	2.67696600	-0.63998200	2.71015800
H	1.96083900	0.18157700	2.81713700
H	2.93723400	-0.98624800	3.71538000
H	3.58001700	-0.24324100	2.24468600
C	-2.16340000	-2.38178400	-0.94293200
H	-1.34256300	-2.97294400	-1.34852500
C	-3.75571300	-0.78235300	-0.83328500
H	-4.39642400	0.05574400	-1.10596800
C	-2.73795200	-1.29487500	-1.86479100
H	-1.99883800	-0.54667000	-2.17085900
H	-3.21645300	-1.70357200	-2.75928000
C	-3.43981100	-3.20190300	-0.62257100
H	-3.66012800	-3.89275800	-1.44249400
H	-3.33625600	-3.79853800	0.28838900
C	-4.52700000	-2.10129600	-0.50390400
H	-5.32165100	-2.23366400	-1.24391900
H	-5.00709500	-2.07005500	0.47861900
C	-2.11221200	1.34534800	0.25112600
C	-2.57386000	2.02058800	-0.89301200
C	-2.29060000	1.95827800	1.50819200
C	-3.20093000	3.25906200	-0.78226100
H	-2.44524300	1.58348900	-1.87826400
C	-2.90800700	3.19971300	1.61627700
H	-1.95560100	1.45340900	2.41187000
C	-3.37023400	3.85054000	0.47021200
H	-3.55419500	3.76474900	-1.67620800
H	-3.03714000	3.65559000	2.59349200
H	-3.86459000	4.81367100	0.55448500

Sum of electronic and zero-point Energies= -1602.140663
Sum of electronic and thermal Free Energies= -1602.200215

Trans-4

P	1.30555500	-1.42291600	0.26649000
Au	-0.36815000	0.16175800	0.08148900
C	-3.22002600	-0.00063400	0.30109800
H	-3.84010800	-0.13197300	1.19623400
C	-2.10447300	-1.08043900	0.18712100
H	-2.08939400	-1.76998600	1.03367200
C	1.04654800	-2.95100400	-0.78529500
H	0.52866000	-2.54653100	-1.66393800
C	1.24346700	1.44968500	-0.35876500
C	2.54357300	0.88031800	-0.51642000
C	1.09596000	2.79686400	-0.61518900
C	2.77166600	-0.50231500	-0.27335200
C	3.65751700	1.69252900	-0.90226700
C	2.18312100	3.60359300	-1.03163900
H	0.12633500	3.27035500	-0.50112000
C	4.04462000	-1.04444000	-0.34383100
C	4.94074600	1.10096900	-0.99716000
C	3.43974300	3.06749500	-1.16683100
H	2.01627100	4.65742800	-1.23646100
C	5.13516000	-0.23154100	-0.71186600
H	4.22756100	-2.08689500	-0.10782600
H	5.78232800	1.72305500	-1.29059000
H	4.27918900	3.68634600	-1.47139100
H	6.12784900	-0.66642900	-0.77028600
C	1.57443100	-1.88506500	2.06019000
H	2.20285900	-2.78626400	1.99106900
C	0.14528600	-3.98997200	-0.10972200
H	-0.80966800	-3.58085900	0.22292500
H	-0.07142700	-4.78376700	-0.83117900
H	0.64534300	-4.45812100	0.74342200
C	2.34428200	-3.60670000	-1.27025000
H	2.94022100	-4.00545100	-0.44276300
H	2.07495700	-4.45516400	-1.90756900
H	2.96178500	-2.93153200	-1.86362500
C	0.28649200	-2.22445200	2.81494000
H	-0.34065800	-1.33398800	2.92099800
H	-0.30624600	-3.00759900	2.34188800
H	0.54734100	-2.56784200	3.82086800
C	2.35304900	-0.78983600	2.79694900
H	1.79472400	0.15185200	2.81416100
H	2.50529700	-1.10566700	3.83371800
H	3.33122200	-0.59697400	2.35442800
C	-2.43105000	-1.77939700	-1.14792900
H	-1.60686100	-2.33611300	-1.59804300
C	-4.06036300	-0.23267500	-0.96835600
H	-4.71830400	0.60134200	-1.22658700
C	-2.99312700	-0.63005900	-2.00163000
H	-2.25792600	0.15471000	-2.21739400
H	-3.42125400	-0.97091200	-2.94947700
C	-3.69904300	-2.63627700	-0.92515200
H	-3.87755000	-3.28391200	-1.79013400
H	-3.61562800	-3.28432200	-0.04674800
C	-4.81679200	-1.56496200	-0.78119800
H	-5.57773600	-1.67934200	-1.55855000
H	-5.33216500	-1.61258300	0.18310500
C	-2.55986800	1.38114600	0.42077300
C	-2.75859000	2.42945900	-0.50741400
C	-1.89962900	1.69978700	1.63769000
C	-2.38938300	3.73102700	-0.19958900
H	-3.25573000	2.22557800	-1.44934400
C	-1.51300400	3.01054400	1.93356600
H	-1.81047100	0.93246800	2.40407800
C	-1.77970700	4.02834600	1.02617500
H	-2.58926800	4.52700500	-0.91075700
H	-1.04014000	3.23113800	2.88521500
H	-1.51453000	5.05381100	1.26511200

Sum of electronic and zero-point Energies= -1602.166796
Sum of electronic and thermal Free Energies= -1602.227260

TStrans-4→cis-4

P	-1.76081600	-1.20412800	0.39947000
C	-1.82589300	-1.22201300	2.25799500
C	-1.92653000	-2.88810000	-0.35091200
C	2.17775900	-0.61639500	-1.05908400
H	2.09960700	-0.51517200	-2.15018700
H	-2.94835700	-3.17585300	-0.05688500
H	-2.03225100	-0.16492300	2.47351000
C	-3.11726700	-0.15653100	-0.16931400
C	-2.58367600	1.12032300	-0.43330200
C	-4.47491100	-0.38967700	-0.25588800
C	-1.18565500	1.30911600	-0.38621700
C	-3.43362600	2.22089300	-0.73749800
C	-5.32503800	0.68507200	-0.60581500
H	-4.90196800	-1.36914700	-0.06250500
C	-0.61369500	2.54344400	-0.53597200
C	-2.83267100	3.48848500	-0.94210600
C	-4.82347500	1.95103100	-0.82957300
H	-6.39226900	0.50658200	-0.68983200
C	-1.46933800	3.64071800	-0.83293800
H	0.45913100	2.70623900	-0.45238100
H	-3.45952100	4.34303300	-1.17957500
H	-5.49899000	2.76096900	-1.09072500

H	-1.01511500	4.61615200	-0.97735500
Au	0.22064700	-0.21628000	-0.36786400
C	-1.86361100	-2.78418000	-1.87910400
H	-0.86044100	-2.50335500	-2.21428900
H	-2.10027500	-3.76037100	-2.31280800
H	-2.57730600	-2.05866700	-2.27856500
C	-0.94170800	-3.91950800	0.20409300
H	-1.10323300	-4.87206000	-0.30939300
H	0.09612700	-3.62376900	0.02820600
H	-1.07512800	-4.09824800	1.27305400
C	-3.00738200	-2.06449700	2.75489100
H	-3.09950900	-1.93256100	3.83750600
H	-3.95732400	-1.75586700	2.30980200
H	-2.85915200	-3.13189400	2.56808500
C	-0.50120100	-1.59908000	2.92713700
H	-0.61534300	-1.49572600	4.01070700
H	-0.20678400	-2.63140600	2.72455300
H	0.31283100	-0.94009300	2.61507800
C	3.33271700	0.31320200	-0.52454800
H	3.87679500	0.70200500	-1.39404400
C	3.97802500	-2.31266300	-1.56687700
H	4.24053300	-3.37570500	-1.53484700
H	3.82432700	-2.05185000	-2.61876700
C	4.26929900	-0.69237900	0.20905300
H	4.89074500	-0.22621400	0.97783800
C	2.71554000	-2.03947000	-0.71456800
H	1.97238700	-2.83855100	-0.79298000
C	3.32136700	-1.81101200	0.67599700
H	3.86408300	-2.68188600	1.05991000
H	2.58373800	-1.50291600	1.42361300
C	5.06619400	-1.43959800	-0.87706000
H	5.56956100	-0.75678700	-1.56839300
H	5.83901200	-2.06552400	-0.41933000
C	2.92023100	1.53353700	0.27131200
C	2.40010600	1.45818100	1.57406800
C	3.07803200	2.80773200	-0.29119700
C	2.04611600	2.60818000	2.27979200
H	2.30668400	0.49148700	2.06001400
C	2.71942900	3.96155200	0.40768300
H	3.50149300	2.89679200	-1.28904600
C	2.19817600	3.86582900	1.69755400
H	1.66788000	2.52258900	3.29493000
H	2.86344000	4.93572500	-0.05150800
H	1.93181600	4.76186700	2.25017800

Sum of electronic and zero-point Energies= -1602.142671
Sum of electronic and thermal Free Energies= -1602.204492

Cis-4

P	-1.72019400	-1.05652900	-0.09297300
Au	0.42130700	0.07050400	0.39468200
C	3.32138200	0.18099900	0.32839300
H	4.00526100	0.11992800	1.18423400
C	2.18886600	1.21609100	0.57459700
H	2.18958900	1.63712900	1.58434200
C	-2.66744000	-1.64769000	1.41274900
H	-2.79147600	-0.70763200	1.96806100
C	-0.77360500	1.73428200	0.51158600
C	-2.06448600	1.66215500	-0.09218100
C	-0.35635000	2.89679600	1.11833800
C	-2.62271600	0.44670700	-0.58432800
C	-2.84316800	2.86236200	-0.18419200
C	-1.16683200	4.05636000	1.08780300
H	0.60650500	2.95975300	1.61078000
C	-3.83662900	0.45281300	-1.24449000
C	-4.08101700	2.82320900	-0.87479000
C	-2.36486400	4.05079200	0.41939700
H	-0.81172500	4.95893700	1.57579100
C	-4.55571300	1.65418600	-1.41838300
H	-4.26251000	-0.46845200	-1.63047200
H	-4.65318700	3.74241900	-0.96632000
H	-2.96691600	4.95260000	0.35490400
H	-5.49913200	1.64148200	-1.95480100
C	-1.93185500	-2.24326800	-1.51234000
H	-3.00732300	-2.22346400	-1.73236300
C	-1.85454800	-2.60378700	2.28986900
H	-0.90432600	-2.15894200	2.59734600
H	-2.42298000	-2.83291700	3.19699100
H	-1.64345900	-3.55193100	1.78743800
C	-4.05928500	-2.19563500	1.08859200
H	-4.01363100	-3.13639300	0.53211600
H	-4.59249400	-2.39750300	2.02330000
H	-4.66171200	-1.48287400	0.52009700
C	-1.54066100	-3.68363200	-1.17999700
H	-0.48868200	-3.75822800	-0.89526900
H	-2.14628300	-4.10947000	-0.37670400
H	-1.69047400	-4.30862400	-2.06649900
C	-1.17237200	-1.71479100	-2.73368300
H	-0.09088200	-1.73928100	-2.56401600
H	-1.38707600	-2.34718100	-3.60108700
H	-1.45874100	-0.69067200	-2.98880300
C	2.39681500	2.25382800	-0.53773800
H	1.53093700	2.88469500	-0.74059600
C	4.05140800	0.76023000	-0.90033600
H	4.68950500	0.04048400	-1.41959700
C	2.89886400	1.38632700	-1.70407200
H	2.15416400	0.66538900	-2.06087900
H	3.24673100	1.97821200	-2.55615100
C	3.67688200	3.04557600	-0.17460600
H	3.77356000	3.91805800	-0.82877000
H	3.66018900	3.41615900	0.85526200
C	4.81329900	2.01580900	-0.43035100
H	5.49140100	2.36066700	-1.21626000
H	5.42305500	1.82139100	0.45752900
C	2.69744500	-1.20295600	0.16205600
C	2.84723700	-1.99465800	-0.99404100
C	2.05714200	-1.78364100	1.28423300
C	2.45888200	-3.32747200	-0.99596800
H	3.32534100	-1.57619600	-1.87275900
C	1.66504800	-3.13231800	1.27209200
H	2.03080200	-1.23489500	2.22465500
C	1.88457500	-3.90662500	0.14276000
H	2.62766400	-3.93252000	-1.88208800
H	1.23654100	-3.57365400	2.16612300
H	1.62360500	-4.96063800	0.14339800

Sum of electronic and zero-point Energies= -1602.186313
Sum of electronic and thermal Free Energies= -1602.248192

Cis-3$^{\pi}$

P	-1.78974800	-0.63513300	0.04733700
Au	0.64211700	-0.57379900	-0.03951400
C	1.77213000	-2.73360800	-0.22897800
H	2.48172000	-2.57656800	-1.03340500
C	0.45137800	-2.95733900	-0.48067300
H	0.08339100	-2.98909800	-1.50167600
C	-2.36072800	-0.96036000	1.80168700
H	-1.93277700	-0.09237800	2.32196800
C	0.33718100	1.46653100	0.18056100
C	-0.98755400	1.96927900	-0.02914300
C	1.35480000	2.35309100	0.46487500
C	-2.11646100	1.12476000	-0.22341800
C	-1.20259200	3.38736800	-0.03289400
C	1.12262600	3.74753400	0.50502200
H	2.36074200	2.00217200	0.64861600
C	-3.36804900	1.65131800	-0.48542000
C	-2.49865200	3.88901800	-0.31210600
C	-0.12059200	4.25846200	0.23815000
H	1.95366000	4.40901300	0.73000200
C	-3.55693700	3.04606700	-0.55150700
H	-4.22490400	1.00275200	-0.64114100
H	-2.64118300	4.96596800	-0.33614300
H	-0.29427100	5.33074200	0.23702000
H	-4.54124400	3.44570200	-0.77294700
C	-2.86758400	-1.53335100	-1.18264300
H	-3.84967400	-1.06605700	-1.02938700
C	-1.75074500	-2.22210200	2.41961900
H	-0.65686900	-2.20428800	2.38797800
H	-2.04239100	-2.28400000	3.47267900
H	-2.10131100	-3.13884600	1.93588400
C	-3.88240200	-0.90829800	1.96137800
H	-4.38086400	-1.74553700	1.46411400

H	-4.13264200	-0.96752300	3.02542500
H	-4.30415400	0.02582000	1.58242000
C	-3.04037900	-3.03620700	-0.94768600
H	-2.12221500	-3.59817800	-1.13046700
H	-3.39335900	-3.27003700	0.05898700
H	-3.78987200	-3.41625200	-1.64907400
C	-2.40817600	-1.21980200	-2.61053200
H	-1.42678300	-1.65536300	-2.82471200
H	-3.11878400	-1.64790500	-3.32433300
H	-2.35094700	-0.14489600	-2.80009400
H	-0.21108300	-3.31917600	0.29811800
H	2.19180200	-2.87230300	0.76261700
C	2.69901200	-0.21542800	-0.04371000
C	3.43874800	-0.29613900	1.13854000
C	3.33041700	0.12794500	-1.24122200
C	4.80829800	-0.01965300	1.12110900
H	2.96178700	-0.55641000	2.08036400
C	4.70063800	0.39946200	-1.25024300
H	2.76716400	0.20385000	-2.16783500
C	5.44009700	0.32803400	-0.07124500
H	5.37757700	-0.07807400	2.04463000
H	5.18532600	0.67199100	-2.18358100
H	6.50433500	0.54249200	-0.08157300

Sum of electronic and zero-point Energies= -1408.166265
Sum of electronic and thermal Free Energies= -1408.223841

TScis-3$^{\pi}$→trans-A

P	-1.80783000	-0.58991100	0.05986800
Au	0.54026700	-0.63770900	-0.05263400
C	2.18065800	-2.55905400	-0.36641100
H	2.74197000	-2.58324600	-1.29332700
C	0.78044400	-2.78785400	-0.40205300
H	0.33451500	-2.99654200	-1.37165600
C	-2.38993100	-0.96671500	1.79514600
H	-1.98856300	-0.10313400	2.34278100
C	0.35486500	1.42783100	0.15848500
C	-0.94804500	1.99290600	-0.01518600
C	1.41688500	2.27949200	0.37579500
C	-2.10467500	1.17808500	-0.18403400
C	-1.11881300	3.41419900	-0.02810800
C	1.24450900	3.68404200	0.40299800
H	2.41717400	1.88632300	0.52077700
C	-3.34859700	1.73409100	-0.41936700
C	-2.40894500	3.95072300	-0.26830800
C	0.00914400	4.24283800	0.18996400
H	2.10751400	4.31956700	0.57883300
C	-3.49698400	3.13512400	-0.47575800
H	-4.22412400	1.10740600	-0.56313700
H	-2.52606500	5.03091800	-0.29228000
H	-0.11981800	5.32156200	0.18594400
H	-4.47516600	3.56307500	-0.67034300
C	-2.80422200	-1.49974300	-1.21580900
H	-3.81434900	-1.08216100	-1.10126900
C	-1.76996700	-2.23331400	2.39206600
H	-0.67692400	-2.19217400	2.38269500
H	-2.08546200	-2.32793200	3.43580900
H	-2.08751000	-3.14205800	1.87319800
C	-3.91635300	-0.94406300	1.92095300
H	-4.38778400	-1.77506200	1.38826500
H	-4.18833300	-1.03854500	2.97694200
H	-4.34589800	-0.00646700	1.55965500
C	-2.88574700	-3.01185100	-0.99250700
H	-1.90522500	-3.49090700	-1.05290600
H	-3.33605200	-3.27271700	-0.03238300
H	-3.51115900	-3.45243800	-1.77526600
C	-2.29261000	-1.14992100	-2.61728900
H	-1.28873100	-1.55322600	-2.78657100
H	-2.95637600	-1.59102800	-3.36726000
H	-2.26199600	-0.07084900	-2.78956500
H	0.32872400	-3.31072000	0.43679800
H	2.74180400	-2.86550900	0.50889000
C	2.70969000	-0.56245400	-0.07006000
C	3.36619500	-0.44380100	1.16828900
C	3.33251500	-0.05752200	-1.22576000
C	4.60948700	0.17764200	1.24874700
H	2.90885600	-0.84320100	2.07042000
C	4.57406700	0.56392900	-1.14051500
H	2.84756300	-0.15196600	-2.19438000
C	5.21391700	0.68136900	0.09543700
H	5.10907900	0.26325600	2.20917800
H	5.04592300	0.95224600	-2.03820600
H	6.18785700	1.15738100	0.15812600

Sum of electronic and zero-point Energies= -1408.154554
Sum of electronic and thermal Free Energies= -1408.211044

trans-A

P	-1.57986500	-0.85319900	0.07002700
Au	0.66772500	-0.43083900	-0.09295200
C	2.80083700	-2.27544000	0.21602300
H	3.53191600	-2.92553200	-0.28332400
C	1.38288800	-2.42232800	-0.34383900
H	1.37665900	-2.68040900	-1.40776700
C	-2.01752000	-1.34429400	1.81327400
H	-1.82644600	-0.40309500	2.34633200
C	0.03000000	1.55756700	0.20192800
C	-1.35494900	1.84286200	0.00575200
C	0.86333700	2.60949100	0.52005300
C	-2.28651800	0.78873900	-0.20123300
C	-1.84353600	3.18567200	0.02781100
C	0.38563400	3.94211300	0.57957300
H	1.91675300	2.43370800	0.71648300
C	-3.62215700	1.04140900	-0.45681900
C	-3.21667700	3.41568000	-0.24083400
C	-0.93202000	4.23111900	0.31691500
H	1.07771300	4.74345000	0.82282500
C	-4.08204800	2.37478500	-0.49374500
H	-4.33043600	0.23511600	-0.62457200
H	-3.58331600	4.43880800	-0.24622900
H	-1.29048400	5.25647100	0.33817700
H	-5.12801900	2.57187600	-0.70579000
C	-2.29519200	-1.98366500	-1.21159500
H	-3.37433100	-1.80044900	-1.09721400
C	-1.11691300	-2.42660600	2.41213700
H	-0.06229500	-2.14105300	2.38085500
H	-1.39172200	-2.57103800	3.46162600
H	-1.22861100	-3.39053200	1.90910300
C	-3.50877800	-1.67901400	1.94025800
H	-3.76960900	-2.60747300	1.42384900
H	-3.74656000	-1.81757300	2.99947800
H	-4.14823900	-0.87489200	1.56757300
C	-2.03268800	-3.47457200	-0.99434000
H	-0.97573900	-3.71868000	-1.11796700
H	-2.35980200	-3.82740100	-0.01387300
H	-2.59017800	-4.04086600	-1.74689600
C	-1.87029100	-1.52160800	-2.60977500
H	-0.80289700	-1.69930300	-2.77404300
H	-2.42211200	-2.09503900	-3.36076700
H	-2.07624100	-0.46147400	-2.77886400
H	0.81003900	-3.17820100	0.19316500
H	2.82260900	-2.54275400	1.27707900
C	3.22165800	-0.82111600	0.07461900
C	3.91915200	-0.14023700	1.09214900
C	2.97758900	-0.13415700	-1.14408100
C	4.41442500	1.13718000	0.87655700
H	4.10832900	-0.64655300	2.03458800
C	3.47415500	1.16685300	-1.34660800
H	2.56718500	-0.67498100	-1.99428200
C	4.20068600	1.79195600	-0.34737600
H	4.98225000	1.63197500	1.65917700
H	3.30652700	1.66057100	-2.29853000
H	4.60828200	2.78523400	-0.50824800

Sum of electronic and zero-point Energies= -1408.189907
Sum of electronic and thermal Free Energies= -1408.247166

TStrans-A→cis-A

P	1.60731900	-1.21094000	-0.04140000
Au	-0.19710000	0.24526900	0.12436100
C	-3.18067400	0.26337600	-0.82071500
H	-2.99323200	-0.62652900	-1.43384500

C	-2.26114200	0.25137500	0.41575100
H	-2.48469200	1.12057400	1.05554400
C	1.58117200	-2.00532500	-1.72212600
H	1.97674700	-1.19488500	-2.34935500
C	1.44738200	1.48490400	-0.12202500
C	2.80500500	1.10956100	-0.02567700
C	1.07372900	2.79194700	-0.29538000
C	3.13276100	-0.25408300	0.10716600
C	3.82019500	2.10638600	-0.05788500
C	2.09036500	3.78633800	-0.33273600
H	0.03198600	3.09021000	-0.38982400
C	4.44487700	-0.65819900	0.24995500
C	5.16028900	1.66411000	0.08783600
C	3.42136400	3.45827700	-0.21335900
H	1.79205500	4.82302700	-0.45400000
C	5.46021200	0.32634900	0.24389000
H	4.71466900	-1.70305800	0.36907200
H	5.95965500	2.39989700	0.08505300
H	4.17791000	4.23705100	-0.23804800
H	6.49368600	0.01669500	0.36170700
C	1.48783100	-2.41748900	1.35555700
H	2.40849600	-3.00974400	1.23055400
C	0.18226000	-2.37007600	-2.22951400
H	-0.49168900	-1.50945000	-2.23654900
H	0.27071900	-2.72977800	-3.25926900
H	-0.28224800	-3.16577200	-1.64259400
C	2.55806600	-3.18788400	-1.77315200
H	2.21998800	-4.02646200	-1.15776300
H	2.61723500	-3.54345600	-2.80649300
H	3.57129700	-2.91067300	-1.47009200
C	0.27713000	-3.35145600	1.27128300
H	-0.66465700	-2.79730100	1.32116500
H	0.27466600	-3.96269000	0.36631200
H	0.30263500	-4.03378300	2.12611000
C	1.55518000	-1.67509200	2.69529600
H	0.65632100	-1.07223500	2.85923700
H	1.61422400	-2.40921900	3.50423700
H	2.42941500	-1.02349600	2.77041300
H	-2.51376700	-0.60660300	1.05857800
H	-2.94726900	1.12836400	-1.45307200
C	-4.64141800	0.30649900	-0.43407000
C	-5.29248300	1.52951900	-0.23894000
C	-5.36101300	-0.87397400	-0.21922600
C	-6.62489200	1.57345200	0.16548700
H	-4.75299700	2.45830200	-0.41448000
C	-6.69376600	-0.83502200	0.18490300
H	-4.87553200	-1.83487000	-0.37965100
C	-7.32916800	0.39000400	0.38034900
H	-7.11578200	2.53231000	0.30608100
H	-7.23909600	-1.76171300	0.34018200
H	-8.36918500	0.42216600	0.69124900

Sum of electronic and zero-point Energies= -1408.161183
Sum of electronic and thermal Free Energies= -1408.221916

Cis-A

P	-0.45170200	1.42176500	-0.08287500
Au	0.45604100	-0.84341800	-0.19063200
C	2.47438000	-2.77233600	0.55566200
H	3.16627000	-3.51033700	0.12851400
C	1.09619800	-2.83884900	-0.10223700
H	1.12157900	-3.21398900	-1.13016100
C	-0.66193800	2.25120200	-1.74788000
H	-1.36072900	1.56154300	-2.24138100
C	-1.51958800	-1.38915900	-0.20564900
C	-2.47745300	-0.40385300	0.17927500
C	-1.92279000	-2.65736700	-0.55333900
C	-2.14059200	0.96115200	0.40633700
C	-3.84582600	-0.80630200	0.32332400
C	-3.28714100	-3.02382800	-0.46706200
H	-1.21456800	-3.40672100	-0.88588800
C	-3.09533900	1.85303800	0.85709000
C	-4.79139600	0.14093400	0.79114100
C	-4.22157300	-2.13090400	-0.00810300
H	-3.57740700	-4.03207700	-0.74606400
C	-4.42416100	1.43420100	1.07551600
H	-2.83878900	2.89124100	1.04523100
H	-5.82192200	-0.17748500	0.92228500
H	-5.26224700	-2.42404800	0.09615400
H	-5.15694300	2.14489200	1.44399100
C	0.12729200	2.63189500	1.20512800
H	-0.67229300	3.38264600	1.25956100
C	0.63134200	2.27038300	-2.56704400
H	1.05488700	1.26794200	-2.67477200
H	0.41695900	2.65024600	-3.57096700
H	1.39266400	2.92016300	-2.12685100
C	-1.32361700	3.62872000	-1.66921400
H	-0.68469100	4.36511700	-1.17276100
H	-1.51393000	3.99480800	-2.68321700
H	-2.28587500	3.59589000	-1.15223700
C	1.43104300	3.34070700	0.83674400
H	2.24970800	2.62845800	0.70721500
H	1.34372300	3.93577000	-0.07534600
H	1.70868300	4.02368700	1.64606600
C	0.22908200	1.92255000	2.55990700
H	1.02543000	1.17086300	2.55332000
H	0.47066900	2.65427500	3.33737200
H	-0.70576600	1.43136900	2.84314300
H	0.38563500	-3.42183500	0.48151700
H	2.39472700	-3.00052300	1.62234800
C	3.05293300	-1.38036200	0.39703600
C	3.72942400	-0.74025100	1.44853800
C	2.95998700	-0.71333900	-0.84825900
C	4.35143200	0.48510300	1.24843100
H	3.80242100	-1.23685600	2.41215100
C	3.58524400	0.53488600	-1.03682600
H	2.59193900	-1.25038200	-1.72160400
C	4.28676600	1.12456300	0.00258100
H	4.90519400	0.94511700	2.06181600
H	3.55275600	1.00682400	-2.01362300
H	4.79791800	2.07025100	-0.14935700

Sum of electronic and zero-point Energies= -1408.198843
Sum of electronic and thermal Free Energies= -1408.256299

Trans-Aβ

P	1.44262900	1.18105300	0.19027700
Au	-0.40680000	-0.02060000	-0.46458600
C	-2.67850100	0.06278500	-1.21548600
H	-1.93390700	-0.89160000	-1.03162000
C	-1.93842400	1.34314100	-1.01060000
H	-1.70898000	1.90177800	-1.91598100
C	1.24433200	1.77207700	1.94646600
H	1.26494400	0.82051100	2.49481600
C	0.79615500	-1.68125600	-0.11629500
C	2.18742500	-1.44391700	0.10505800
C	0.35485500	-2.99116600	-0.16571800
C	2.70277800	-0.11850900	0.19737200
C	3.10208400	-2.53566600	0.22514500
C	1.25143300	-4.07733300	-0.02146700
H	-0.69654100	-3.21506200	-0.32487400
C	4.05755000	0.11944400	0.34408900
C	4.48290200	-2.25868000	0.38830100
C	2.59521900	-3.85789600	0.16052300
H	0.86729200	-5.09208000	-0.06780900
C	4.95369000	-0.96600500	0.43370100
H	4.45068100	1.13103000	0.38975600
H	5.17588900	-3.09196300	0.46900900
H	3.28467800	-4.69230700	0.25352900
H	6.01583300	-0.77322500	0.54521700
C	1.98716700	2.51467400	-0.97311700
H	2.98551800	2.78582000	-0.59910700
C	-0.09254600	2.46273300	2.22604300
H	-0.94269700	1.82344600	1.97583200
H	-0.15355700	2.69343400	3.29408600
H	-0.19817900	3.40361000	1.68072200
C	2.44355200	2.61636600	2.39423500
H	2.49013600	3.57446000	1.86854900
H	2.33930900	2.83514300	3.46137900
H	3.39404400	2.09370700	2.26347200
C	1.09607100	3.75793100	-0.94948700
H	0.06730300	3.52034900	-1.23423300

H	1.08026400	4.24963200	0.02564400
H	1.47997600	4.48277900	-1.67382800
C	2.13321200	1.93584200	-2.38466800
H	1.15969000	1.64933600	-2.79616200
H	2.56046600	2.69772600	-3.04352700
H	2.78985100	1.06256700	-2.41119700
H	-2.30243200	1.96933200	-0.19948100
H	-2.88813300	-0.13875300	-2.27045900
C	-3.83514000	-0.25129200	-0.30351300
C	-3.64527800	-0.49159300	1.06009900
C	-5.12849000	-0.26964300	-0.83269400
C	-4.73669200	-0.72932500	1.89103700
H	-2.63722700	-0.51103600	1.47367000
C	-6.21998300	-0.50265800	0.00137000
H	-5.28608900	-0.09424200	-1.89373300
C	-6.02674800	-0.73146900	1.36209000
H	-4.57971800	-0.92246600	2.94788000
H	-7.22202100	-0.51082800	-0.41638600
H	-6.87835800	-0.92102000	2.00809800

Sum of electronic and zero-point Energies= -1408.181031
Sum of electronic and thermal Free Energies= -1408.239876

TStrans-Aβ→cis-H

P	-1.51343400	-1.15021100	0.14322500
Au	0.43233300	0.01647200	-0.44630900
C	1.89060400	-1.48327400	-1.06504200
H	1.54971300	-1.93109800	-1.99489300
H	2.12366200	-2.19457100	-0.27897400
C	2.71397700	-0.30697000	-1.18773100
H	1.81119300	0.86955200	-0.86044200
H	2.82376000	0.07653700	-2.20347400
C	-2.14720300	-2.41305900	-1.05703700
H	-3.15997900	-2.64373000	-0.69794700
C	-1.41194800	-1.81804600	1.88323400
H	-1.39502300	-0.88878500	2.46856000
C	-0.69608400	1.69972100	-0.03718900
C	-2.10131900	1.52002800	0.15908800
C	-0.18489700	2.98502000	-0.02092700
C	-2.69454700	0.22322600	0.18865300
C	-2.95437700	2.65713200	0.32030300
C	-1.02299400	4.10847800	0.17071800
H	0.87734300	3.15660100	-0.16253800
C	-4.06268700	0.06369500	0.31064600
C	-4.35135000	2.45577300	0.45487400
C	-2.37811300	3.95182700	0.32652000
H	-0.58418600	5.10182000	0.17887200
C	-4.89763200	1.19342200	0.43605100
H	-4.51345700	-0.92460100	0.30797600
H	-4.99376200	3.32532100	0.56567700
H	-3.02492700	4.81551800	0.45270400
H	-5.97075500	1.05844400	0.52557700
C	-2.66012100	-2.61459700	2.27748900
H	-2.59309200	-2.87820600	3.33763400
H	-3.57864800	-2.03751000	2.14637000
H	-2.74711600	-3.54873000	1.71479700
C	-0.11752100	-2.58469500	2.16680100
H	-0.09369300	-2.86825600	3.22363400
H	-0.03839800	-3.50284200	1.57890500
H	0.76570800	-1.97085600	1.96958600
C	-2.25224900	-1.78701200	-2.45185600
H	-2.71738700	-2.50317200	-3.13615500
H	-2.85819400	-0.87736700	-2.45466300
H	-1.26252900	-1.54213400	-2.85175900
C	-1.32237200	-3.70175000	-1.06811000
H	-1.34260700	-4.22458800	-0.10926800
H	-1.73220400	-4.38320400	-1.82000200
H	-0.27874100	-3.50869300	-1.33310400
C	3.84891200	0.03006300	-0.29437400
C	4.83607900	0.89797700	-0.78077200
C	3.98063300	-0.50437700	0.99468300
C	5.94511300	1.21164800	-0.00116300
H	4.74089400	1.31937600	-1.77869300
C	5.08540400	-0.18378300	1.77416500
H	3.22256300	-1.17178800	1.39433900
C	6.07009800	0.67254800	1.27755600
H	6.70947300	1.87582700	-0.39191700
H	5.18222300	-0.60319300	2.77075100
H	6.93289000	0.91826500	1.88900400

Sum of electronic and zero-point Energies= -1408.181433
Sum of electronic and thermal Free Energies= -1408.239439

Cis-H

P	1.24289600	1.26327200	0.11301200
Au	-0.40987800	-0.36136200	-0.55957400
C	-1.92035100	1.22076400	-1.32261500
H	-1.44697800	1.47733900	-2.26685000
H	-1.96666200	2.01146000	-0.58095400
C	-2.84992000	0.19382000	-1.31588200
H	-1.29272800	-1.62449400	-0.92840800
H	-2.88623000	-0.43652500	-2.20314500
C	1.77122300	2.61635600	-1.04927700
H	2.70379800	3.00016700	-0.61376000
C	0.94781200	1.92962500	1.83705700
H	1.08648200	1.02357100	2.44240800
C	1.02443800	-1.72784400	-0.03141500
C	2.34192500	-1.24428000	0.24646600
C	0.77682500	-3.08748200	0.00167200
C	2.66316000	0.14408900	0.27199100
C	3.38917700	-2.18731300	0.50496900
C	1.80927900	-4.00753800	0.29544600
H	-0.21368800	-3.47346900	-0.20484000
C	3.96280200	0.56841600	0.47752000
C	4.70712400	-1.71112800	0.72002000
C	3.08878000	-3.57127500	0.52838100
H	1.57844000	-5.06831300	0.31621500
C	4.99525300	-0.36759300	0.69158300
H	4.20912900	1.62618300	0.47478600
H	5.49641800	-2.43564700	0.90148100
H	3.88787000	-4.27893700	0.73017200
H	6.01196300	-0.01975800	0.84357100
C	1.98862500	2.96308800	2.27630500
H	1.81924100	3.21801300	3.32729500
H	3.00880700	2.57994900	2.19803100
H	1.91811500	3.89101200	1.70091100
C	-0.48635500	2.42053100	2.05083600
H	-0.61765400	2.70629600	3.09935800
H	-0.72109100	3.30016300	1.44399800
H	-1.21683400	1.63619400	1.83156500
C	2.08685300	2.01790100	-2.42421100
H	2.48342300	2.79922400	-3.08009200
H	2.83090600	1.21937500	-2.36698100
H	1.18762800	1.61276800	-2.90026700
C	0.77815800	3.77686700	-1.14676100
H	0.58511400	4.25196800	-0.18218400
H	1.18969500	4.54392300	-1.81024900
H	-0.17772600	3.46147400	-1.57335700
C	-3.86569200	-0.09521900	-0.31506400
C	-4.65822800	-1.24436000	-0.48708900
C	-4.11500200	0.74595200	0.78516600
C	-5.66317100	-1.55366100	0.42069300
H	-4.47798100	-1.89510800	-1.33905400
C	-5.12026900	0.43549900	1.68959600
H	-3.53839800	1.65555500	0.92107100
C	-5.89281100	-0.71548300	1.51138400
H	-6.26878000	-2.44286600	0.27798100
H	-5.31314300	1.09333700	2.53119500
H	-6.68005600	-0.95269200	2.22059600

Sum of electronic and zero-point Energies= -1408.190159
Sum of electronic and thermal Free Energies= -1408.249244

TScis-H-cisH'

Au	-0.47956600	0.11242200	-0.01358800
C	-1.92614700	-1.71665800	-0.03366100
C	-2.83290500	-1.01762400	0.74225200
H	-1.66239300	1.15739800	-0.02232400
H	-2.65626300	-1.01943200	1.81865600
P	1.58533000	-1.15834500	-0.03628000
C	0.75904000	1.73608900	-0.15644400

C	2.16744000	1.52580500	-0.01982000
C	0.27177600	3.01671800	-0.33930600
C	2.74465300	0.23125800	0.13040200
C	3.04046500	2.66239600	-0.02541200
C	1.14646600	4.12666300	-0.37884700
H	-0.79121100	3.19337700	-0.44590600
C	4.10481100	0.08198000	0.32875500
C	4.43060500	2.46446900	0.16919200
C	2.49760600	3.95707400	-0.21387100
H	0.73091000	5.11900200	-0.52558700
C	4.95322000	1.20758600	0.35814000
H	4.54062000	-0.90249100	0.47045100
H	5.08195600	3.33426300	0.17476700
H	3.16869500	4.81126000	-0.22298600
H	6.01783200	1.07312800	0.52051800
C	1.98943600	-2.32451700	1.35903100
H	3.06466100	-2.51896800	1.24706400
C	1.97824700	-1.93745300	-1.69282700
H	2.13711200	-1.04727800	-2.31651400
C	1.75637300	-1.61927200	2.69923400
H	0.69418000	-1.39893100	2.85279300
H	2.07932000	-2.27119600	3.51698500
H	2.31372600	-0.68246400	2.77642000
C	1.25516000	-3.66618500	1.29536600
H	1.40270700	-4.19423100	0.35056000
H	1.62935500	-4.31551800	2.09325900
H	0.17981800	-3.55051600	1.46213200
C	0.79593300	-2.71531500	-2.27884200
H	-0.08392000	-2.07826000	-2.40375000
H	1.06845400	-3.09371800	-3.26914200
H	0.51466300	-3.57852200	-1.66835000
C	3.27028500	-2.75894700	-1.69497300
H	3.19042000	-3.65727500	-1.07607900
H	3.48356700	-3.08691000	-2.71734600
H	4.13172600	-2.17781800	-1.35798400
H	-1.30404300	-2.45811300	0.45425000
H	-2.11726200	-1.88177000	-1.09086000
C	-4.04223500	-0.33164500	0.33200000
C	-4.78801700	0.33913700	1.31920700
C	-4.51943900	-0.34577400	-0.99314900
C	-5.97463400	0.98418000	0.99384000
H	-4.42935700	0.34916600	2.34541300
C	-5.70313600	0.29950000	-1.31454500
H	-3.96867700	-0.86842300	-1.76905100
C	-6.43146300	0.96600200	-0.32325400
H	-6.54318200	1.49692900	1.76296600
H	-6.06891300	0.28187300	-2.33630200
H	-7.35996400	1.46645800	-0.58123000

Sum of electronic and zero-point Energies= -1408.187354
Sum of electronic and thermal Free Energies= -1408.244901

Cis-H’

P	-1.08540000	1.29855700	-0.10630900
Au	0.38986300	-0.49746500	0.59469700
C	2.61369300	0.82352000	0.79765000
H	2.32746300	1.79043700	0.38577600
C	2.01380300	0.44287500	1.97911700
H	1.14077600	-1.83074600	0.98709500
H	1.40665100	1.15936700	2.52448700
C	-1.43801200	2.70438100	1.05793300
H	-2.28946000	3.23797100	0.61535000
C	-0.77244600	1.93623800	-1.84076600
H	-1.04011700	1.05557700	-2.44000500
C	-1.14644100	-1.69641900	-0.02400400
C	-2.41637000	-1.08698700	-0.27503900
C	-1.01595600	-3.06959500	-0.11057700
C	-2.61074900	0.32445100	-0.25261900
C	-3.54347100	-1.92411300	-0.55842400
C	-2.12530200	-3.88252200	-0.43769600
H	-0.06320900	-3.54717900	0.08199900
C	-3.86886200	0.86958600	-0.42704000
C	-4.81607500	-1.32545000	-0.73920700
C	-3.36368200	-3.32685000	-0.63973800
H	-1.98849600	-4.95759800	-0.50342600
C	-4.98320400	0.03631400	-0.65686600
H	-4.01862800	1.94448800	-0.38623400
H	-5.66820100	-1.96972400	-0.93809500
H	-4.22255200	-3.95447300	-0.86001800
H	-5.96678400	0.47772800	-0.78143600
C	-1.69734500	3.08914300	-2.23971900
H	-1.54407900	3.32139500	-3.29841600
H	-2.75341700	2.83764900	-2.11567000
H	-1.48604500	4.00125100	-1.67327900
C	0.69909400	2.25662300	-2.11439300
H	0.81850000	2.52507400	-3.16902200
H	1.05685700	3.10634100	-1.52417800
H	1.34514500	1.39526000	-1.92220300
C	-1.86278200	2.15560100	2.42381800
H	-2.13238900	2.98566200	3.08422300
H	-2.72696200	1.49081700	2.34989800
H	-1.05048800	1.60114800	2.90576000
C	-0.26381800	3.68009700	1.17138300
H	0.00237800	4.13377200	0.21366100
H	-0.53055100	4.49293800	1.85405700
H	0.62862000	3.19614200	1.58168100
C	3.66876600	0.13327500	0.07049900
C	4.16787800	-1.12455500	0.46088600
C	4.23984100	0.77478800	-1.04383300
C	5.20344100	-1.71613500	-0.24579200
H	3.74473000	-1.64061700	1.31725000
C	5.28166700	0.18138800	-1.74697900
H	3.87320400	1.75283500	-1.34468700
C	5.76229300	-1.06539700	-1.35028500
H	5.58318600	-2.68515900	0.06231500
H	5.72001500	0.68996600	-2.59968200
H	6.57503600	-1.53243600	-1.89826300
H	2.38489500	-0.39805300	2.55474600

Sum of electronic and zero-point Energies= -1408.188794
Sum of electronic and thermal Free Energies= -1408.247319

TScis-H’→trans-6β

P	-0.51510500	1.30000200	0.05470500
Au	0.16126800	-0.93180700	0.43462500
C	2.30403600	-0.90171400	1.26011100
H	2.27327200	-0.24252100	2.12452600
C	1.82205000	-2.23102900	1.47187000
H	0.51734100	-2.52911000	0.75408100
H	1.45484200	-2.49068200	2.46211200
C	-0.27005400	2.53421000	1.41945600
H	-0.90675100	3.38255300	1.13208200
C	0.03540300	1.96043400	-1.60297900
H	-0.49569400	1.28260900	-2.28547800
C	-1.74527000	-1.39910700	-0.23186400
C	-2.68667800	-0.32880700	-0.31788700
C	-2.17010500	-2.68739000	-0.49794600
C	-2.30283800	1.02694900	-0.10212100
C	-4.05717900	-0.60849700	-0.61855100
C	-3.51647100	-2.95798000	-0.83878400
H	-1.47894300	-3.52227400	-0.44195900
C	-3.24183500	2.04175800	-0.11127800
C	-4.98980900	0.45936300	-0.62941000
C	-4.44320800	-1.94599000	-0.88331700
H	-3.81460600	-3.98150000	-1.04565100
C	-4.59890000	1.75082900	-0.36396900
H	-2.95393100	3.07246700	0.07427300
H	-6.03174700	0.23811800	-0.84476400
H	-5.48276100	-2.15737400	-1.11750300
H	-5.32771300	2.55497700	-0.36027700
C	-0.46315100	3.38691000	-1.85414000
H	-0.20513700	3.67555000	-2.87803400
H	-1.54793900	3.47284300	-1.75787900
H	0.00951400	4.11210500	-1.18455800
C	1.53631200	1.81382700	-1.85596200
H	1.75065100	2.11327600	-2.88668700
H	2.13474000	2.45005200	-1.19992600
H	1.87812400	0.78389900	-1.73139600
C	-0.79341300	1.95422600	2.73731000
H	-0.75166600	2.72273200	3.51528300
H	-1.82870200	1.61251200	2.65839300
H	-0.17781700	1.11209200	3.07200200

C	1.17244300	3.02659900	1.54246800
H	1.51632800	3.55358000	0.64974600
H	1.24383500	3.72336400	2.38348900
H	1.86188100	2.20059400	1.73815300
C	3.32420400	-0.59222100	0.24361000
C	3.47457300	-1.36075900	-0.92410600
C	4.20036300	0.48336900	0.45709100
C	4.46937600	-1.05778500	-1.84753500
H	2.80896000	-2.19777900	-1.12177000
C	5.19944400	0.77947000	-0.46433700
H	4.11167300	1.07597300	1.36429000
C	5.33511800	0.01271700	-1.62132300
H	4.57226800	-1.66097700	-2.74447500
H	5.87826100	1.60564700	-0.27566200
H	6.11512800	0.24349800	-2.34016800
H	2.29651400	-3.04982700	0.93338900

Sum of electronic and zero-point Energies= -1408.179137
Sum of electronic and thermal Free Energies= -1408.236475

Trans-6β

P	-0.55046600	1.29933500	0.02961600
Au	0.19442900	-0.85383500	0.42831800
C	2.21470500	-0.86248700	1.24713400
H	2.28185500	-0.26491800	2.15394700
C	1.78239000	-2.27674000	1.47918000
H	1.54551900	-2.49951600	2.51977200
C	-0.28046200	2.53024000	1.38787000
H	-0.92588800	3.37359000	1.10159400
C	-0.00320700	1.91825400	-1.64116600
H	-0.53562500	1.22433500	-2.30593300
C	-1.70789700	-1.40765700	-0.23401500
C	-2.68751500	-0.37294200	-0.30795200
C	-2.10183900	-2.70709600	-0.49302400
C	-2.33230900	0.99096300	-0.09816600
C	-4.05453600	-0.68517500	-0.58433300
C	-3.44801200	-3.01928500	-0.80269800
H	-1.38505400	-3.52360100	-0.45644800
C	-3.29023300	1.98875300	-0.09405300
C	-5.00998300	0.36238500	-0.58371800
C	-4.40615700	-2.03524800	-0.83493500
H	-3.72138700	-4.05168500	-1.00012100
C	-4.64305400	1.66414200	-0.32889100
H	-3.02316600	3.02634400	0.08495900
H	-6.05072400	0.11978600	-0.78148800
H	-5.44274700	-2.27943900	-1.04957100
H	-5.38974500	2.45164700	-0.31815100
C	-0.52014800	3.33726400	-1.90500500
H	-0.28172500	3.60905500	-2.93811600
H	-1.60404600	3.41557200	-1.79172800
H	-0.04096300	4.07686600	-1.25631900
C	1.49718800	1.78162000	-1.90109200
H	1.69931500	2.07184500	-2.93683100
H	2.09442500	2.43019100	-1.25655600
H	1.84883100	0.75616000	-1.76838200
C	-0.78588100	1.95436000	2.71448800
H	-0.73188700	2.72688200	3.48751600
H	-1.82241900	1.61324000	2.65202100
H	-0.16461900	1.11373100	3.04150000
C	1.16334800	3.02336900	1.48497300
H	1.49079900	3.55070800	0.58640700
H	1.24731500	3.72025000	2.32455400
H	1.85390600	2.19592900	1.66849500
C	3.27910300	-0.59072000	0.26240500
C	3.43309300	-1.37158500	-0.89847900
C	4.17682700	0.46890400	0.47439100
C	4.44062100	-1.09336500	-1.81643800
H	2.76178400	-2.20586300	-1.09133400
C	5.18740700	0.74191100	-0.44104700
H	4.09306600	1.06824500	1.37780900
C	5.32091200	-0.03476600	-1.59249300
H	4.54401300	-1.70880500	-2.70510600
H	5.88047200	1.55602700	-0.25090800
H	6.11268400	0.17620900	-2.30453100
H	2.44272400	-3.02725400	1.03811700
H	0.77423400	-2.58315600	0.93584600

Sum of electronic and zero-point Energies= -1408.183733
Sum of electronic and thermal Free Energies= -1408.241643

Trans 6

P	1.40481200	-1.03801500	0.03778100
Au	-0.74975500	-0.19026100	-0.12421200
C	-2.19426300	-1.78888100	0.27318600
H	-2.06364200	-2.23690500	1.25840100
C	1.77423200	-2.20226100	1.43077600
H	2.87066800	-2.28360500	1.41799100
C	2.00083300	-1.69127400	-1.60712300
H	2.06283400	-0.76182700	-2.18885200
C	0.24168900	1.62924300	-0.39125600
C	1.62295400	1.67672800	-0.03047300
C	-0.37941500	2.80536200	-0.76218300
C	2.33367600	0.49200100	0.31876300
C	2.32316300	2.92277000	0.00239600
C	0.31630800	4.03887200	-0.76190900
H	-1.42792000	2.80652100	-1.04550300
C	3.64996700	0.54192500	0.73992500
C	3.67492100	2.93838000	0.42962400
C	1.63385700	4.10119300	-0.37843400
H	-0.20704300	4.94390100	-1.05712600
C	4.32004700	1.78184900	0.80417700
H	4.18530800	-0.35950000	1.02450900
H	4.19939800	3.88958200	0.46593800
H	2.16156400	5.05070800	-0.36036700
H	5.35118800	1.81425100	1.14129000
C	3.40708700	-2.29135800	-1.50403700
H	3.76425000	-2.52780800	-2.51126400
H	4.12449100	-1.59519000	-1.06223000
H	3.41753700	-3.22198900	-0.92860100
C	1.01322600	-2.62645000	-2.30592900
H	1.40115900	-2.87567400	-3.29866500
H	0.87251300	-3.56584200	-1.76507000
H	0.03587600	-2.15615900	-2.44115100
C	1.34188600	-1.57844900	2.76150600
H	1.66497900	-2.22272300	3.58498300
H	1.77679800	-0.58822800	2.91848700
H	0.25206100	-1.48899700	2.81789700
C	1.17841700	-3.59754900	1.23349000
H	1.54957000	-4.09306400	0.33350500
H	1.44985300	-4.22553900	2.08772100
H	0.08606300	-3.56298600	1.18467600
C	-2.41337300	-2.81116400	-0.81988800
H	-1.62632200	-3.56767400	-0.80823100
H	-3.36776200	-3.32963700	-0.66153700
H	-2.44117100	-2.37898600	-1.82350100
C	-2.95535400	-0.54704800	0.31580200
C	-3.14624200	0.21809100	-0.88070100
C	-3.33660300	0.06348200	1.55148300
C	-3.77232700	1.47614100	-0.83617000
H	-2.94713400	-0.23683600	-1.84717600
C	-3.94699800	1.29826400	1.57103200
H	-3.17492700	-0.48571600	2.47480000
C	-4.17486000	2.00836400	0.37459300
H	-3.95406800	2.01187500	-1.76260400
H	-4.26285800	1.72660800	2.51731600
H	-4.67175400	2.97288600	0.40847900

Sum of electronic and zero-point Energies= -1408.205253
Sum of electronic and thermal Free Energies= -1408.262738

TStrans-6→cis-6

P	-1.90178500	-0.64790200	0.18311300
Au	0.42255800	-0.66200900	-0.11968500
C	2.44530600	-2.28581900	-1.80896200
H	2.28319900	-1.57206600	-2.62243000
C	2.26560600	-1.66858600	-0.42093100
H	2.28176900	-2.46617100	0.33455500
C	-2.32099100	-1.06990500	1.94583600
H	-2.09480500	-0.12180000	2.45243100
C	-0.03973600	1.35047800	0.02021600
C	-1.34099200	1.89004900	-0.10193100
C	1.07403900	2.13969900	0.13924100

C	-2.46369100	1.03731600	-0.13270300
C	-1.50105200	3.30395200	-0.18152200
C	0.89859000	3.54973500	0.06710700
H	2.07235100	1.73138200	0.26454700
C	-3.73730100	1.54532500	-0.29316400
C	-2.82050700	3.79625600	-0.35139700
C	-0.34287100	4.11688900	-0.09734700
H	1.78298300	4.17502000	0.14013000
C	-3.90267300	2.94371600	-0.41518200
H	-4.60917800	0.89906000	-0.32828300
H	-2.97089800	4.86871200	-0.43908600
H	-0.44601200	5.19635700	-0.15762700
H	-4.90053000	3.34770800	-0.55240400
C	-2.65655600	-1.81045800	-1.04339700
H	-3.73662900	-1.69758800	-0.86320400
C	-1.43699900	-2.16242600	2.55680700
H	-0.37402200	-1.91417500	2.49566700
H	-1.69209500	-2.26400200	3.61621600
H	-1.59065800	-3.13759800	2.08866600
C	-3.81822900	-1.36469200	2.09857400
H	-4.10209700	-2.30473700	1.61641000
H	-4.04893200	-1.46144000	3.16410800
H	-4.44644300	-0.56123300	1.70403100
C	-2.26251200	-3.27315000	-0.81229200
H	-1.18219100	-3.42097100	-0.90466100
H	-2.58204400	-3.64816000	0.16239400
H	-2.74430400	-3.89216100	-1.57496400
C	-2.33967100	-1.34353500	-2.46881100
H	-1.27210900	-1.44121300	-2.69025100
H	-2.88451700	-1.97295000	-3.17872700
H	-2.63619000	-0.30641300	-2.64498300
H	1.74424400	-3.11214100	-1.96188800
H	3.46005700	-2.68835500	-1.92507500
C	3.30319900	-0.64522900	-0.02229500
C	3.62716600	-0.45828200	1.33446700
C	3.94732200	0.16935100	-0.96839100
C	4.54188500	0.51101100	1.73142900
H	3.15699200	-1.09214300	2.08402000
C	4.86865800	1.13733900	-0.57053900
H	3.73609900	0.04251600	-2.02585900
C	5.16420500	1.32173000	0.77954200
H	4.78005600	0.62728300	2.78497900
H	5.36384500	1.74511500	-1.32287100
H	5.88359100	2.07480100	1.08639600

Sum of electronic and zero-point Energies= -1408.163851
Sum of electronic and thermal Free Energies= -1408.222916

Cis-6

P	-0.65592100	1.45919300	-0.12603800
Au	0.56621500	-0.60666600	-0.14632000
C	1.84766100	-2.29471000	-0.20698300
H	1.63816000	-2.97897500	0.61675500
C	1.92513200	-2.98738300	-1.55020500
H	0.99852200	-3.52246700	-1.77210900
C	-0.29587200	2.71044100	1.20081300
H	-1.11660900	3.43874700	1.15033200
C	-0.76286200	2.26326100	-1.81127600
H	-1.35073300	1.52006100	-2.36725500
C	-1.28309800	-1.49820600	-0.03807400
C	-2.40861500	-0.64098200	0.16399600
C	-1.45179200	-2.86397400	-0.08887900
C	-2.30549600	0.78192300	0.21438600
C	-3.70073000	-1.23689100	0.33775000
C	-2.73615900	-3.44005600	0.05603800
H	-0.60701800	-3.52693300	-0.23233600
C	-3.41847300	1.55998300	0.47434800
C	-4.81754800	-0.39969500	0.58597200
C	-3.83368000	-2.64593000	0.27112800
H	-2.83891500	-4.51991300	0.00744700
C	-4.68234100	0.96548800	0.66663200
H	-3.33458300	2.64082800	0.53980800
H	-5.79149700	-0.86195800	0.72213700
H	-4.81847700	-3.08636700	0.39834600
H	-5.54453100	1.59196200	0.87156100
C	-1.53414000	3.58566700	-1.81290400
H	-1.63744000	3.94060300	-2.84328200
H	-2.54272200	3.47686600	-1.40731300
H	-1.01313400	4.36690000	-1.25114500
C	0.60259600	2.39000400	-2.49342700
H	0.46194400	2.74169300	-3.52040100
H	1.25486700	3.10719000	-1.98809400
H	1.12345600	1.42948100	-2.53972400
C	-0.32927000	2.02652000	2.57149500
H	-0.18699400	2.77639400	3.35600000
H	-1.28019400	1.52130000	2.75946800
H	0.47614100	1.29016600	2.66620800
C	1.02850000	3.44006100	0.96414600
H	1.03104300	4.01638400	0.03594600
H	1.20511400	4.14274200	1.78475800
H	1.87140900	2.74205400	0.93946100
C	2.81201400	-1.24560000	0.15778200
C	3.16808300	-0.23557600	-0.78094900
C	3.28393200	-1.10487800	1.49349600
C	4.00876800	0.82399200	-0.40778200
H	2.88822100	-0.34774700	-1.82474000
C	4.12234500	-0.06414400	1.83936100
H	3.00648500	-1.85568700	2.22817700
C	4.49323800	0.90304900	0.88753700
H	4.29873100	1.56157900	-1.14943500
H	4.50727000	0.00253500	2.85249400
H	5.16551700	1.70735000	1.17005500
H	2.73573600	-3.72685600	-1.53877000
H	2.11490200	-2.30342500	-2.38085600

Sum of electronic and zero-point Energies= -1408.213165
Sum of electronic and thermal Free Energies= -1408.270998

Cis-4’

Au	-0.25573800	-0.54719400	-0.28193100
O	-2.94719900	4.10863500	0.08378100
C	-1.47532700	-2.23188600	-0.56714200
H	-1.31310200	-2.60746800	-1.58177400
C	-2.85910700	-1.56771400	-0.34078000
H	-3.52971200	-1.70318000	-1.19717900
C	-3.40216600	-2.32602500	0.88748100
H	-4.21305600	-1.80881800	1.40806600
C	-3.78414200	-3.74109700	0.40437100
H	-4.42498800	-3.71579700	-0.48248500
H	-4.33802800	-4.26831500	1.18663100
C	-2.40556700	-4.41302200	0.14050700
H	-2.26176500	-5.29184800	0.77725900
H	-2.28474300	-4.74341000	-0.89598900
C	-1.39567000	-3.30471600	0.52599400
H	-0.38919100	-3.67237100	0.73383300
C	-2.12741300	-2.62819300	1.69705100
H	-2.31149700	-3.30733200	2.53530800
H	-1.60452500	-1.74309600	2.07681200
C	-2.64339200	-0.05096000	-0.17117900
C	-2.50990600	0.74915600	-1.33359500
H	-2.41783900	0.25691700	-2.29951400
C	-2.59053600	2.13956300	-1.29747200
H	-2.51669600	2.70119200	-2.22133900
C	-2.81663900	2.78331600	-0.07366500
C	-2.92031100	2.01622900	1.10395900
H	-3.10556600	2.53408900	2.03975600
C	-2.83822500	0.63861100	1.05486000
H	-2.97251800	0.07299500	1.97081500
C	-2.98244300	4.94163200	-1.07074900
H	-2.03281600	4.90815200	-1.61713900
H	-3.14950300	5.95232900	-0.70043900
H	-3.80334700	4.65685800	-1.73746300
C	1.52983000	-1.53558800	-0.47715500
C	2.72338300	-0.85662600	-0.07660400
C	2.74472100	0.50682300	0.34788300
C	3.92647600	1.09598300	0.76258900
H	3.94194600	2.13359200	1.07853500
C	5.13241600	0.36688800	0.76874100
H	6.04656200	0.84319600	1.10855900

C	5.14854500	-0.93269600	0.32258400
H	6.07983600	-1.49230200	0.30006600
C	3.96522700	-1.57345300	-0.12227600
C	3.99067500	-2.90039000	-0.61767100
H	4.93880700	-3.42942300	-0.65225500
C	2.83828600	-3.49883500	-1.05768000
H	2.85885500	-4.50827000	-1.45724900
C	1.60193600	-2.81578800	-0.97826400
H	0.71147200	-3.33102200	-1.31739100
P	1.15639100	1.37407200	0.23082100
C	0.72484800	2.16597000	1.86413000
H	-0.34358300	2.38576300	1.73486700
C	1.43434300	3.47383500	2.22292900
H	1.04409300	3.83579100	3.18021700
H	1.26070000	4.26144100	1.48601100
H	2.51265400	3.34001700	2.34794000
C	0.86712300	1.12377200	2.97969300
H	1.91642600	0.87026700	3.15632900
H	0.33218900	0.19548800	2.75446200
H	0.45808800	1.52737900	3.91124200
C	1.27945200	2.66463200	-1.11584300
H	0.39664600	3.29619600	-0.94200300
C	2.53963600	3.53573300	-1.08646700
H	3.43156400	2.93824700	-1.29407000
H	2.68762900	4.05696500	-0.13909300
H	2.46463000	4.29600600	-1.87109400
C	1.14127600	1.98366000	-2.48343700
H	1.18229300	2.73774800	-3.27631500
H	0.19880400	1.43867000	-2.58213400
H	1.96036800	1.27861100	-2.65851000

Sum of electronic and zero-point Energies= -1716.643169
Sum of electronic and thermal Free Energies= -1716.707455

Trans-4’

Au	0.24061700	-0.28788300	0.03807700
O	4.04766600	3.54303100	-0.00943100
C	0.98087700	-2.20120400	0.59163600
H	0.62533700	-2.47048500	1.59188100
C	2.49851800	-1.88524600	0.53580600
H	3.01828600	-2.09947000	1.47703600
C	3.00713500	-2.83389900	-0.56809500
H	3.97077400	-2.54740500	-0.99845600
C	2.99972200	-4.25712800	0.02963500
H	3.51451700	-4.30255500	0.99433200
H	3.51429000	-4.94953500	-0.64306000
C	1.48415900	-4.59514000	0.13231200
H	1.22949500	-5.47262000	-0.47143400
H	1.16480200	-4.80499500	1.15811500
C	0.80747600	-3.32649200	-0.43913900
H	-0.22110500	-3.49471900	-0.77195100
C	1.80990300	-2.91749900	-1.53255900
H	1.94261200	-3.68237900	-2.30439400
H	1.55751400	-1.97025900	-2.02316400
C	2.63757300	-0.37221200	0.27267000
C	2.53453100	0.51265600	1.38253100
H	2.16613400	0.12015800	2.32793600
C	2.97542400	1.82797100	1.33323900
H	2.88608900	2.45482800	2.21204100
C	3.55694800	2.31120100	0.15227100
C	3.64513000	1.46970500	-0.97850800
H	4.09353400	1.87064300	-1.88181900
C	3.19547800	0.16922200	-0.92070300
H	3.31513400	-0.46807300	-1.79053500
C	4.02275800	4.45163100	1.08899800
H	2.99472000	4.65854800	1.40508700
H	4.48261600	5.36813600	0.72184700
H	4.60419600	4.06470500	1.93245500
C	-0.47256000	1.64445200	-0.46121900
C	-1.84704500	1.91893200	-0.17361000
C	-2.73672100	0.87660500	0.22183400
C	-4.05638700	1.13635600	0.54855000
H	-4.73114400	0.33733800	0.83731100
C	-4.54608100	2.45692900	0.48679800
H	-5.57825800	2.65702600	0.75627700
C	-3.72814900	3.47866100	0.05961900
H	-4.11776700	4.49062900	-0.01573600
C	-2.37677600	3.24275400	-0.29608500
C	-1.53027000	4.27175300	-0.77908800
H	-1.92642000	5.27834200	-0.88155500
C	-0.23399300	3.98560100	-1.13084100
H	0.40552000	4.76774400	-1.53097100
C	0.28843800	2.67865000	-0.96721900
H	1.32257000	2.50610900	-1.24856900
P	-2.01353900	-0.77710700	0.11451400
C	-2.37944400	-1.82446300	1.60597100
H	-1.64305500	-2.63292500	1.50445600
C	-2.06573400	-1.03462600	2.88075800
H	-2.76313500	-0.20350400	3.01727100
H	-1.05060900	-0.62551500	2.87308300
H	-2.15404100	-1.69617100	3.74790700
C	-2.60106600	-1.63132300	-1.43482600
H	-2.40156000	-2.69414300	-1.24112200
C	-4.10279600	-1.43250700	-1.67932400
H	-4.32466300	-0.39016900	-1.92225000
H	-4.72581300	-1.72739200	-0.83253500
H	-4.40055300	-2.04649900	-2.53516600
C	-1.79178700	-1.18103100	-2.65533200
H	-2.16605400	-1.69841300	-3.54437300
H	-0.72856400	-1.41283600	-2.55429100
H	-1.89334800	-0.10515200	-2.82607200
C	-3.77352400	-2.46005400	1.65167800
H	-3.83415500	-3.09520900	2.54130600
H	-4.56871200	-1.71460900	1.73567800
H	-3.97661900	-3.09560500	0.78652300

Sum of electronic and zero-point Energies= -1716.631304
Sum of electronic and thermal Free Energies= -1716.694770
